# Global relationships in tree functional traits

**DOI:** 10.1038/s41467-022-30888-2

**Published:** 2022-06-08

**Authors:** Daniel S. Maynard, Lalasia Bialic-Murphy, Constantin M. Zohner, Colin Averill, Johan van den Hoogen, Haozhi Ma, Lidong Mo, Gabriel Reuben Smith, Alicia T. R. Acosta, Isabelle Aubin, Erika Berenguer, Coline C. F. Boonman, Jane A. Catford, Bruno E. L. Cerabolini, Arildo S. Dias, Andrés González-Melo, Peter Hietz, Christopher H. Lusk, Akira S. Mori, Ülo Niinemets, Valério D. Pillar, Bruno X. Pinho, Julieta A. Rosell, Frank M. Schurr, Serge N. Sheremetev, Ana Carolina da Silva, Ênio Sosinski, Peter M. van Bodegom, Evan Weiher, Gerhard Bönisch, Jens Kattge, Thomas W. Crowther

**Affiliations:** 1 Institute of Integrative Biology, ETH Zürich, 8092 Zürich, Switzerland; 2 Department of Biology, Stanford University, Stanford, CA 94305 USA; 3 Department of Science, Roma Tre University, Rome, Italy; 4 Natural Resources Canada, Canadian Forest Service, Great Lakes Forestry Centre, Sault Ste Marie, ON P6A 2E5 Canada; 5 Environmental Change Institute, School of Geography and the Environment, University of Oxford, Oxford, UK; 6 Lancaster Environment Centre, Lancaster University, Lancaster, UK; 7 Department of Aquatic Ecology & Environmental Biology, Institute for Water and Wetland Research, Radboud University, Nijmegen, The Netherlands; 8 Department of Geography, King’s College London, 30 Aldwych, London, WC2B 4BG UK; 9 Department of Biotechnologies and Life Sciences (DBSV), University of Insubria, 21100 Varese, Italy; 10 Goethe University, Institute for Physical Geography, Altenhöferallee 1, 60438 Frankfurt am Main, Germany; 11 Biology Department, Faculty of Natural Sciences, Universidad del Rosario, Avenida carrera 24 # 63C-69, Bogotá, Colombia; 12 Institute of Botany, University of Natural Resources and Life Sciences, Gregor Mendel St. 33, 1190 Vienna, Austria; 13 Environmental Research Institute, University of Waikato, Hamilton, New Zealand; 14 Research Center for Advanced Science and Technology, The University of Tokyo, 4-6-1 Komaba, Meguro, Tokyo, 153-8904 Japan; 15 Chair of Crop Science and Plant Biology, Estonian University of Life Sciences, Tartu, 51006 Estonia; 16 Department of Ecology, Universidade Federal do Rio Grande do Sul, Porto Alegre, RS 91501-970 Brazil; 17 AMAP, Univ Montpellier, INRAe, CIRAD, CNRS, IRD, Montpellier, France; 18 Departamento de Botânica, Universidade Federal de Pernambuco, Recife, Pernambuco Brazil; 19 Laboratorio Nacional de Ciencias de la Sostenibilidad, Instituto de Ecología, Universidad Nacional Autónoma de México, A.P. 70-275, Ciudad Universitaria, Coyoacán, 04510 Mexico City, Mexico; 20 Institute of Landscape and Plant Ecology, University of Hohenheim, Ottilie-Zeller-Weg 2, D-70599 Stuttgart, Germany; 21 Komarov Botanical Institute, Prof. Popov str., 2, St. Petersburg, 197376 Russia; 22 Department of Forestry, Santa Catarina State University, Lages, SC 88520-000 Brazil; 23 Embrapa Clima Temperado, Pelotas, RS 96010-971 Brazil; 24 Institute of Environmental Science, Leiden University, 2333 CC Leiden, the Netherlands; 25 Department of Biology, University of Wisconsin – Eau Claire, Eau Claire, WI 54702 USA; 26 Max Planck Institute for Biogeochemistry, 07745 Jena, Germany; 27 German Centre for Integrative Biodiversity Research (iDiv) Halle-Jena-Leipzig, Deutscher Platz 5e, 04103 Leipzig, Germany

**Keywords:** Ecophysiology, Biogeography, Ecology

## Abstract

Due to massive energetic investments in woody support structures, trees are subject to unique physiological, mechanical, and ecological pressures not experienced by herbaceous plants. Despite a wealth of studies exploring trait relationships across the entire plant kingdom, the dominant traits underpinning these unique aspects of tree form and function remain unclear. Here, by considering 18 functional traits, encompassing leaf, seed, bark, wood, crown, and root characteristics, we quantify the multidimensional relationships in tree trait expression. We find that nearly half of trait variation is captured by two axes: one reflecting leaf economics, the other reflecting tree size and competition for light. Yet these orthogonal axes reveal strong environmental convergence, exhibiting correlated responses to temperature, moisture, and elevation. By subsequently exploring multidimensional trait relationships, we show that the full dimensionality of trait space is captured by eight distinct clusters, each reflecting a unique aspect of tree form and function. Collectively, this work identifies a core set of traits needed to quantify global patterns in functional biodiversity, and it contributes to our fundamental understanding of the functioning of forests worldwide.

## Abstract (web summary)

Understanding patterns in woody plant trait relationships and trade-offs is challenging. Here, by applying machine learning and data imputation methods to a global database of georeferenced trait measurements, the authors unravel key relationships in tree functional traits at the global scale.

## Introduction

Trees play a fundamental role in forested ecosystems, driving carbon capture, nutrient cycling, and water dynamics, with forest ecosystems supporting enormous terrestrial biodiversity and being critically important for livelihoods worldwide^1^. The types of trees that can survive in a given location ultimately depend on their functional traits, which include all the physiological and morphological features that determine how they interact with, influence, and respond to their environment^2^. By governing trees’ water, nutrient, and light economies^2–6^, functional traits which elevate performance in one habitat typically reduce performance in others, leading to selection for specific traits across environments^7^. Genetic, morphological, and biophysical constraints subsequently limit the range of traits that a species can exhibit, leading to so-called ecological ‘trade-offs’ that shape species’ geographic distributions^8^, coexistence mechanisms^9^, and the provision of ecosystem services^1,10^. Identifying the key relationships that underpin global variation in tree trait expression is needed for quantifying the functional biodiversity of forests, and will be critical for predicting how forest diversity, composition, and function will respond to changing environmental conditions^11,12^.

Despite a wealth of research into trait relationships across the plant kingdom, our understanding of organismal-level trait coordination in trees lags behind that of herbaceous species^6,12,13^. Prior research has identified a key set of functional traits that summarize the spectrum of form and function across the plant kingdom, with leaf economics and plant size being the dominant trait axes underpinning life-history strategies^5,14–16^. Yet, because such studies have traditionally focused only on a small handful of traits estimated at the species level—often heavily biased towards leaf traits^17^—the full dimensionality of trait space remains unknown. This is particularly problematic for trees, which, due to their size, longevity, ontogeny, and unique structural properties, have distinct characteristics and face novel abiotic stressors relative to herbaceous plants^6,13,18–21^. Trait analyses which include both woody and herbaceous plants typically omit critical aspects of tree architecture (e.g. bark properties or crown size), as these traits are either altogether absent in herbaceous plants or because they are rarely measured on non-woody species. Thus, our current understanding of dominant trait patterns in plants fundamentally overlooks the massive energetic investments in structures that are unique to large woody species^21,22^.

A challenge when measuring and quantifying forest functional biodiversity is the enormous range of putative traits that can be measured, in tandem with the relative difficulty of measuring many tree traits relative to herbaceous plants (e.g. root depth)^2^. As with all plants, the functional biogeography of trees is known to be partly governed by leaf economics^13^, which are critical for light acquisition and water balance, as well as wood and root traits, which are fundamental for structural stability and water transport and nutrient acquisition^6,23^. Traits such as seed characteristics and crown dimensions can be critical drivers of successional trajectories, canopy positioning, and life-history strategies across the landscape^24–26^. Identifying the multidimensional trait relationships that integrate these different tree parts is urgently needed to create standardized, systematic metrics of forest functional biodiversity across the globe^12,27,28^.

A key limitation when exploring functional trait relationships is the sparsity inherent in most plant trait databases^5,17,29,30^. Such restrictions limit traditional analyses to a small subset of traits and species where there is complete coverage, often capturing only a tiny fraction of known species (e.g. <1% of all plants^5^). To overcome this limitation, studies increasingly use imputation approaches to estimate species-level trait averages using phylogenetic and taxonomic information^17,30,31^. Yet, because these approaches focus solely on species-level averages (i.e. “gap-filling” of the species-by-trait matrix^30^), they are inherently biased towards traits which are highly phylogenetically conserved and which exhibit minimal environmental plasticity or intraspecific variation^29^. To the extent that environmentally sensitive traits are even included, their role in individual-level trait relationships can be underestimated due to these species-level trait averages having little ecological relevance^32^. To explore relationships between phylogenetically conserved traits and environmentally plastic traits, trait-imputation methods must consider evolutionary history along with the local environmental conditions^32^. Given the enormous ontogenetic and environmental variation observed in many important tree traits—including stem size, crown size, leaf geometry, tree height, and root depth^23–25^—incorporating both phylogenetic information and environmental information is particularly important when exploring trait patterns in trees.

**Fig. 1:**
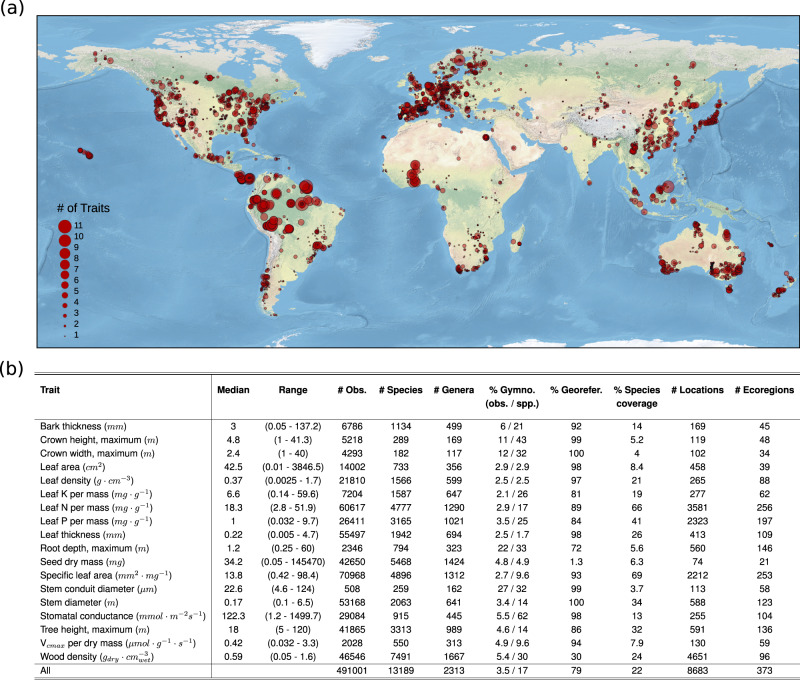
Overview of the 18 functional traits. **a** The unique geographic locations (*n* = 8683) where tree functional traits were recorded. The size of the circles denotes the relative number of unique traits (out of 18 possible) that were measured at each location, regardless of species identity. **b** Summary statistics for the 18 traits considered here (see Supplementary Table 1–3, Supplementary Figs. 1, 2 for additional information). The analysis included 491,001 trait measurements, encompassing 13,189 unique tree species and 2313 unique genera.

Here, we use a global trait database^29^ comprising nearly 500,000 trait measurements across more than 13,000 tree species to explore relationships among 18 functional traits, reflecting leaf economics, wood structure, bark thickness, tree size and crown size, seed size, and root depth (Fig. 1b). To overcome the data sparsity and focus on relationships across all 18 traits, we develop a series of non-parametric machine-learning models that provide spatially-explicit estimates of trait expression of an individual tree in a given location, as a function of its evolutionary history and the local environmental conditions. Using the resulting dataset, we ask: (1) Which traits and environmental variables best capture overall variation in tree trait expression? and (2) What is the dimensionality of trait space and the dominant multi-trait clusters that capture the full breadth of tree form and function at the global scale?

Our expectation was that traits underpinning the leaf-economic spectrum would continue to emerge as a key driver of trait relationships, primarily reflecting the broad differences in leaf morphology and physiology between angiosperms and gymnosperms^5^. Yet, due to the wide range of traits being considered here, we expected the dimensionality of the trait space to be more complex than when considering a small set of traits, or when omitting traits integral to tree functioning (e.g. wood, crown, or bark traits). Indeed, our analysis shows that the dominant trait axes underpinning trait variation in trees closely mirror those found across the plant kingdom^5^, with the first axis representing leaf-level resource economies, and the second axis capturing whole-tree size and light competition. Yet these two axes account for less than half of overall trait variation in trees, suggesting more complex trait associations governing the full variation in life-history strategies. By subsequently exploring multidimensional relationships across all traits, we identify a unique set of eight functional clusters that reflects the full breadth of tree form and function, and which can aid in trait selection for global studies of forest functional biodiversity. Collectively, this work identifies emergent constraints on tree functional biogeography, and sheds light on the core set of traits needed to quantify and study forest functional diversity worldwide.

## Results

### Trait models

Our analysis included 491,001 unique trait measurements across 18 traits, encompassing 13,189 tree species from 2313 genera, reflecting ~21% of all known tree species^33^ (Fig. 1). Traits were measured at 8683 locations across the globe and 373 distinct eco-regions (Supplementary Tables 1, 2), with georeferenced measurements capturing 15% of known tree species in Eurasia, 13% in South America, 9% in Oceania, and 6% in North America and Africa^33^. The raw data covered 22% of all trait-by-species combinations (Fig. 1b, Supplementary Fig. 2), nearly identical to other large-scale trait analyses across the entire plant kingdom^5,17,30^. Yet there was considerable variation in coverage across traits, with traits such as specific leaf area and leaf nitrogen measured on more than 60% of all species, versus traits such as crown diameter and conduit diameter, which captured fewer than 5% of species (Fig. 1b, Supplementary Fig. 2). Across all species, 423 had more than 10 unique traits measured, and two species (*Picea abies* and *Pinus sylvestris*) had measurements for all 18 traits. In general, there was highly consistent coverage across taxonomic orders and traits (Supplementary Fig. 1), with gymnosperms being slightly overrepresented (comprising 3.1 ± 6.8% of measurements in the database versus ~1% of all known tree species^34,35^, Fig. 1a), in part reflecting the wider geographic range of many gymnosperms relative to angiosperms^36^.

To explore relationships in functional traits at the individual level, we used random-forest machine-learning models to estimate missing trait values for each individual tree as a function of its environment and phylogenetic history. We also conducted a second set of analyses where trait expression was estimated using phylogenetic information only, which allowed us to include additional non-georeferenced data (Fig. 1), while also quantifying the relative contribution of environmental information on trait expression (Supplementary Fig. 6). Following standard approaches^5,15,29,30^, all traits were log-transformed and standardized to allow for statistically robust comparisons. Environmental predictors included ten variables encompassing climate^37–40^, soil^41^, topographic^42^, and geological^43^ features. Phylogenetic history was incorporated via the first ten phylogenetic eigenvectors^44,45^ (see Methods). By including environmental information alongside phylogenetic information, this approach not only allowed us to impute species-level traits which have strong phylogenetic signals and weak environmental signals, as is traditionally done^17,30^ but also to robustly estimate traits which have a weak phylogenetic signal and are instead strongly sensitive to environmental conditions. Moreover, being a non-parametric approach, the random forest makes no a priori assumptions about how trait expression varies across phylogenetic groups or environments.

Across all 18 traits, the best-fitting models explained 54 ± 14% of out-of-fit trait variation (VEcv, see Methods), ranging from 26% for stem diameter to 76% of the variation in leaf area (Supplementary Figs. 6, 7). This accuracy was quantified using buffered leave-one-out cross-validation to account for spatial and phylogenetic autocorrelation^46^, and thus serves as a conservative lower bound for species which are phylogenetically and environmentally distinct from the observations^47^. There was no significant relationship between out-of-fit cross-validation accuracy and sample size (R^2^ = 0.06, *p* = 0.33), highlighting the relatively broad taxonomic coverage for each trait (Fig. 1, Supplementary Fig. 1).

Environmental variables and phylogenetic information had approximately equal explanatory power (relative importance of 0.51 vs 0.49 for environment vs. phylogeny), albeit with substantial variation across traits (Supplementary Fig. 9). The inclusion of environmental variables increased the explanatory power of the models by 35%, on average (Supplementary Fig. 6), with crown diameter, crown height, leaf density, and stem diameter exhibiting the largest relative increases (54%, 45%, 73%, and 26%, respectively), mirroring the fact that these traits have comparatively low phylogenetic signal relative to other traits (assessed via Pagel’s *λ* on the raw data, Fig. 4c). Seed dry mass was the only trait with a substantial increase in accuracy using the phylogeny-only model (25% improvement; Supplementary Fig. 6), reflecting the fact that seed dry mass had the strongest phylogenetic signal of all traits (Fig. 4c), and also because this trait has a substantial amount of additional non-georeferenced data that was included in the phylogeny-only models (Fig. 1b). Wood density was the only trait with nearly identical predictive power whether or not environmental information was included, whereas all other traits exhibited significantly reduced accuracy when environmental information was excluded (Supplementary Fig. 6).

### Relationships in tree trait expression

Using the resulting trait models, we imputed missing trait values for every tree with at least one georeferenced trait measurement. For all traits except seed dry mass, we used the random-forest models accounting for environmental and phylogenetic information; for seed dry mass, we used the phylogeny-only model to estimate expression due to its substantially higher data availability and out-of-fit accuracy. For tree height, stem diameter, crown height, crown width, and root depth, we used quantile random forest^48^ to estimate the upper 90th percentile value for each species in its given location, thereby minimizing ontogenetic variation across a tree’s lifetime (see Methods). We used the resulting trait data to explore the dominant drivers of trait variation using species-weighted principal component analysis, accounting for an unequal number of observations across species.

**Fig. 2:**
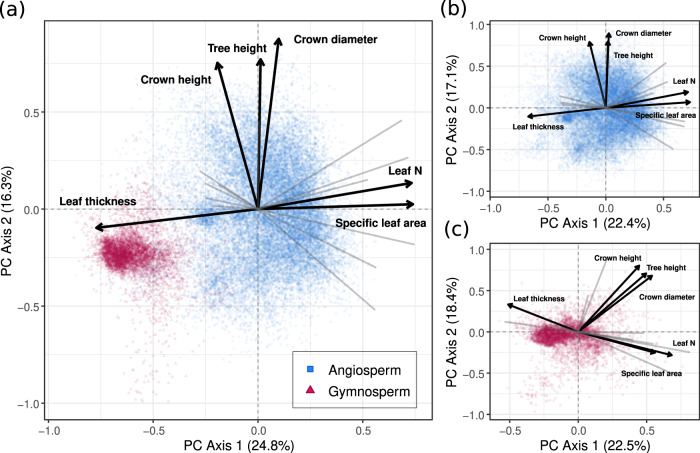
The dominant trait axes and relationships. Shown are the first two principal component axes capturing trait relationships across the 18 functional traits. **a** All tree species (*n* = 30,146 observations), **b** angiosperms only (*n* = 24,658), and **c** gymnosperms only (*n* = 5498). In **a** the three variables that load most strongly on each axis are shown in dark black lines, with the remaining variables shown in light grey. These same six variables are highlighted in **b** and **c** illustrating how the same relationships extend to angiosperms and gymnosperms (see Supplementary Figs. 10–12 for the full PCAs with all traits visible, and Supplementary Table 5 for the PC loadings).

When considering all traits simultaneously, the first two axes of the resulting principal components (PC) capture 41% of the variation in overall trait expression (Fig. 2a; Supplementary Fig. 10; Supplementary Table 5). The first trait axis correlates most strongly with leaf thickness, specific leaf area, and leaf nitrogen (PC loadings of L = 0.77, 0.74, and 0.73, respectively). By capturing key aspects of the leaf-economic spectrum^14^, these traits reflect various physiological controls on leaf-level resource processing, tissue turnover and photosynthetic rates^49^. Thick leaves with low specific leaf area (SLA) can help minimize desiccation, frost damage, and nutrient limitation, but at the cost of reduced photosynthetic potential due to primary investment in structural resistance^50^. Accordingly, leaf nitrogen—a crucial component of Rubisco for photosynthesis^51^—trades off strongly with leaf thickness. This first axis thus captures the core distinction between “acquisitive” (fast) and “conservative” (slow) life-history strategies across the plant kingdom^7,52^, reflecting an organismal-level trade-off between the high photosynthetic potential in optimal conditions versus abiotic tolerance in suboptimal conditions. Nevertheless, leaf density—which is related to SLA and is a key feature of the leaf-economic spectrum—loads relatively weakly on this first trait axis compared to other leaf traits (L = −0.28 for axis 1, vs 0.20 for axis 2; Supplementary Table 5), highlighting important aspects of leaf structure that are not captured by this dominant trait axis^53^.

The second trait axis correlates most strongly with maximum tree height (PC loading of L = 0.77), crown height, (L = 0.75), and crown diameter (L = 0.88), highlighting the overarching importance of competition for light and canopy position in forests^7^ (Fig. 2a; Supplementary Fig. 10; Supplementary Table 5). Large trees and large crowns are critical for light access and for maximizing light interception down through the canopy^54^. Nevertheless, tall trees with deep crowns also experience greater susceptibility to disturbance and mechanical damage, primarily due to wind and weight^25^. Because of the massive carbon and nutrient costs required to create large woody structures^55,56^, larger trees are less viable in nutrient-limited or colder climates^57^, and in exposed areas with high winds or extreme weather events^58^. This second axis thus reflects a fundamental biotic/abiotic trade-off related to overall tree size, which is largely orthogonal to leaf-level nutrient-use and photosynthetic capacity.

Despite substantial differences in wood and leaf structures between angiosperms and gymnosperms (e.g. vessels vs. tracheids), the two main relationships hold within, as well as across, angiosperms and gymnosperms (Fig. 2b, c; Supplementary Figs. 11, 12). Indeed, angiosperms and gymnosperms are subject to the same physical, mechanical, and chemical processes that determine the ability to withstand various biotic and abiotic pressures^59^.

Collectively, these two primary trait axes capture two dominant ecological trade-offs that underpin tree survival in any given environment: (1) the ability to maximize leaf photosynthetic activity, at the cost of increased risk of leaf desiccation, and (2) the ability to compete for space and maximize light interception, at the cost of increased susceptibility to mechanical damage. By capturing two aspects of conservative-acquisitive life-history strategies, these two relationships closely mirror those seen when considering herbaceous species alongside woody species^5,17^. However, in line with our expectations, these two axes capture only ~40% of the variation in trait space, versus nearly ~75% of variation when considering only six traits across the entire plant kingdom^5^. Here, the first seven PC axes are needed to account for 75% of the variation across all 18 traits (Supplementary Table 5). Thus, while this analysis supports the universality of these two primary PC axes, it also demonstrates that the majority of trait variation in trees is unexplained by these two dimensions. As such, quantifying the full dimensionality of trait space by exploring multidimensional trait clusters is needed to better capture the wide breadth of tree form and function.

### Environmental predictors of trait relationships

To examine how environmental variation shapes trait expression across the globe, we next quantified the relationships between environmental conditions and the dominant trait axes. Using Shapley values^60^, we partitioned the relative influence of each environmental variable on the PC trait axes, controlling for all other variables in the model (see Methods).

**Fig. 3:**
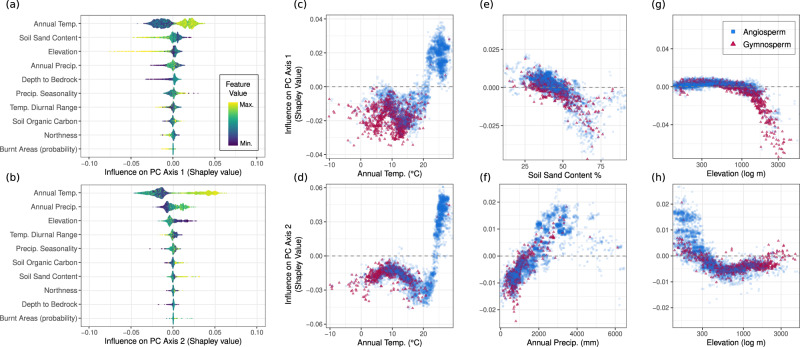
The relationship between environmental variables and trait axes. **a**, **b** The relative influence of the environmental variables on the two dominant PC axes. The ten variables are sorted by overall variable importance in the models (see Methods). Yellow points are observations which have high values of that environmental variable; blue values are the lowest. Points to the right of zero indicate a positive influence on the PC axis; points to the left indicate a negative influence (see also Supplementary Figs. 17, 18). **c**–**h** The relationships between environmental variables and PC axis values for the three variables in **a** with the strongest influence. Values above zero show a positive influence on PC axis values; values less than zero indicate a negative influence.

In line with previous analysis across the plant kingdom^61^, temperature variables were the strongest drivers of trait relationships (Fig. 3, Supplementary Figs. 17, 18), with annual temperature having the strongest influence both on leaf-economic traits (PC axis 1, Fig. 3c) and on tree-size traits (PC axis 2, Fig. 3d). Leaves face increased frost risk and reduced photosynthetic potential in colder conditions, such that ecological selection should favour thick leaves with low SLA over thin leaves with high SLA and high nutrient-use^49^. Trees in warm environments are more likely to experience strong biotic interactions, which should increase evolutionary and ecological selection pressures over time^62,63^, favouring tall species with large crowns that have high competitive ability and efficient light acquisition strategies. Annual temperature thus predominantly reflects the transition from gymnosperm- to angiosperm-dominated ecosystems, with this inflection point occurring at ~15 °C for both axes, demonstrating strong environmental convergence between the dominant axes of trait variation.

Beyond annual temperature, each trait axis demonstrated different relationships with climate, soil, and topographic variables (Fig. 3a, b, Supplementary Figs. 17, 18). Percent sand content had the second-highest influence on the first trait axis (Fig. 3e), supporting patterns seen across the entire plant kingdom^17^. Sand content is a strong proxy for soil moisture and soil-available nutrients such as phosphorous, and is therefore closely tied to leaf photosynthetic rates^64^. In contrast to previous work, however, we find that soil characteristics have correspondingly little effect on the second axis of trait variation (Fig. 3b; Supplementary Fig. 18). Instead, precipitation was the second strongest driver of tree height and crown size (Fig. 3f), with large trees with large crowns becoming consistently more frequent with increasing precipitation. These results highlight that, despite the primary importance of temperature, the main climate stressors to trees (e.g. xylem cavitation and embolism, fire regimes, and leaf desiccation) typically arise via interactions between temperature, soil nutrients, and water availability.

For both axes, elevation was the third strongest driver of trait values (Fig. 3g, h), highlighting a critical component of tree functional biogeography that extends beyond climate and soil. Yet the effects of elevation on trait expression differed somewhat across the two axes. For the first axis related to leaf-economic traits, there is little influence at low elevations, followed by a sharp transition at ~2000 m towards gymnosperm-dominated species with thick leaves, low SLA, and low leaf N. For the second trait axis related to tree size, elevation instead has a strong positive influence on tree height and crown size at low elevations, which becomes increasingly less influential past ~500 m. Such results partly reflect the transition from angiosperm to gymnosperm-dominated stands at higher elevations (blue vs. red points, Fig. 3g, h), and potentially the role of environmentally mediated intraspecific variation in traits such as tree height^65,66^.

These results demonstrate close alignment of the dominant trait PC axes across biogeographic regions. Despite the orthogonality of these axes in trait species, environmental conditions place similar constraints on both trait axes, particularly at the environmental extremes (e.g. warm, moist, low elevation vs. cold, dry, high elevation), leading to convergence of the dominant trait axes across environmental gradients.

### Trait clusters at the global scale

To better explore the multidimensional nature of trait relationships that are not fully covered by the dominant two axes, we subsequently identified groups of traits that form tightly coupled clusters and which reflect distinct aspects of tree form and function.

**Fig. 4:**
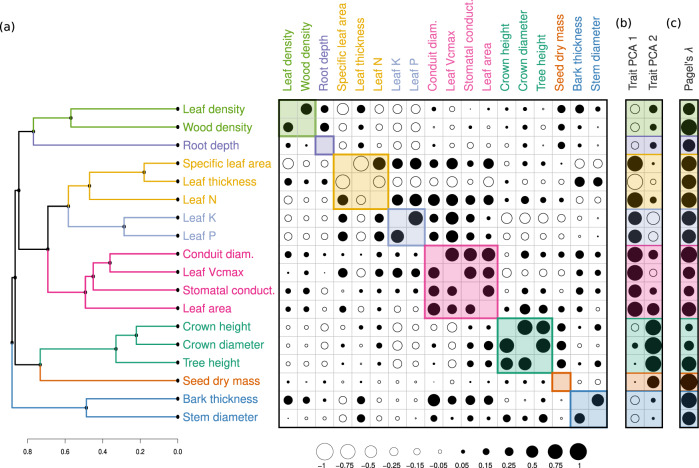
Trait correlations and functional clusters. **a** Trait clusters with high average intra-group correlation. The upper triangle gives the species-weighted correlations incorporating intraspecific variation. The lower triangle gives the corresponding correlations among phylogenetic independent contrasts, which adjusts for pseudo-replication due to the non-independence of closely related species. The size of the circle denotes the relative strength of the correlation, with solid circles denoting positive correlations and open circles denoting negative correlations (see Supplementary Fig. 19 for the numeric values). **b** PC loadings for each trait and each of the first two principal component axes, illustrating which functional trait clusters align most strongly with the dominant axes of trait variation (see Supplementary Table 5 for the full set of PC loadings). **c** The species-level phylogenetic signal of each trait (Pagel’s λ), calculated using only the raw trait values.

Our results show that these 18 traits can be grouped into eight trait clusters, each of which reflects a unique aspect of morphology, physiology, or ecology (Fig. 4a, Supplementary Fig. 23). The largest trait cluster (Fig. 4a, pink cluster) demonstrates wood/leaf integration of moisture regulation and photosynthetic activity via the inclusion of leaf area, stem conduit diameter, stomatal conductance, and leaf V_cmax_ (the maximum rate of carboxylation). Distinct from this cluster are the three traits loading most strongly on PC axis 1 (SLA, leaf thickness and leaf N; Fig. 4a, yellow), highlighting complementary aspects of the leaf-economic spectrum indicative of acquisitive vs. conservative resource use^15^. The role of leaf K and P in leaf nutrient economies are well established^7,67^, and yet these traits form a distinct cluster from the other leaf-economic traits (Fig. 4a, light blue) due to their relatively high correlation with tree height and crown size, particularly for leaf K, which loads almost equally on both trait axes (Fig. 4b, Supplementary Table 5).

Tree height and crown size form their own distinct cluster (Fig. 4a, dark green), further supporting the inference that these traits reflect key aspects of tree form and function independent of the leaf-economic spectrum. Yet leaf area, despite being part of the cluster reflecting moisture regulation and photosynthetic activity, loads almost equally on PC axes 1 and 2 (Fig. 4b, Supplementary Table 5), highlighting that it serves as an intermediary between the two key aspects of tree size and leaf economics. It is a critical driver of moisture regulation and photosynthetic capacity, while also playing an important role in the light acquisition, leaf-turnover time, and competitive ability^54,68^.

There are two additional two-trait clusters, both of which load relatively poorly on the two primary PC axes: (1) stem diameter and bark thickness (Fig. 4, dark blue), and (2) wood and leaf density (Fig. 4, light green). Bark thickness increases with tree size not only as a result of bark accumulation as trees age, but also due to the functional/metabolic needs of the plant^69,70^. From an ecological perspective, thick bark can be critical for defense against fire and pest damage (mainly a thick outer bark region), for storage and photosynthate transportation needs (mainly a thick inner bark region)^71,72^. Yet such relationships are strongly ecosystem-dependent, with tree size emerging as the dominant driver at the global scale^70^. In contrast, wood density and leaf density are strongly linked to slow/fast life-history strategies, where denser plant parts reduce growth rate and water transport^6,15^ but protect against pest damage, desiccation, and mechanical breakage^6,50,56^. As such, leaf density captures fundamentally unique aspects of leaf form and function relative to other leaf traits such as SLA^53^ (Fig. 4b, Supplementary Table 5), and our results support the inference that these translate into fundamentally different ecological strategies^73^. Collectively, these two-trait clusters each demonstrate unique and complementary mechanisms that insulate trees against various disturbances and extreme weather events, but at the cost of reduced growth, competitive ability, and productivity under optimal conditions (see Supplementary Notes).

Lastly, two traits each comprise their own unique cluster: root depth and seed dry mass (Fig. 4a, purple and orange, respectively). Root growth is subject to a range of belowground processes (e.g. root herbivory, depth to bedrock), and our results confirm previous work demonstrating a clear disconnect between aboveground and belowground traits^23,74,75^. Root depth accordingly has a relatively weak phylogenetic signal (*λ* = 0.44, Fig. 4c) but a strong environmental signal (Supplementary Figs. 6, 9), reflecting distinct belowground constraints on trait expression^23^. In contrast, seed dry mass exhibits the strongest phylogenetic signal (*λ* = 0.98, Fig. 4c) and weakest environmental signal of any trait (Supplementary Figs. 6, 9), and it accordingly was the only trait where the phylogeny-only model performed substantially better (Supplementary Fig. 6). In line with previous work, seed dry mass has moderate correlations with various other traits underpinning leaf economics and tree size^5,28^ (e.g. ρ = 0.28, −0.22, and 0.22 for tree height, leaf K, and leaf density, using the raw data), yet it exhibits relatively weak correlation with most other traits, placing it in a distinct functional cluster. Reproductive traits are subject to unique evolutionary pressures^26^, indicative of different seed dispersal vectors (wind, water, animals) and various ecological stressors that uniquely affect seed viability and germination^26^. The emergence of root depth and seed dry mass as solo functional clusters thus supports the previous inference that belowground traits^74^ and reproductive traits^26^ reflect distinct aspects of tree form and function not fully captured by leaf or wood trait spectrums.

## Discussion

This work provides a baseline understanding of tree form and function, aimed at informing future research into the functional biodiversity of forests. Our lack of understanding of the trait relationships unique to woody species is partly due to the enormous number of putative traits that can be measured on trees^7,29^. To help address these challenges, the eight distinct trait clusters identified here (Fig. 4) can help inform future research into forest functional biodiversity. First, this work indicates traits which are largely redundant at the global scale (e.g. SLA and leaf N), versus those that occupy clear distinct roles (seed dry mass and root depth). Second, by quantifying the relative importance of environmental conditions vs. phylogenetic history as drivers of trait expression (Fig. 4c, Supplementary Figs. 6, 9), these results can aid in the selection of representative traits from each cluster, depending on the intended question or application (e.g. by selecting environmentally sensitive traits such as crown size, vs. more phylogenetically conserved traits such as maximum tree height). Such insight can help with the adoption of a standardized set of tree traits that allow for consistent quantification and comparison of forest biodiversity on a global scale.

The 18 physiological and morphological traits used here were selected in part due to their representation in prior trait analyses, their functional uniqueness, their relevance to tree structure and architecture, and the data quality. Thus, as with all functional trait analyses, there are additional traits and metrics not considered here which may capture complementary aspects of tree form and function. For example, various allometric relationships (e.g. the ratio of tree height to stem diameter) may better capture energetic allocation in trees than do physiological traits^76^ by reflecting unique aspects of tree morphology (see Supplementary Figs. 24, 25 for analysis exploring allometric ratios). Alternatively, belowground traits such as root architecture and chemistry remain particularly underrepresented for trees, despite capturing potentially unique aspects of tree form and function^74,75^. Increasing the representation of such traits in trait databases is an important next step, and critical for quantifying the multi-functionality of forest biodiversity.

Here, by focusing on trees, we have significantly higher species-level representation compared to kingdom-wide analyses, encompassing more than 20% of known tree species and 22% of all trait-by-species combinations (Supplementary Fig. 2), comparable to other large-scale analyses exploring trait relationships across the entire plant kingdom^5,17,30^. However, because we explore patterns obtained using imputed data—which can introduce issues of circularity and imputation bias—we conducted a series of sensitivity analyses to test the robustness of our findings to various modelling assumptions. Most importantly, when using the raw (un-imputed) data to estimate pairwise correlations, we see that the PCA results, functional clusters, and environmental relationships are nearly identical to those obtained using the imputed data (Supplementary Fig. 20–23). Additionally, our results are likewise insensitive to the selection of phylogeny-only vs. phylogeny + environment models (Supplementary Fig. 13), to the use of all 52,255 tree species in the reference phylogeny (Supplementary Fig. 14), to various levels of missingness in the data (Supplementary Fig. 15), and to the number of predictor variables in the models (Supplementary Fig. 16). Indeed, the value of the PCA and clustering approaches used here is that they rely only on accurate estimates of pairwise correlations, which have shown to be highly robust to data sparsity^5,30^. Moreover, by focusing only on correlative analyses, we can directly validate these results using the raw data (Supplementary Figs. 20–23).

Nevertheless, an ongoing challenge in plant trait analyses is to improve the taxonomic and spatial representation of trait measurements^29^. We had generally broad spatial coverage (Supplementary Tables 1, 2; Fig. 1), with notable exceptions being the interior of Africa and northeast Asia, where trait data is lacking in general^29^. Similarly, we had largely consistent representation across taxonomic orders (Fig. 1, Supplementary Fig. 1), albeit with variation across traits and orders, with species in the order Pinales, for example, being consistently overrepresented (comprising 4% of observations vs ~1% of known tree species; Supplementary Fig. 1), versus those in the orders Asterales and Solanales being slightly underrepresented (1% of observations vs ~2% of known tree species). By using buffered leave-one-out cross-validation^46^, our accuracy estimates present a conservative lower bound for such species^47^ (Supplementary Fig. 6). Nonetheless, caution should still be exercised when using trait-imputation approaches to make inferences of trait values for a specific tree in a given location, particularly on the unlogged scale. Nearly all traits exhibit skewed log-normal distributions (where the sample variance is proportional to the mean), and so traits must be log-transformed to allow for statistically valid comparisons^5,15,29,30^. As such, estimates on the raw (linear) scale can be subject to unavoidably high variation, regardless of model accuracy (here, 29 ± 19% median relative absolute error on the unlogged scale, ranging from 12% for wood density to 86% for seed dry mass). Thus, although our approach yields robust insights into global patterns that hold when making comparative analyses of aggregate trends across species^5,17^ (Supplementary Fig. 8), obtaining highly precise estimates of trait expression for a given tree in a given location remains a fundamental challenge in functional ecology.

Our analysis differs from previous approaches^5,17,30^ in that we use non-parametric machine-learning models to estimate trait expression as a function of phylogenetic history and environmental variables. This allows us to model traits regardless of their degree of phylogenetic signal or relationships to abiotic conditions (Fig. 4c, Supplementary Figs. 6, 9). Yet there are likely additional abiotic and biotic factors not included here which are important drivers of trait expression. For example, forest age and successional stage are key drivers of tree morphology and stand structure^77^; and forest management practices, disturbance history, human activity, and native/introduced status can all play important roles in tree trait expression^78^ (see Supplementary Notes). Local environmental conditions (e.g. sun vs. shade, microsite soil characteristics) are also critical drivers of individual tree morphology and physiology, and landscape-level climate variables like those used here may underestimate the extent of these local abiotic controls^79^. By including environmental variation alongside phylogenetic information, our approach should better incorporate these non-taxonomic drivers of trait expression than approaches which do not allow for intraspecific variation. Yet including high-resolution microsite data and stand-level information in existing trait databases will be critical for gaining a more nuanced understanding of the drivers of tree trait expression. Here, by quantifying the full dimensionality of trait space, this work can serve as a baseline for such research, helping to identify the dominant traits that underpin the functional biodiversity of forests.

Collectively, this work reveals key relationships and trait clusters governing tree form and function worldwide. We show that tree functional traits predominantly reflect two major functional axes: one representing leaf-level photosynthetic capacity and resource economies, and the other representing competition for light via tree and crown size. Mirroring patterns seen across the entire plant kingdom, these patterns capture an ecological gradient from conservative strategies under suboptimal environments (cold, dry, high elevation), to acquisitive strategies associated with light competition in high-resource environments (warm, high soil quality, low elevation). However, these two axes capture less than half of the overall variation in tree form and function. By subsequently exploring multidimensional relationships across all traits, we identify a unique set of eight functional clusters that reflects the full breadth of tree form and function. In doing so, these results elucidate key constraints on functional trait relationships in trees, contributing to our fundamental understanding of the controls on the function, distribution, and composition of forest communities. By identifying a core set of traits that reflect the broad variety of ecological life-history strategies in trees, this work can inform future trait-based research into the functional biodiversity of the global forest system.

## Methods

### Trait information

Trait data were obtained from the TRY plant trait database^29^ v. 5.0. Data were cleaned by converting all traits to standardized units and by matching species names to The Plant List (TPL) database v1.1 (http://www.theplantlist.org, accessed June 2020) using the *Taxonstand* package in R v3.6.0^80^. Synonyms were replaced with accepted names, when available. The phylogenetic tree was taken from the seed plant phylogeny of Smith & Brown^81^, and species names were likewise cleaned and harmonized using the TPL database. To limit our analysis to tree functional traits only, we used the BGCI GlobalTreeSearch database v1.3^35,82^, containing a comprehensive list of ca. 60,000 tree species compiled and harmonized from across 500 sources. The BGCI database uses TPL for much of its taxonomic identification, but to ensure consistency among all sources we used the same name harmonization pipeline as with TRY and the seed plant phylogeny. We constrained the set of traits and the phylogenetic tree to those species that could be matched to the BGCI database (*n* = 52,255 species matched, excluding monocots), and we likewise trimmed the phylogenetic tree to the set of species matched in TRY

For comparison with previous work on trait trade-offs, we focused on physiological and morphological traits directly measured at the individual level, rather than derived traits such as those related to tree allometry (although see Supplementary Figs. 24, 25 and Supplementary Notes). The core set of 18 traits was selected from the TRY database first based on their use in prior global analyses^5^ (leaf area, specific leaf area, seed dry mass, maximum tree height, leaf nitrogen, wood density), their importance in the leaf economics spectrum^14,15,20,83^ (leaf thickness, density, Vcmax, phosphorous, potassium, and stomatal conductance), their role in tree water transport and access^23,84^ (root depth, stem conduit diameter), and finally, those that are integral components of tree structure^6^ (stem diameter, bark thickness, crown height and diameter). This resulted in a primary set of 18 traits for use in the main analysis (Supplementary Table 3, Supplementary Data 1). For each trait, we selected sub-categories (as specified by TRY) that denoted comparable measurements and reflected uniform assay conditions (e.g. V_cmax_ measured at 25 °C) (Supplementary Table 3). We also obtained an additional 12 traits from the TRY to improve our ability to predict missing trait values via incorporating trait covariation (see model details, below). However, these were not used in the main analysis because they are either auto-correlated with one of the focal traits by definition (e.g. leaf width, leaf length, leaf C/N, leaf N/P, leaf moisture), they encompassed fewer than 150 species (root/shoot ratio, root N per mass, root C/N, and wood N per mass), or because they can vary substantially across measurement protocols or assay conditions but had insufficient metadata (i.e. lack of incubation temperature) needed for standardization (leaf J_max_, leaf respiration rate, leaf chlorophyll). Trait values were converted to common units where necessary (e.g. mm to cm), log-transformed, and normalized to have a mean of zero and a standard deviation of one, following standard approaches^5,15,29,30^.

### Trait-imputation models

In order to consider multidimensional trait relationships across all species and all traits, we used a machine-learning model to estimate the missing trait values for each species in each location. Specifically, we used random-forest (RF) models to estimate trait values for each georeferenced species using the *ranger* package in R^85^, with trait expression modelled as a function of each tree’s environment and phylogenetic history. The random-forest algorithm is determined by a specified set of “hyper-parameters”, which govern the splitting rules, variable selection, and stopping rules when building each tree in the forest^86,87^. Preliminary investigation showed that a wide variation in these hyper-parameters (e.g. using 1 versus 25 minimum observations per node) led to negligible improvements in model accuracy relative to the default parameters (i.e. <2% increase in out-of-bag R^2^). Thus, to minimize the risk of overfitting due to a large number of traits and models being considered here, we used the default *ranger* hyper-parameters for all traits (500 trees per forest; sampling with replacement; the number of variables per split equal to the square root of the number of predictors; a minimum of 5 observations per node; and the split rule determined by maximal variance). In order to minimize the influence of data-recording errors or unit mismatches in the dataset, trait values which occurred outside of the bulk of the trait distribution were investigated as outliers. Those which could not be externally verified and which were biologically unreasonable were removed (e.g. stem diameters > 15 m). When modeling tree height, crown size, and root depth, we only considered observations with height > 5 m, stem diameter > 10 cm, root depth > 25 cm, and crown height and width > 1 m^88^, thereby ensuring that our analysis focused on adult trees rather than saplings or woody shrubs. We subsequently implemented quantile random forest^48,89^ to estimate the upper 90th percentile trait value for maximum stem diameter, crown height and width, and root depth. In all other cases the imputed traits represent the mean predicted value across the random forest.

Environmental covariates considered for use in the models included 50 variables encompassing a range of climate^37–39^, soil^41^, topographic^42^, and geological^43^ variables (Supplementary Table 4). We omitted variables that directly measure plant community composition or biotic factors (e.g. NDVI or % forest cover) so as to ensure the resulting geographic layers solely encompassed non-biotic factors. Layers were sampled from a previously prepared global composite^90^. Briefly, all covariate map layers were resampled and reprojected to a unified pixel grid in EPSG:4326 (WGS84) at 30 arcsec resolution (~1 km^2^ at the equator). Layers with a higher original pixel resolution were downsampled using a mean aggregation method; layers with a lower original resolution were resampled using simple upsampling (that is, without interpolation) to align with the higher resolution grid. The set of environmental variables for each trait measurement was obtained by sampling this composite image at each unique latitude and longitude value given in the TRY database.

Phylogenetic information was incorporated in the form of phylogenetic eigenvectors^31,32,44,45^. We first calculated the pairwise cophenetic phylogenetic distance matrix across all 54,153 tree species that could be matched to both the BGCI tree list and the plant phylogeny. This matrix was then double-centred by rows and columns^44,91^, and the eigenvectors were sorted by percent variation explained across the phylogeny, with the first eigenvector accounting for the majority of variation. The first 50 orthogonal eigenvectors were extracted from this matrix for consideration as continuous predictors in the random-forest models.

To improve model parsimony and minimize overfitting, we used a sequential variable selection approach, whereby we selected *k* = 3, 5, 10, 15, 25, 50 phylogenetic and environmental variables (each), and fit the full set of models. The *k* phylogenetic eigenvectors for each step were selected by taking the first 1,...,*k* eigenvectors, sorted based on percent variation explained across the phylogeny. The *k* environmental variables were selected via clustering the full set of 50 variables into *k* groups and selecting a representative variable from each group, thereby minimizing correlation. Doing so revealed that out-of-fit model accuracy saturates at approximately *k* = 10 variables (Supplementary Fig. 5). We therefore used 10 phylogenetic eigenvectors and 10 environmental variables in the final models to improve model parsimony and minimize overfitting; however, the results are unchanged regardless of the number of covariates (Supplementary Fig. 6).

To leverage trait covariation among the disparate observations, we used a two-step algorithm to improve predictive power and imputation accuracy^31,92^. Using the general approach of Stekhoven & Bühlmann (2012), we first implemented a random forest on all traits for all observations. We then used these initial models to predict the full set of trait values for each observation (including the 12 ancillary traits not included in the focal analysis, Supplementary Table 3). We then refit the random-forest models for each trait, using the full set of predicted traits (apart from the focal traits) as covariates. For the final analysis, observed traits were used in place of imputed traits, when available, with the exception of maximum tree height, stem diameter, root depth, and crown size, where the upper 90th percentile trait values were used. Variable importance in the random-forest models (Supplementary Fig. 9) was calculated using the “permutation” metric, reflecting the variance in responses across predictors^85^.

By incorporating using both phylogenetic and environmental variables in the random-forest models, our approach makes no assumptions about which traits are more strongly governed by phylogeny versus environmental conditions. It thus provides a non-parametric alternative to taxonomic-based imputation methods^30^, allowing for traits with high phylogenetic signal and low environmental relationship, as well as those with low phylogenetic signal and high environmental relationship. Nevertheless, because some traits had substantial amounts of non-georeferenced information available, we conducted a second set of models using only the phylogenetic eigenvectors as predictors. For each trait, the final model used to impute missing trait values was the one with the higher predictive accuracy (either phylogeny-only, or phylogeny + environmental variables). Note that this amounted to us using the phylogeny-only model for seed dry mass only, and the combined model for all other traits (Supplementary Fig. 6).

### Model performance

Model performance was quantified using buffered leave-one-out cross-validation^46^. To avoid overestimating the out-of-fit accuracy, we first estimated the range of spatial and phylogenetic autocorrelation in the raw data, following the approach recommended in Roberts et al. (2017). Specifically, before running any trait-imputation models, we fit a simple linear regression model to the raw data, where trait expression was modelled as a linear function of phylogenetic and environmental variables. We then assessed spatial autocorrelation of the residuals via Moran’s I plots using the *ncf* package in R, which displays the value of spatial autocorrelation (ranging from −1 to 1) as a function of distance^93^. We likewise assessed residual phylogenetic autocorrelation across taxonomic ranks (genus, family, order, group), using the the *ape* package in R. In general, spatial autocorrelation was low (I < 0.10) (Supplementary Fig. 3), with the exception of leaf phosphorous, which exhibited slight autocorrelation up to ~250 km. The residual phylogenetic correlation was likewise low, and generally only observable at the genus level, apart from crown width and height and conduit diameter, which exhibited residual autocorrelation up to the family level (Supplementary Fig. 4). Thus, to be conservative, for all traits except crown size and conduit diameter, we used a genus-level spatial buffer of 250 km to exclude test/training data; and for crown size and conduit diameter we used a family-level buffer at 250 km.

To implement the cross-validation accuracy assessment, we first randomly selected a focal species, with the out-of-fit test data containing all observations for that species for the focal trait. To construct the corresponding training data, we excluded all observations of the same genus (or family) that fell within a 250 km spatial buffer of any of the training points for that species. The random-forest models were then fit using the buffered training data, and used to predict the trait values for the omitted species^46^. This procedure was repeated for each unique species for each trait, up to 1000 times, with a randomly sampled focal species selected at each iteration. Note that this approach is known to underestimate the actual accuracy^47^, and so the R^2^ values should thus be seen as conservative lower bounds.

Following the recommendation of Li (2017), model accuracy was calculated via the cross-validated coefficient of determination relative to the one-to-one line (termed "VEcv", Li 2017), which provides a normalized version of the mean-squared-error (MSE) that allows for comparisons across data types and units. Specifically, this value is calculated as: ${R^2}_{{VE_{CV}}}=1-{\sum }^{}{({y}_{i}^{{{{{{\mathrm{pred}}}}}}}-{y}_{i}^{{{{{{\mathrm{obs}}}}}}})}^{2}/{\sum }^{}{({y}_{i}^{{{{{{\mathrm{obs}}}}}}}-\bar{y})}^{2}=1-{{{{{\mathrm{SSE}}}}}}/{{{{{\mathrm{TSS}}}}}}=1-{{{{{\mathrm{MSE}}}}}}/{\hat{\sigma }}^{2}$, where the summation is taken across the species, and the predicted values are estimated out-of-fit using the buffered cross-validation procedure outlined above. Importantly, this metric is not the same as a regression-based goodness-of-fit, as it is instead calculated by direct comparison of observed vs. out-of-fit predicted values^94^.

### Principal component analysis

To identify the dominant axes of variation across all 18 traits, we use species-weighted principal component analysis (PCA). This was conducted on the full set of imputed traits using the *aroma.light* package in R, with observed values used in place of imputed values where available. The weights were set to be inversely proportional to the number of observations for each species, which allowed us to incorporate intraspecific variation while also ensuring that each species had the same overall contribution to trait relationships. Representative vectors for each axis were identified by selecting those that loaded most uniquely on each of the first two principle component axes.

### Abiotic relationships

To explore the relationships between the primary trait axes and environmental conditions, we used Shapley values from random-forest models. Shapley values are a game-theoretic metric that partitions the relative influence of each variable on the outcome, for every observation in the dataset^60,95,96^. It is a machine-learning analog to partial regression, in that it looks to quantify the marginal relationship between predictors and outcome variables, taking into account all other variables in the model. To quantify Shapley values, we used the random forest to model each of the PC axis values as a function of the ten environmental variables. We then used the *fastshap* package in R to estimate the Shapley values for each variable for each observation using out-of-fit test dataset (15% of data withheld), and we quantified the overall variable importance by taking the sum of the absolute Shapley values. The environmental variables in Fig. 3a, b are ranked by variable importance, and the trends for the three variables with the highest Shapley values are shown in Fig. 3c–h for each axis. We also conducted a second analysis on the full set of 50 covariates to depict the full set of trends across climate, soil, and topography (Supplementary Figs. 17, 18), but note that many of these relationships are redundant due to high correlations between variables.

### Functional cluster analysis

In order to identify the dimensionality of trait space and identify the dominant functional trait clusters capturing the full variation in tree form and function, we conducted hierarchical clustering on the species-level correlation matrix. First, we calculated species-weighted rank correlations between pairs of traits using the *wCorr* package in R, which again allowed us to incorporate intraspecific trait variation while ensuring each species contributed equal weight. The optimal number of clusters was identified using the silhouette method in the *dendextend* package in R, and the dendrogram was subsequently cut into clusters based on groups of traits which exhibited consistently high average intra-group correlation. As an alternate measure of trait correlation which accounts for phylogenetic relatedness, we calculated phylogenetic independent contrasts^97^ (PIC) on species-level average trait values using the *ape* package. PIC adjusts for the non-independence of species due to their shared evolutionary history, and allows us to remove the effects of pseudo-replication (i.e. among closely related species within a genus) when calculating correlations. The corresponding correlations among these contrasts are shown in the bottom triangle of the correlation matrix in Fig. 4a. Species-level phylogenetic conservatism was calculated using the empirically measured (non-imputed) trait values via Pagel’s λ^98,99^ which quantifies the extent to which trait correlations among species can be explained by their shared evolutionary history, with a value of one equivalent high phylogenetic signal (under Brownian motion), and a value of zero equivalent to no phylogenetic signal (a star phylogeny).

All analyses were conducted in R v. 4.2.0, with the exception of the phylogenetic eigenvector calculations, which were obtained using the *Arpack* package in Julia v. 1.6.2.

### Reporting summary

Further information on research design is available in the Nature Research Reporting Summary linked to this article.

## Supplementary information

Supplementary Information File

Supplementary Data 1

Supplementary Data 2

Reporting Summary

## Data Availability

The imputed trait data used in this study have been deposited in a dedicated GitHub repository with 10.5281/zenodo.6564051 (https://github.com/dsmaynard/tree_traits), along with the cleaned phylogenetic eigenvectors from the seed plant phylogeny of Smith & Brown (2018) and corresponding tree species names matched using BGCI GlobalTreeSearch database v1.3. The raw TRY trait data are not available due to data privacy and sharing restrictions, but can be obtained by making a data request to the TRY Plant Trait Database (https://www.try-db.org/TryWeb/).

## Code availability

The code for replicating the central findings of this paper is available at https://github.com/dsmaynard/tree_traits (10.5281/zenodo.6564051).
