# Supplementary material for: Global relationships in tree functional traits
Source: Nat Commun. 2022 Jun 8;13:3185. doi: 10.1038/s41467-022-30888-2 (PMC9177664; doi:10.1038/s41467-022-30888-2)
Supplement: Supplementary file 1 — Supplementary Information File [file 41467_2022_30888_MOESM1_ESM.pdf]

Supplementary Information File

Global relationships in tree functional traits

Daniel S. Maynard *et al.*

Institute of Integrative Biology, ETH Zürich, 8092 Zürich, Switzerland

Email: dan.s.maynard@gmail.com

CONTENTS:

- Supplementary Figures 1-25
- Supplementary Table 1-5
- Supplementary Notes
- Supplementary References

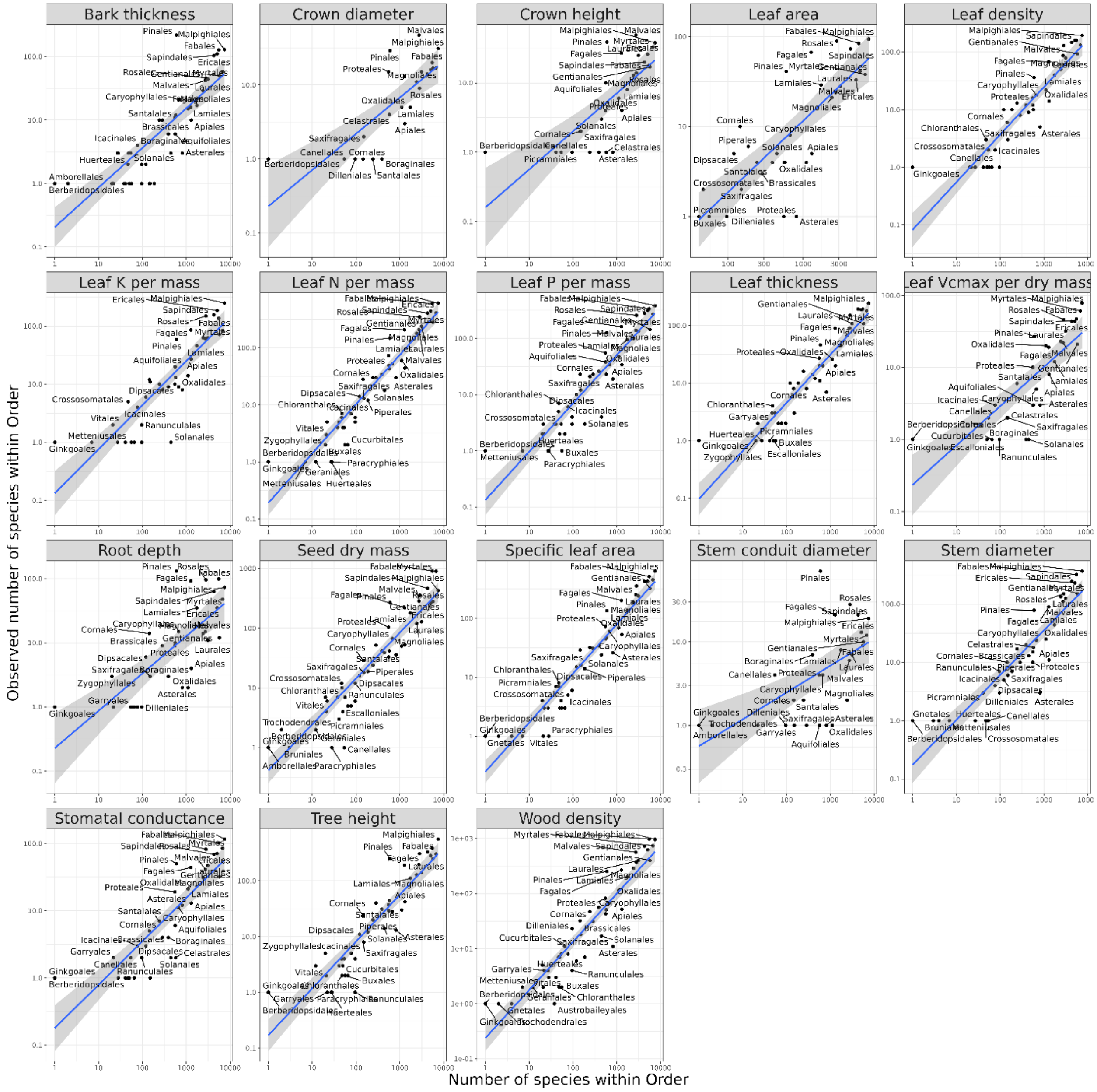

Supplementary Figure 1. The relationships between the number of species within each taxonomic order[21], versus the observed number of species for each trait. The line shows the standard linear regression fit, with the shaded region giving the 95% confidence interval for the mean.

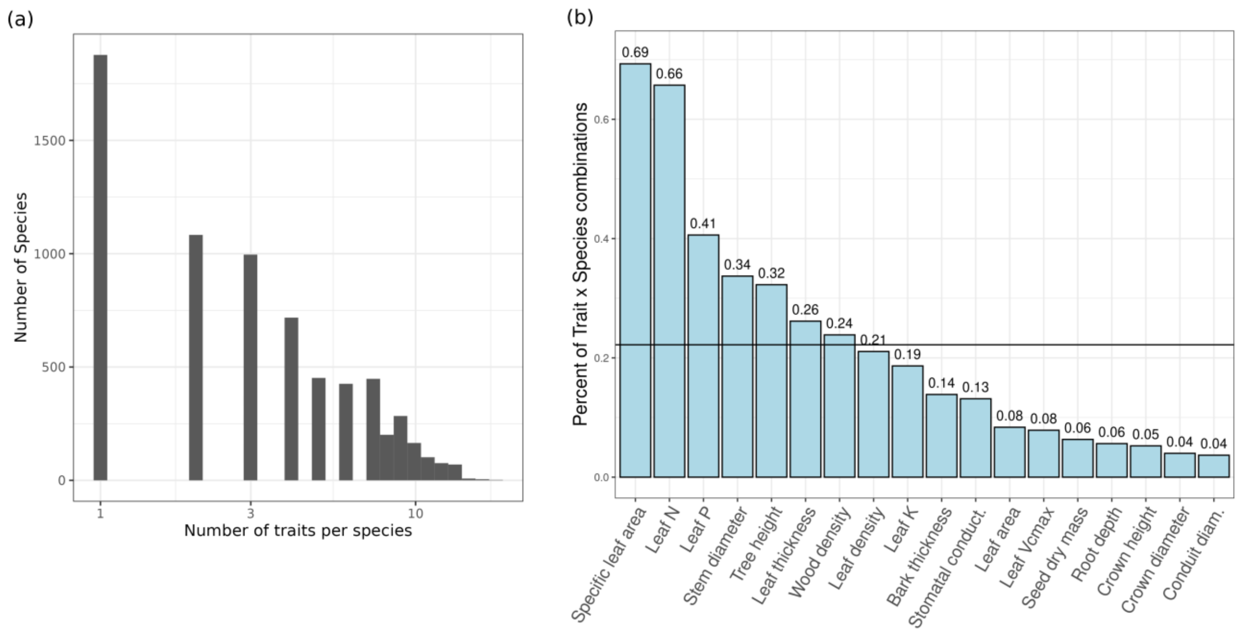

Supplementary Figure 2. **The degree of missingness in the data**. **(a)** The distribution of the number of traits per species. There was an average of 4 traits measured per species, with 423 species having more than 10 unique traits measures, and two species (*Picea abies* and *Pinus sylvestris*) having all traits measured. **(b)** The proportion of unique species with at least one measurement for each trait. The dataset captured 22% of all trait-by-species combinations (horizontal black line), slightly better than other large-scale trait analyses across the entire plant kingdom[30,31].

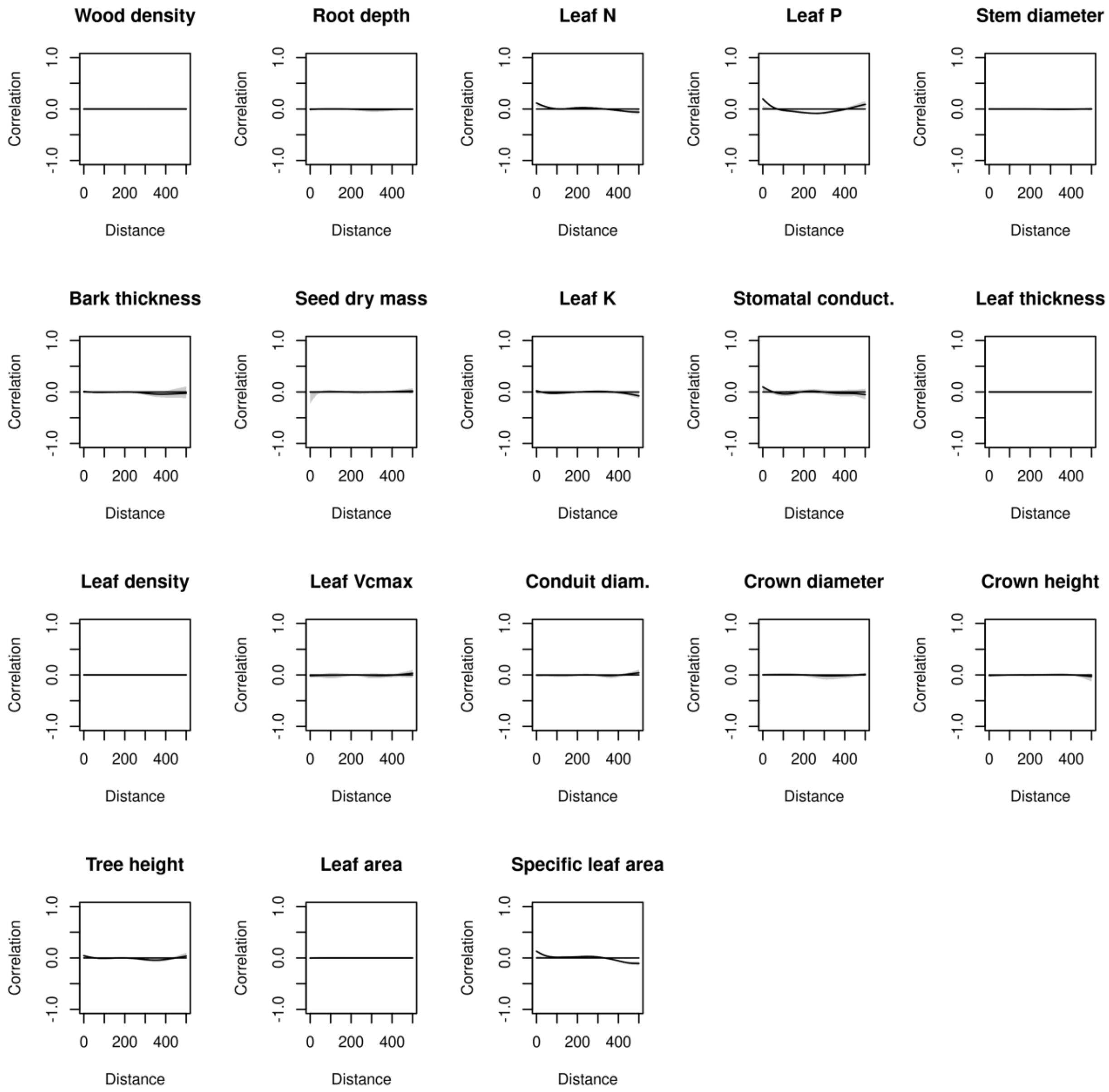

Supplementary Figure 3. Residual spatial autocorrelation (Moran's I) for all 18 traits, assessed using standard linear regression models.

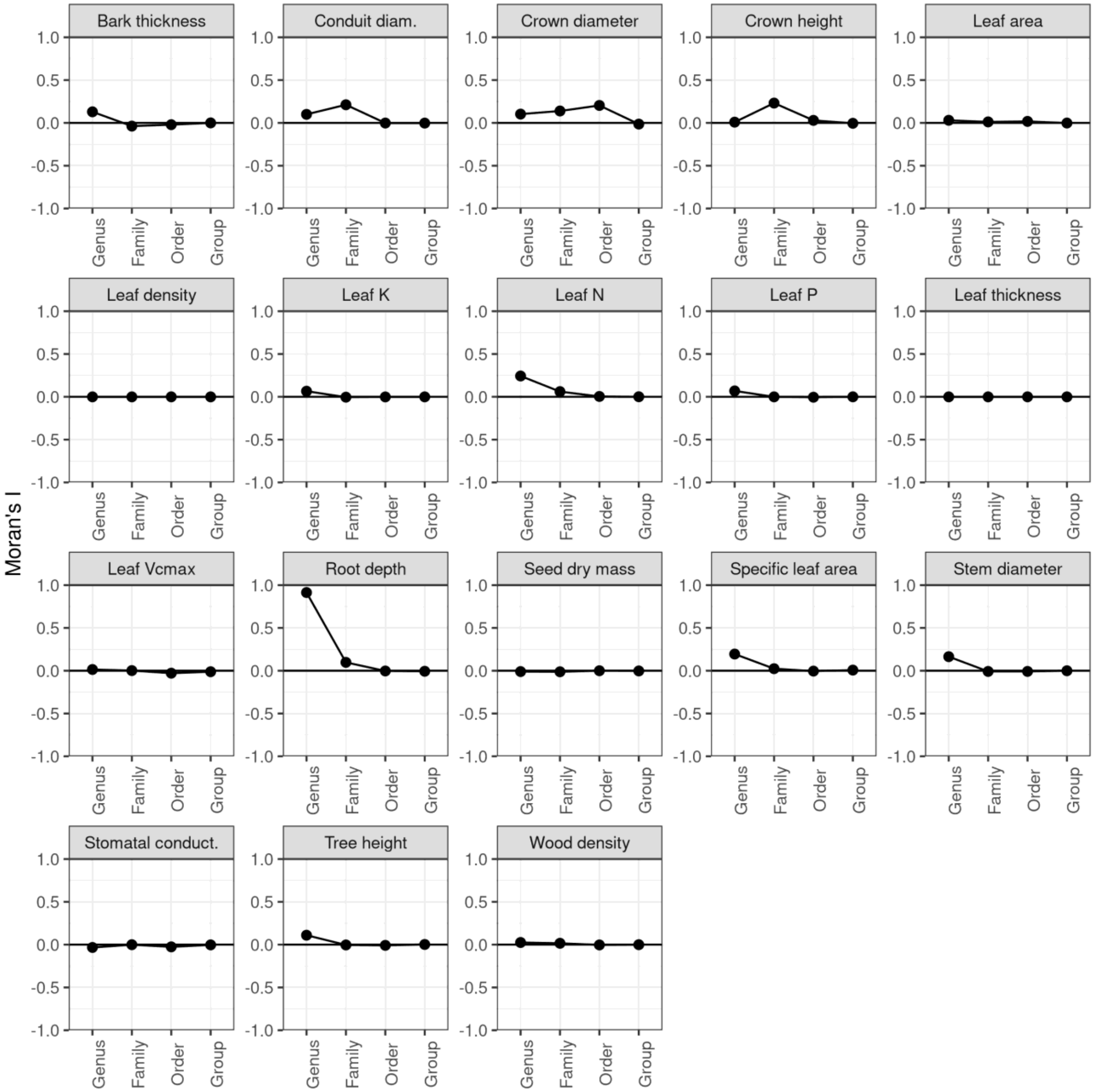

Supplementary Figure 4. Residual taxonomic autocorrelation (Moran's I) for all 18 traits, assessed using standard linear regression models.

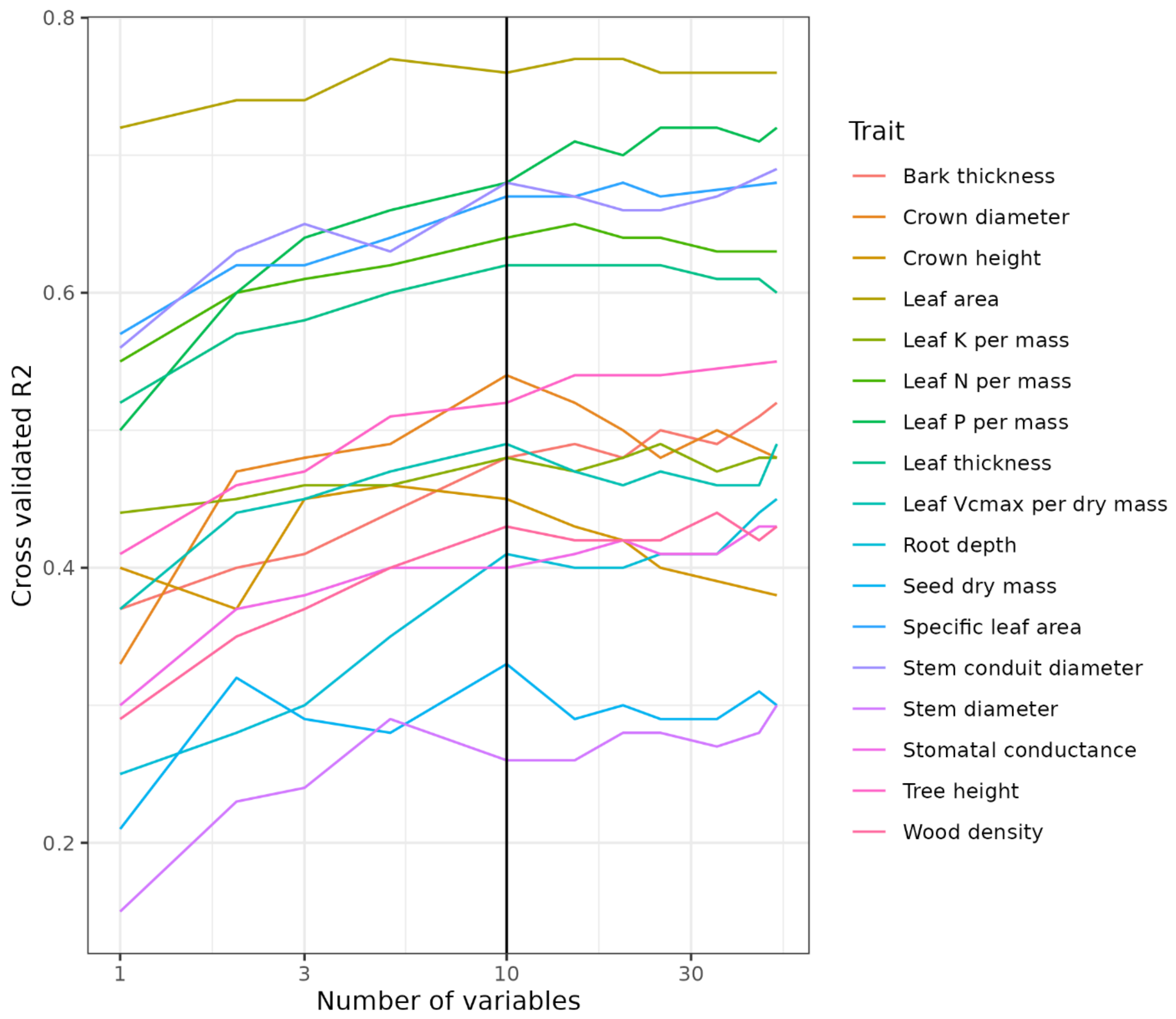

Supplementary Figure 5. The out-of-fit R2 as a function of the number of environmental and phylogenetic predictors in the random forest model. The final models used 10 environmental and phylogenetic predictors, which exhibited consistency high R^2 values while also prioritizing model parsimony to avoid overfitting.

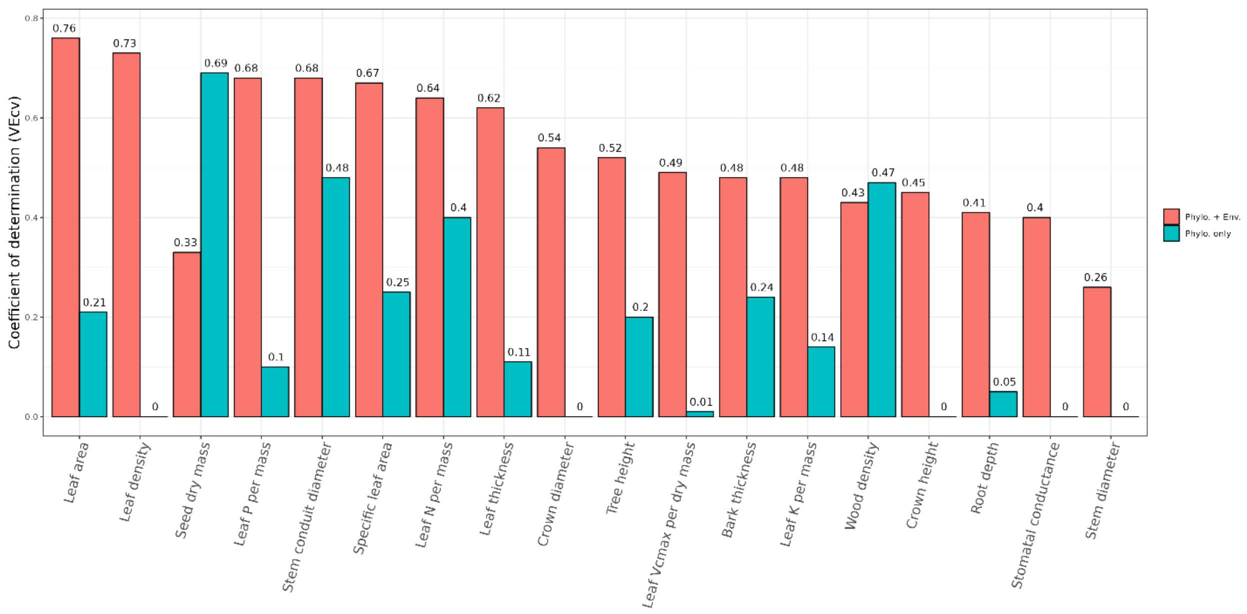

Supplementary Figure 6. The performance of the models (R^2_{VEcv}) with phylogenetic information only (green) vs. phylogenetic and environmental covariates included (red).

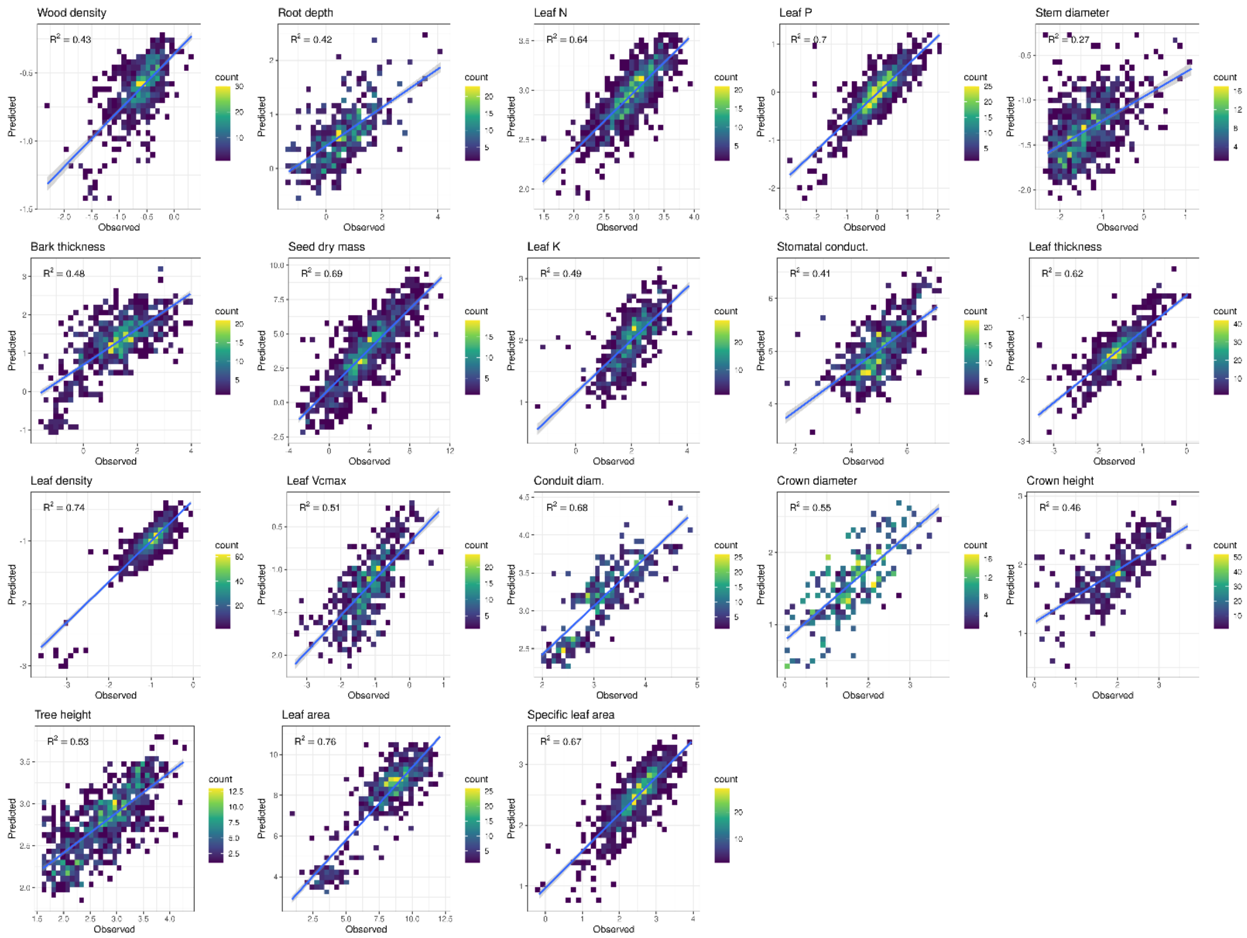

Supplementary Figure 7. The observed vs. predicted values for the out-of-fit data. The highest accuracy was with leaf area (R^2 = 0.76) with the lowest being stem diameter (R^2 = 0.27), with the latter reflecting the fact that stem diameter is closely related to tree age. Hence, for traits with strong ontogenetic variation (stem diameter, tree height, root depth, crown height, crown width) we used quantile random forest to predict the maximum values (see Methods). The points are shaded by density (yellow = many points in that pixel; blue = few). The line shows the standard linear regression fit, with the shaded region giving the 95% confidence interval for the mean.

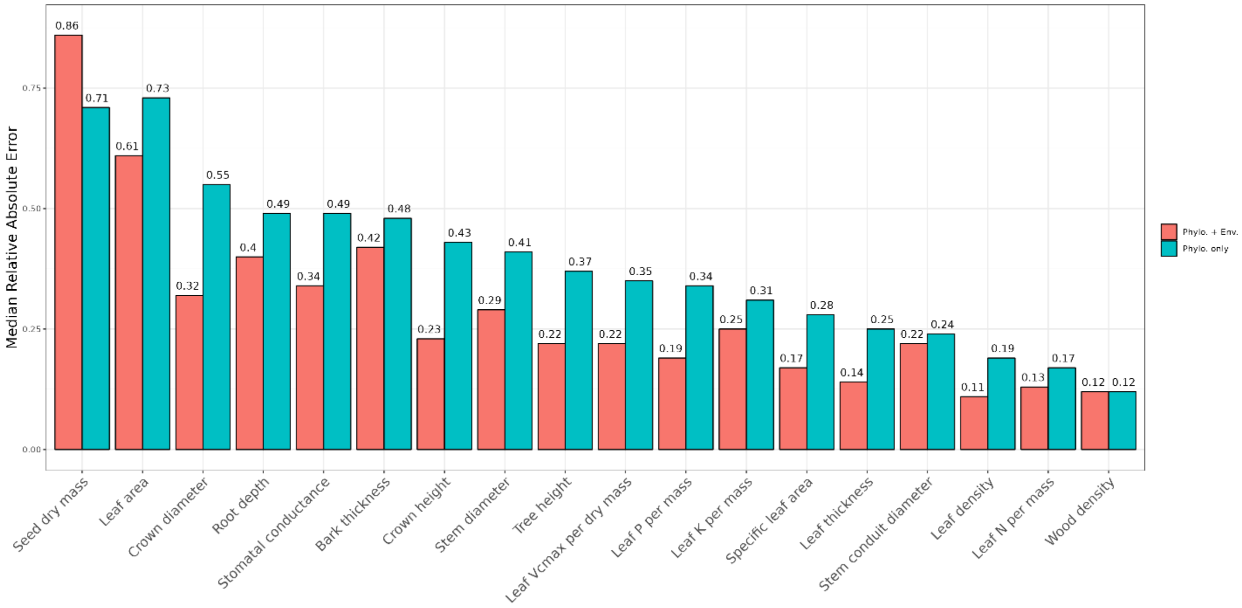

Supplementary Figure 8. Trait accuracy on the unlogged scale, assessed via median relative absolute error, with phylogenetic information only (green) vs. phylogenetic and environmental covariates included (red). Note that because most traits exhibit skewed log-normal distributions, where the sample variance for an observation is correlated with the mean, the relative error on the unlogged scale is largely an intrinsic artifact of the skewness of the data. Thus, regardless of the accuracy of the models (Supplementary Fig. 6), care should be taken when using these approaches to make inference about the unlogged trait expression of a specific tree in a specific location.

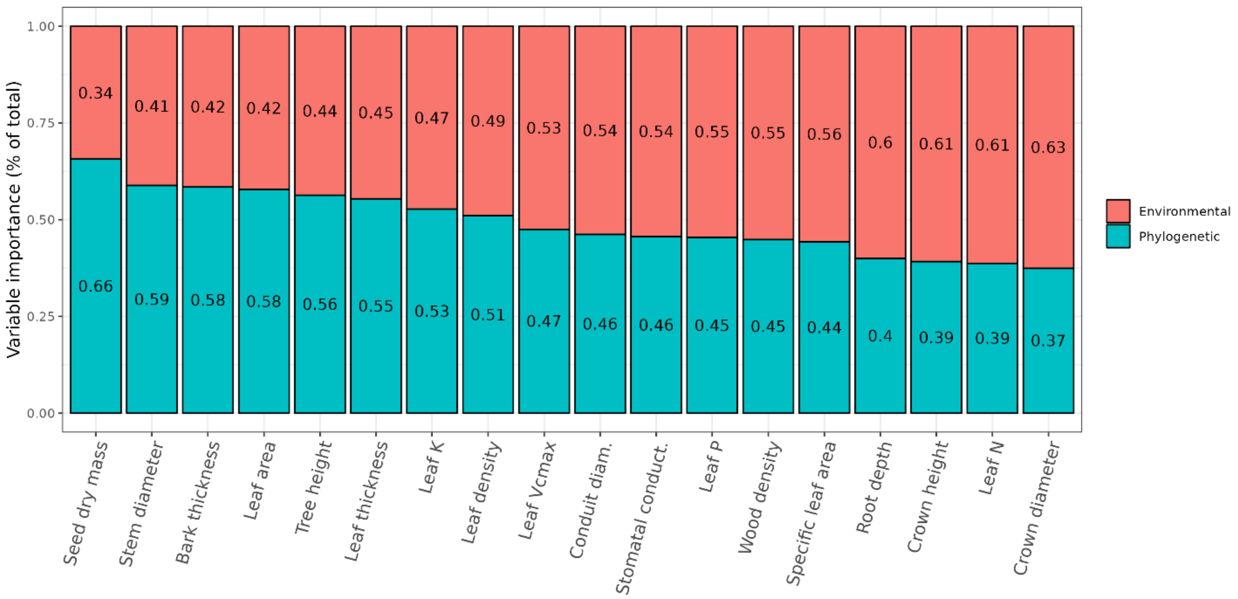

Supplementary Figure 9. The relative importance (scaled to 1) attributable to environmental variables vs. phylogenetic eigenvectors as predictors of trait expression.

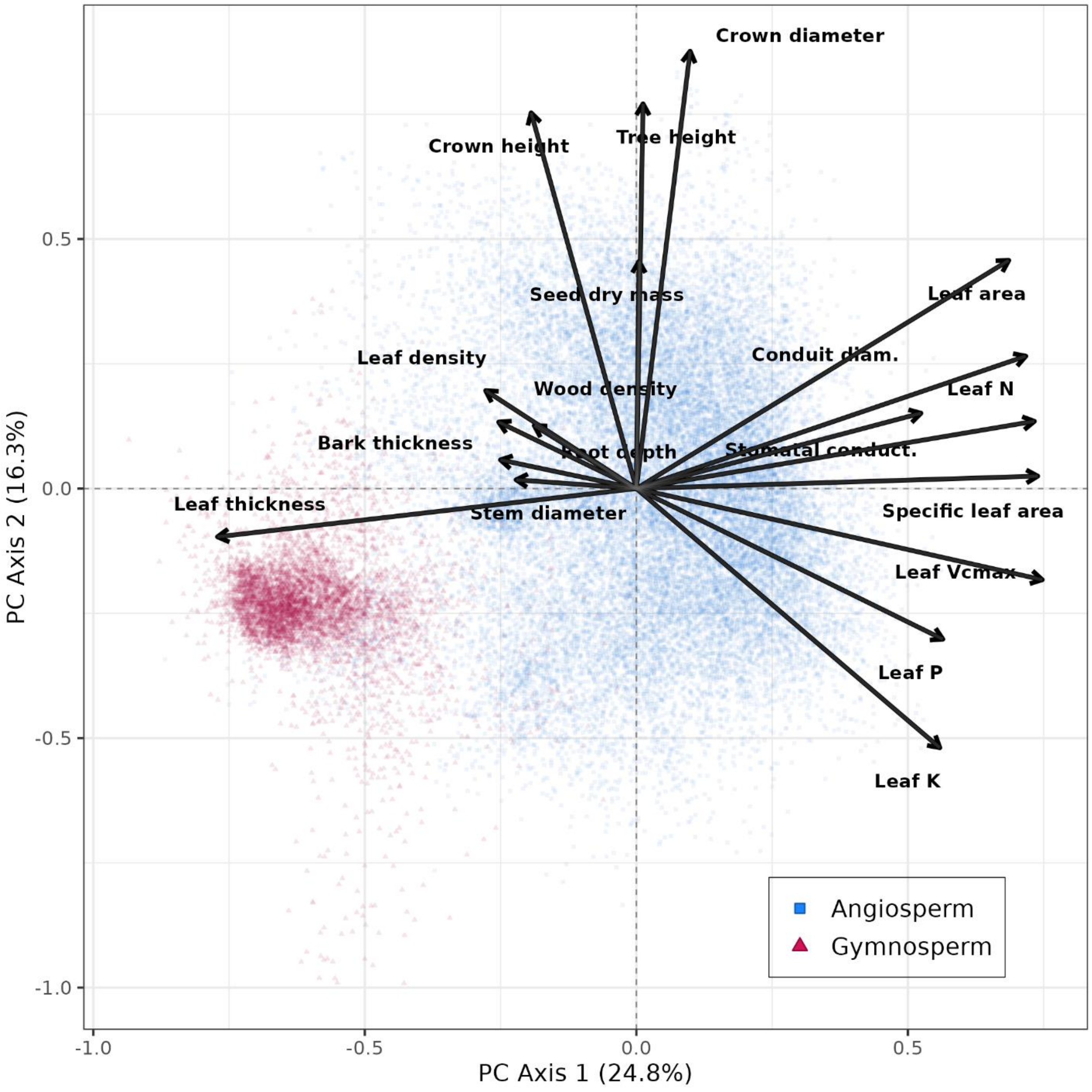

Supplementary Figure 10. The same PCA as in the main text (Fig. 2a), but with all trait axes labeled. See Supplementary Table 5 for the loadings.

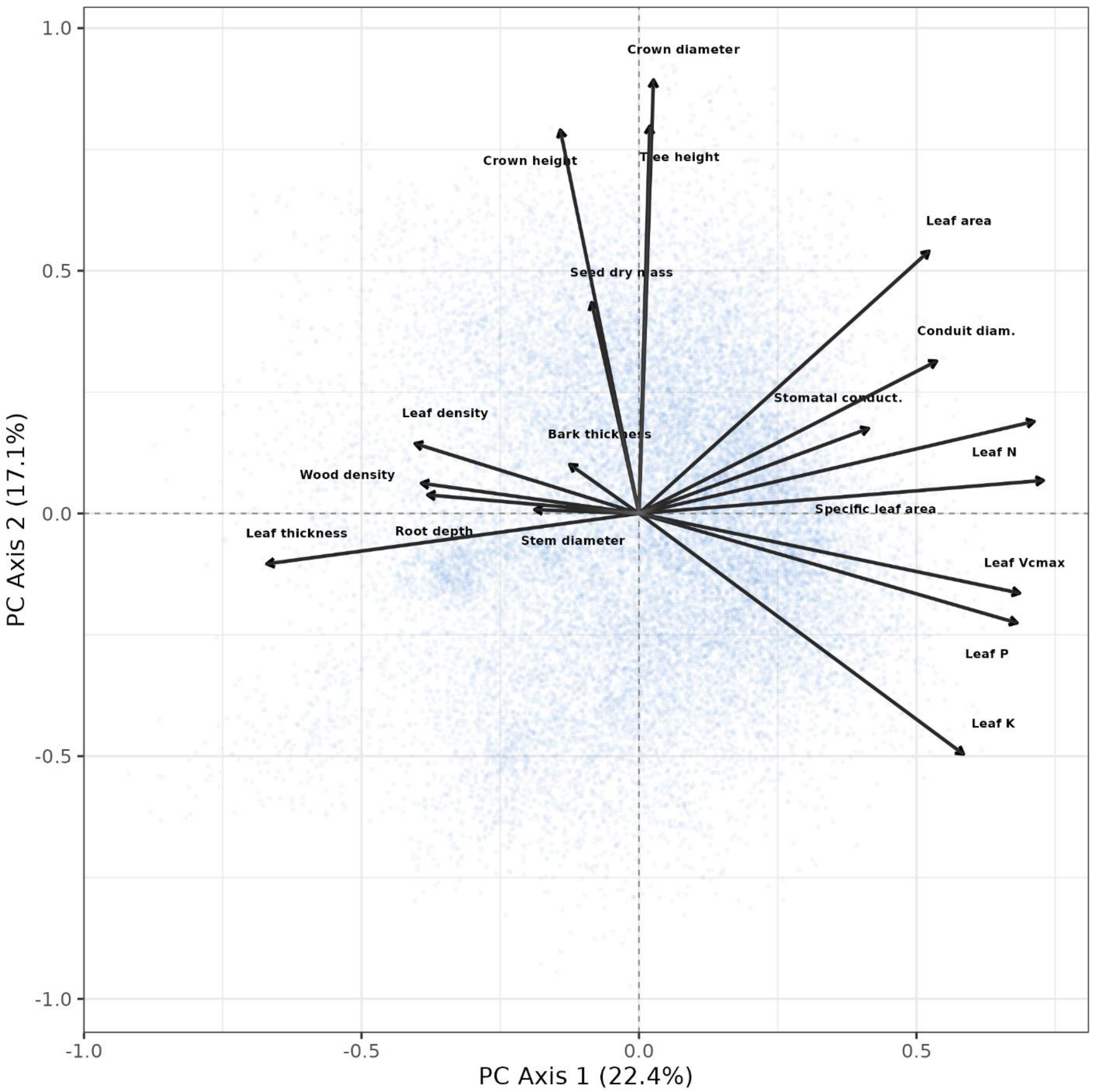

Supplementary Figure 11. The same PCA as in the main text (Fig. 2b), but with all trait axes labeled. See Supplementary Data 2 for the PC loadings table.

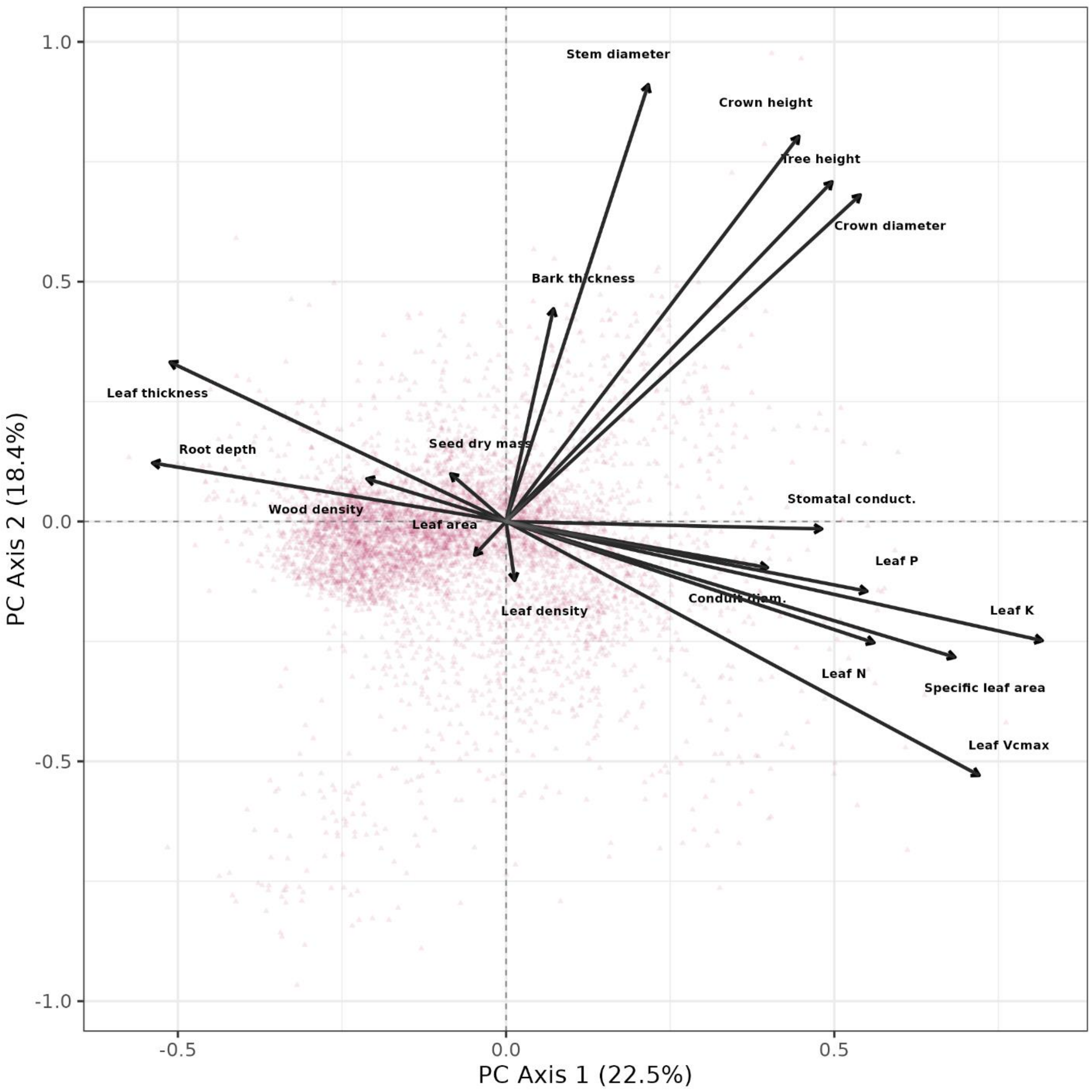

Supplementary Figure 12. The same PCA as in the main text (Fig. 2c), but with all trait axes labeled. See Supplementary Data 2 for the PC loadings table.

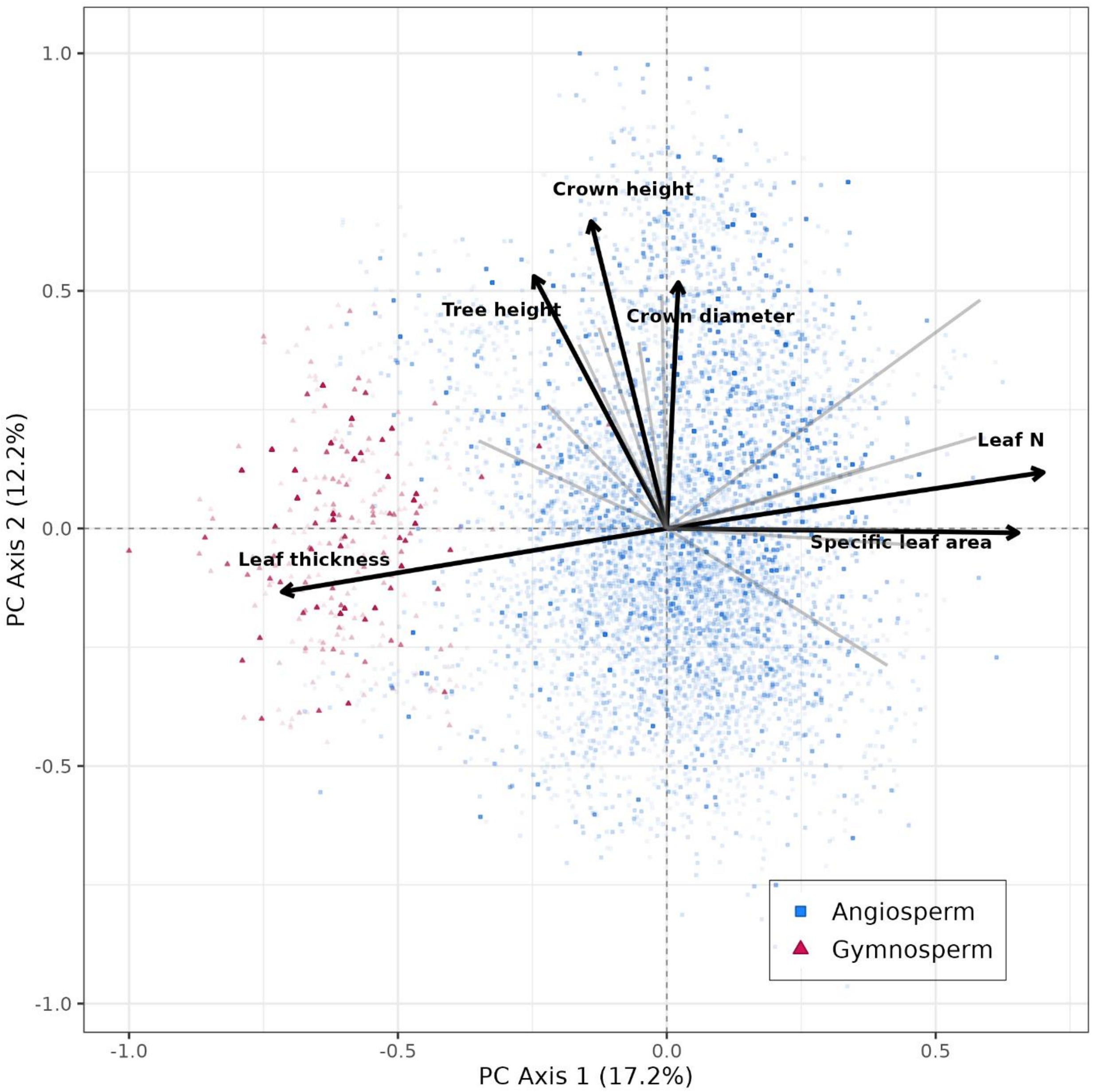

Supplementary Figure 13. The PCA using phylogeny-only models, showing the same trade-offs as with the environmental + phylogeny models, despite having substantially lower predictive accuracy of the trait models. See Supplementary Data 2 for the PC loadings table.

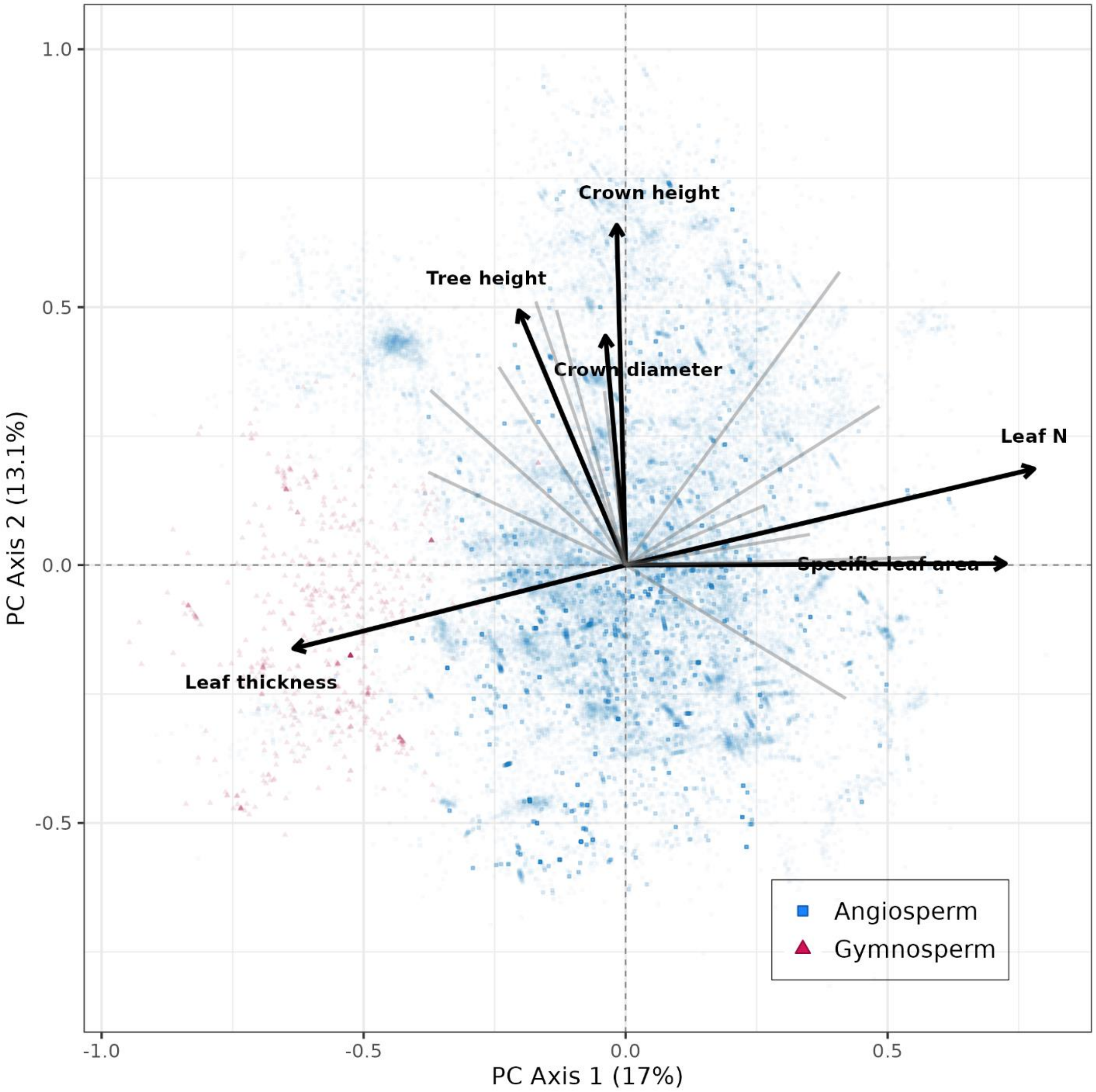

Supplementary Figure 14. The PCA for all 52,255 tree species matched in the reference phylogeny, using the phylogeny-only models, showing the same trade-offs as with the subset of species with georeferenced data. See Supplementary Data 2 for the PC loadings table.

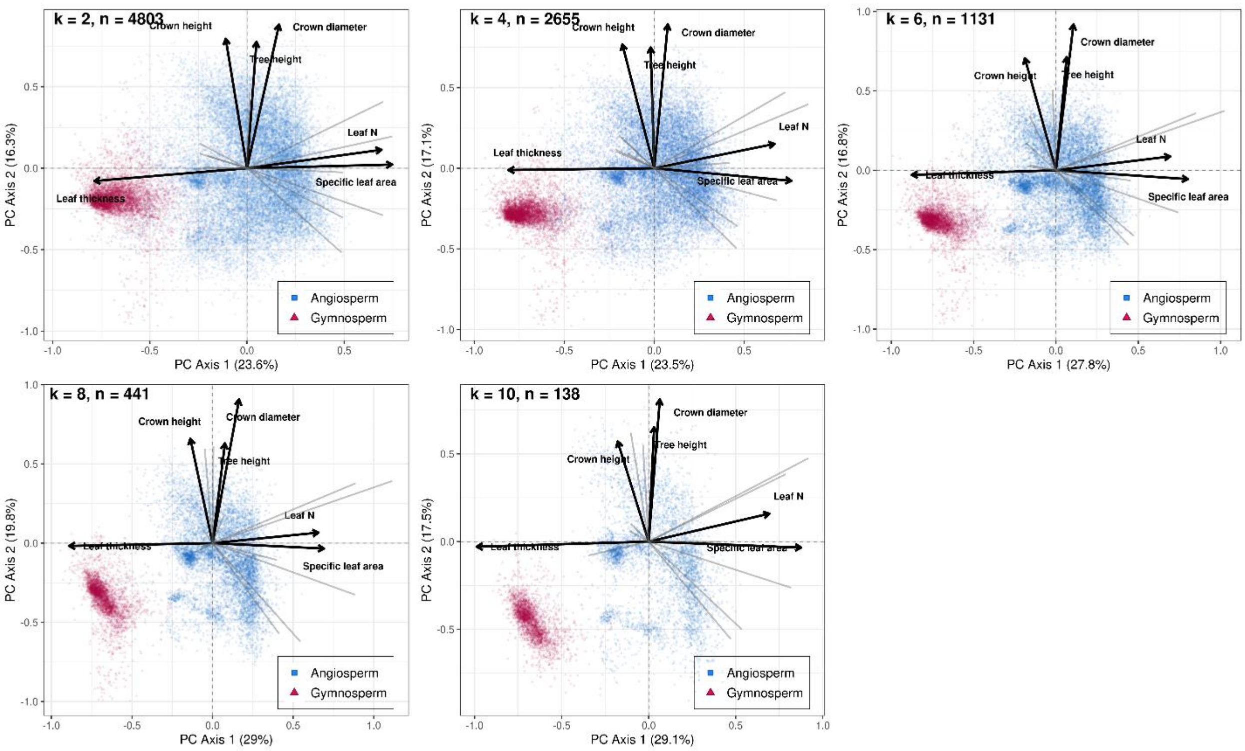

Supplementary Figure 15. The PCA results using decreasing levels of missingness in the dataset, ranging from all species with at least 2 traits measured (n = 4803), to only those with at least 10 unique traits measurements (n=138 species). The results are highly robust to the level of missingness in the data. See Supplementary Data 2 for the PC loadings table.

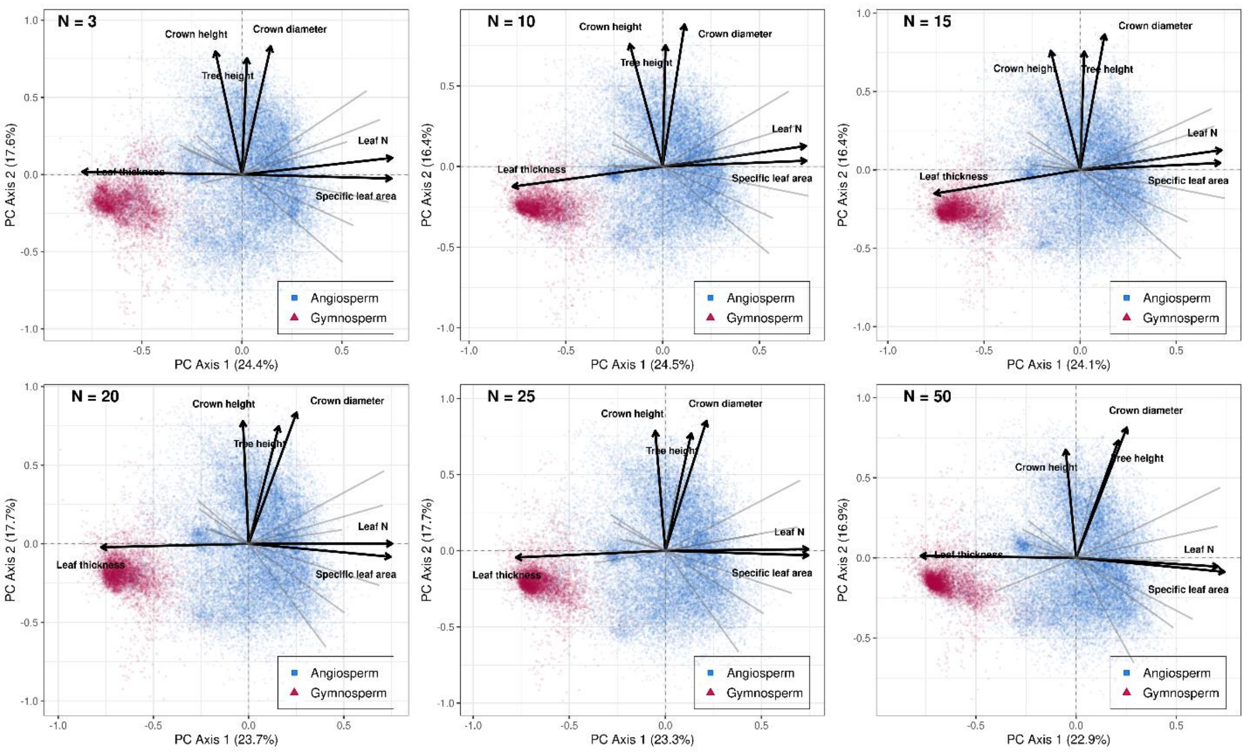

Supplementary Figure 16. The PCA results using increasing number of environmental covariates, ranging from 3 to 50 (each) phylogenetic and environmental covariates. N=10 is used throughout the main text (see Supplementary Fig. 5). The results are robust to the choice of variables. See Supplementary Data 2 for the PC loadings table.

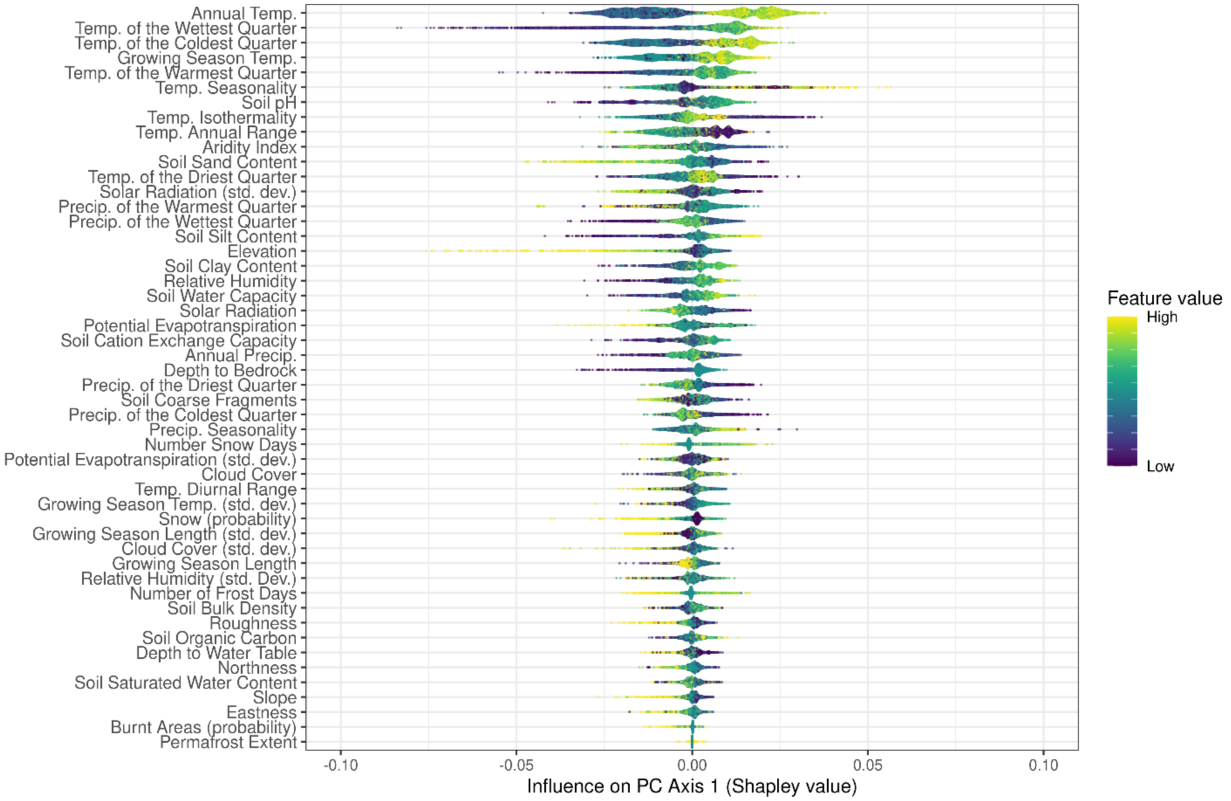

Supplementary Figure 17. The Shapley values for all 50 environmental variables' influence on PC 1, sorted by variable importance. Note that some of these variables are highly collinear, and thus reflect redundant patterns. See the main text for the 10 representative variables with low correlation.

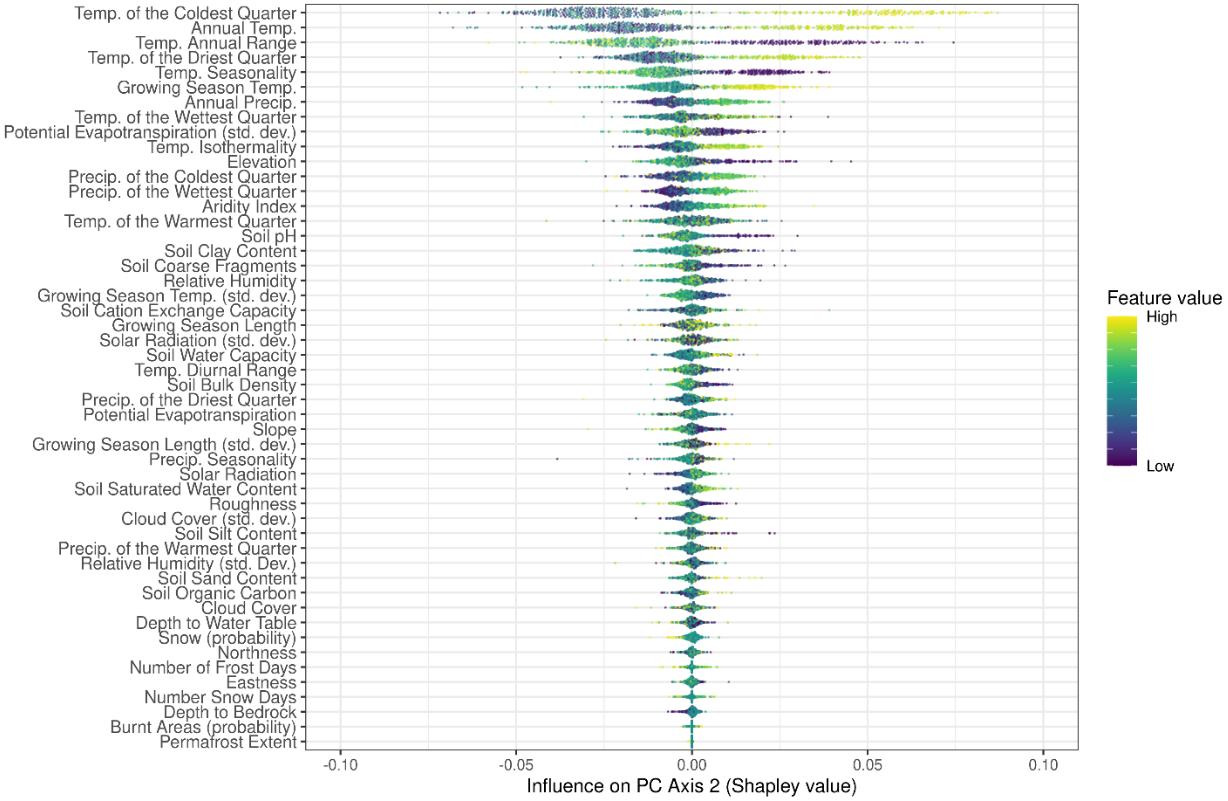

Supplementary Figure 18. The Shapley values for all 50 environmental variables' influence on PC 2, sorted by variable importance. Note that some of these variables are highly collinear, and thus reflect redundant patterns. See the main text for the 10 representative variables with low correlation.

	Leaf density	Wood density	Root depth	Specific leaf area	Leaf thickness	Leaf N	Leaf K	Leaf P	Conduit diam.	Leaf Vcmax	Stomatal conduct.	Leaf area	Crown height	Crown diameter	Tree height	Seed dry mass	Bark thickness	Stem diameter
Leaf density		0.43	0.2	-0.48	0.23	-0.28	-0.31	-0.31	0.06	-0.1	0	0.02	0.03	0.05	-0.01	0.22	0.26	0.08
Wood density	0.33		0.27	-0.22	0.08	-0.16	-0.26	-0.31	-0.01	-0.07	0.04	-0.03	0.02	0.08	-0.07	0.15	0.05	-0.01
Root depth	0.08	0.01		-0.2	0.11	-0.07	-0.23	-0.26	0.02	-0.08	-0.14	-0.06	-0.06	0.06	-0.01	0.17	0.04	-0.01
Specific leaf area	-0.42	-0.14	-0.18		-0.82	0.55	0.38	0.45	0.25	0.44	0.14	0.37	-0.08	0.08	0.06	0.01	-0.32	-0.29
Leaf thickness	0.19	0.07	0.11	-0.72		-0.51	-0.31	-0.42	-0.26	-0.41	-0.1	-0.36	0.04	-0.1	-0.06	-0.1	0.35	0.29
Leaf N	-0.24	-0.09	-0.1	0.39	-0.31		0.42	0.52	0.47	0.48	0.32	0.34	0.05	0.21	0.11	0.07	-0.18	-0.14
Leaf K	-0.17	-0.1	-0.11	0.26	-0.22	0.27		0.71	0.26	0.56	0.25	0.04	-0.43	-0.41	-0.36	-0.22	-0.07	-0.01
Leaf P	-0.22	-0.14	-0.16	0.32	-0.3	0.34	0.58		0.29	0.51	0.22	0.1	-0.17	-0.21	-0.14	-0.1	-0.02	0.01
Conduit diam.	0.15	0.08	0.03	0.02	0.01	0.09	0.06	0.04		0.64	0.59	0.62	-0.06	0.21	0.1	0.12	0.24	0.11
Leaf Vcmax	0.01	0.07	0	0.3	-0.27	0.22	0.33	0.26	0.44		0.51	0.45	-0.31	-0.15	-0.21	-0.15	0.15	0.12
Stomatal conduct.	0.1	0.14	-0.15	-0.02	0.04	0.07	0.06	0.08	0.39	0.31		0.45	-0.05	0.14	0.1	-0.05	0.18	0.09
Leaf area	0.09	0.11	-0.06	0.15	-0.12	0.05	-0.16	-0.1	0.54	0.31	0.31		0.08	0.37	0.26	0.13	0.11	-0.05
Crown height	-0.06	-0.06	-0.21	-0.07	0.06	0.08	-0.3	-0.14	-0.14	-0.33	0.05	0.06		0.78	0.64	0.23	0.03	0.14
Crown diameter	-0.05	-0.03	-0.19	0.04	-0.03	0.11	-0.33	-0.18	0.06	-0.2	0.14	0.29	0.74		0.7	0.3	-0.06	-0.03
Tree height	-0.06	-0.09	-0.11	0.02	-0.01	0.04	-0.29	-0.12	0.01	-0.24	0.14	0.16	0.52	0.58		0.28	0.03	0.14
Seed dry mass	0.02	-0.01	0.03	-0.02	0.01	-0.03	-0.03	-0.02	0	-0.06	0.01	0.02	0.04	0.04	0.03		-0.05	-0.11
Bark thickness	0.27	0.17	0.03	-0.22	0.21	-0.08	-0.03	-0.04	0.52	0.25	0.31	0.29	-0.05	-0.01	0.06	-0.01		0.51
Stem diameter	0	-0.03	-0.04	-0.2	0.22	-0.1	-0.06	-0.01	0.15	0.02	0.12	0.04	0.2	0.09	0.25	-0.02	0.37	

Supplementary Figure 19. The full set of correlations between the imputed trait values. The upper triangle gives the Spearman correlation between imputed values; the lower triangle gives the correlation between phylogenetic independent contrasts, thereby controlling for highly related species with similar phylogenetic histories.

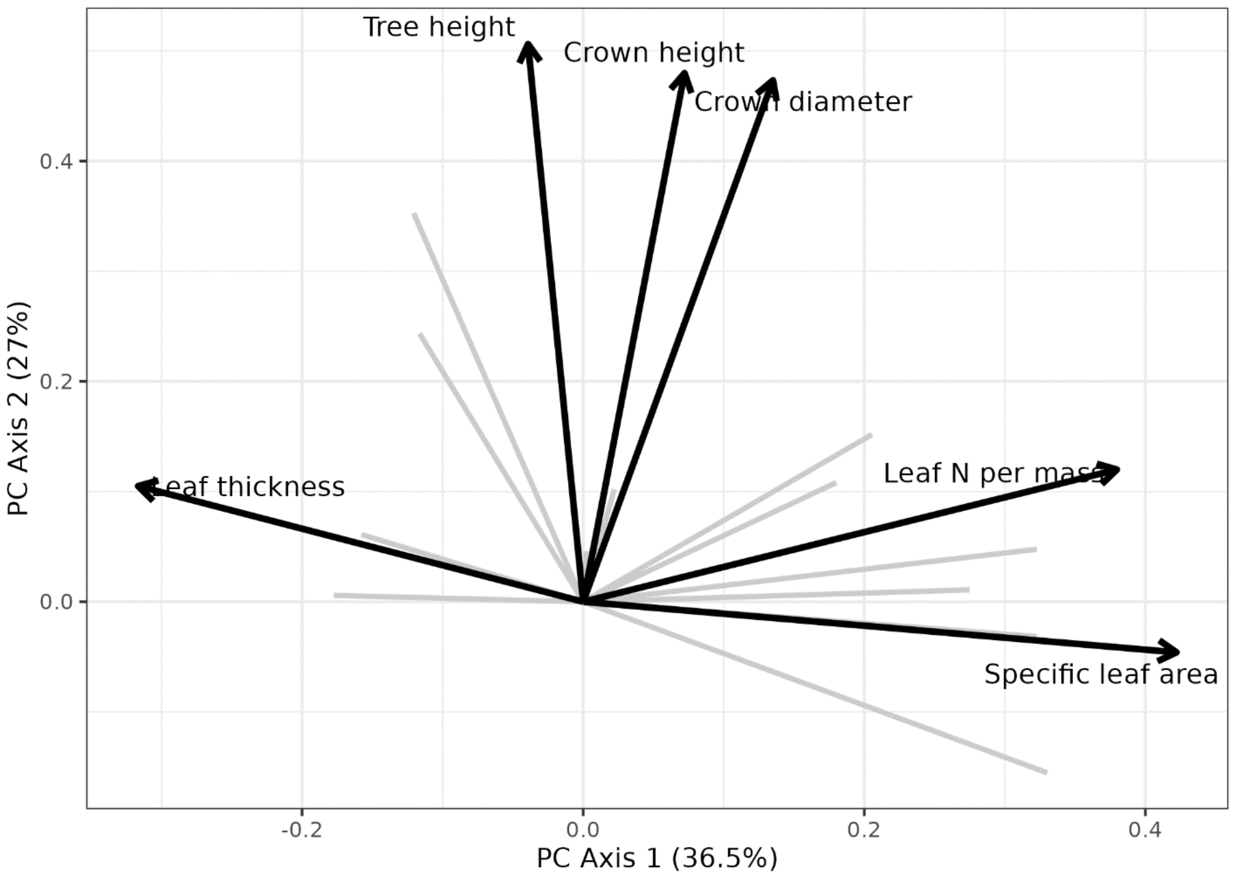

Supplementary Figure 20. The PCA using raw data only. Since PCA is implemented via calculating the eigenvectors of the correlation matrix, we can recreate this PCA using only the raw data. Here, we calculated the correlations using only the pairwise complete raw observations for each pair of traits. We then took the eigen-decomposition of this correlation matrix, with the first two eigenvectors show here, along with % variation. Using raw data only, we see the identical 6 traits load most heavily on the two axes.

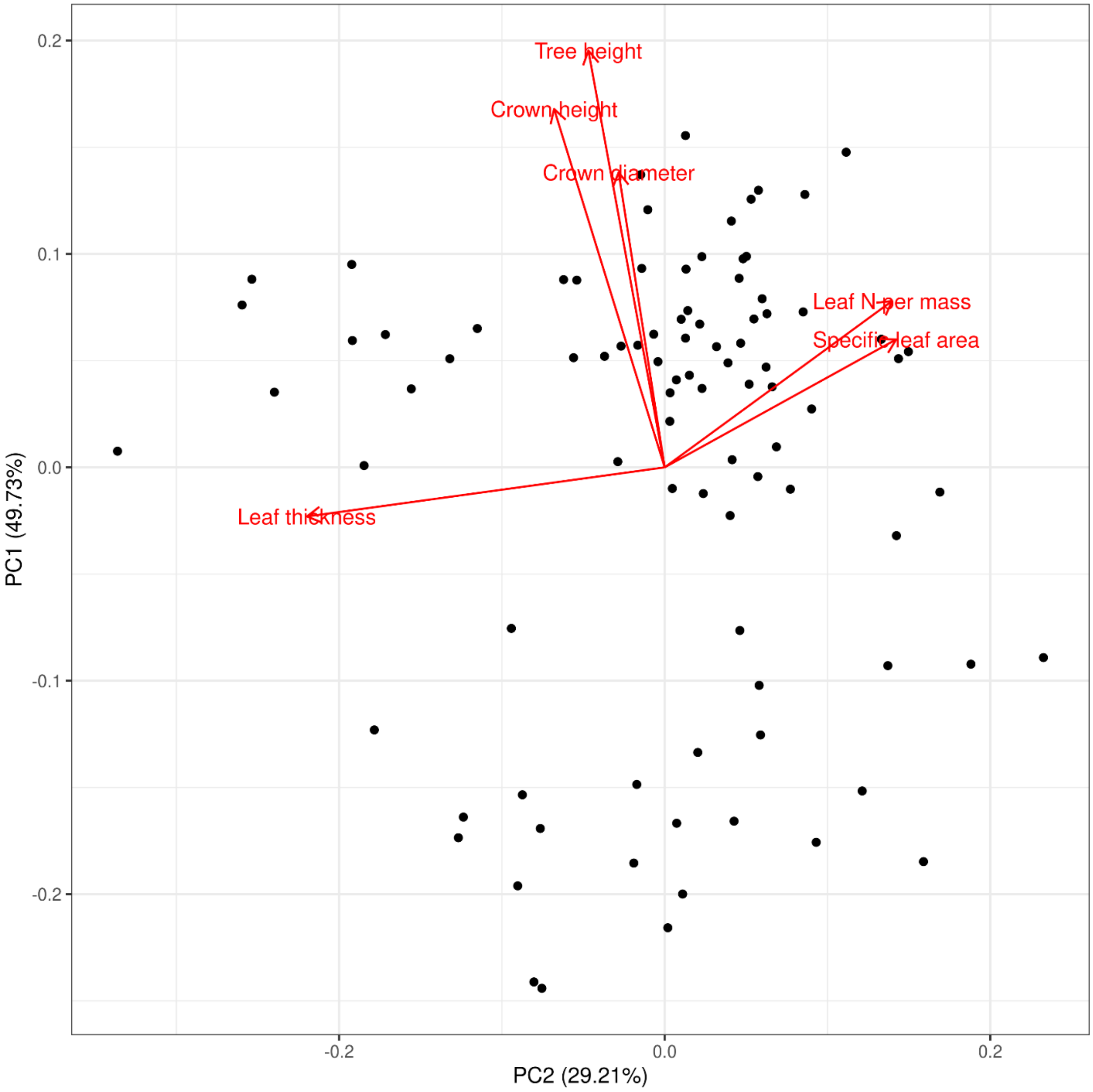

Supplementary Figure 21. The PCA results when restricted to the 91 species which have complete trait values for the six dominant traits underpinning the PC axes in the full dataset. Using this small subset of data, we see the same orthogonality of the trait axes. Moreover, we recover the same environmental patterns using these PC results (Supplementary Fig. 22).

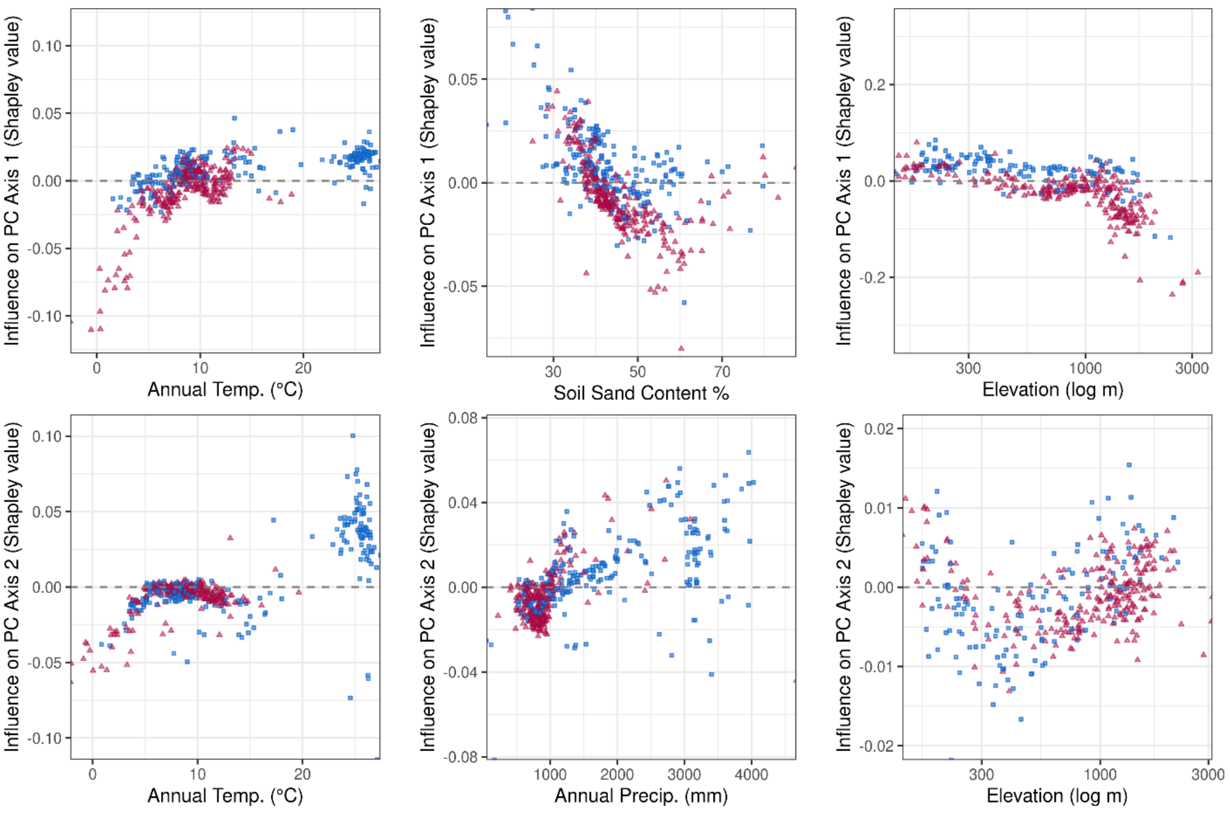

Supplementary Figure 22. The relationships between the first two PC axes and the dominant environmental drivers shown in the main text, obtained using raw data only, using the 91 species and 3319 observations where we had complete data for the dominant 6 traits underpinning the PC axes (see Supplementary Fig. 21).

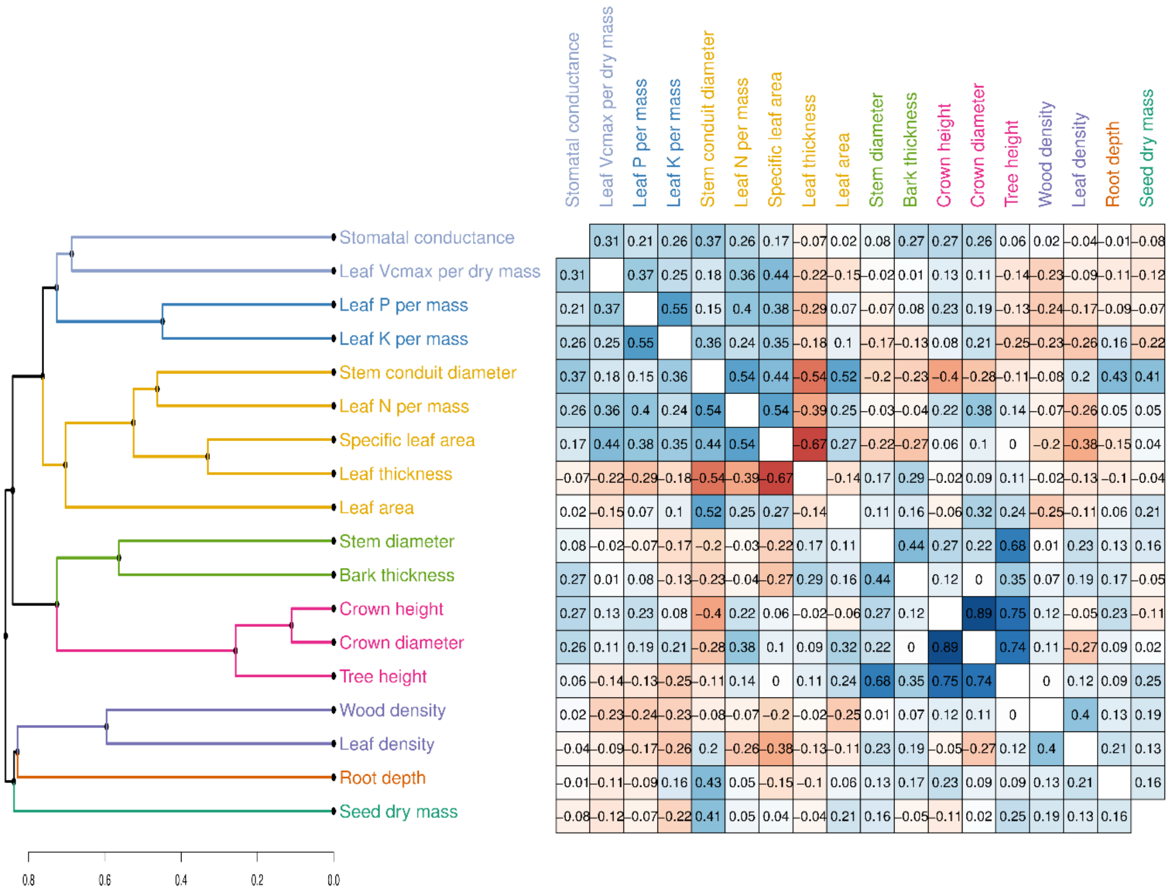

Supplementary Figure 23. The functional dendrogram obtained using pairwise-complete raw observations. The results show nearly the identical trends and relationships to the full set of imputed data, despite being species-level averages.

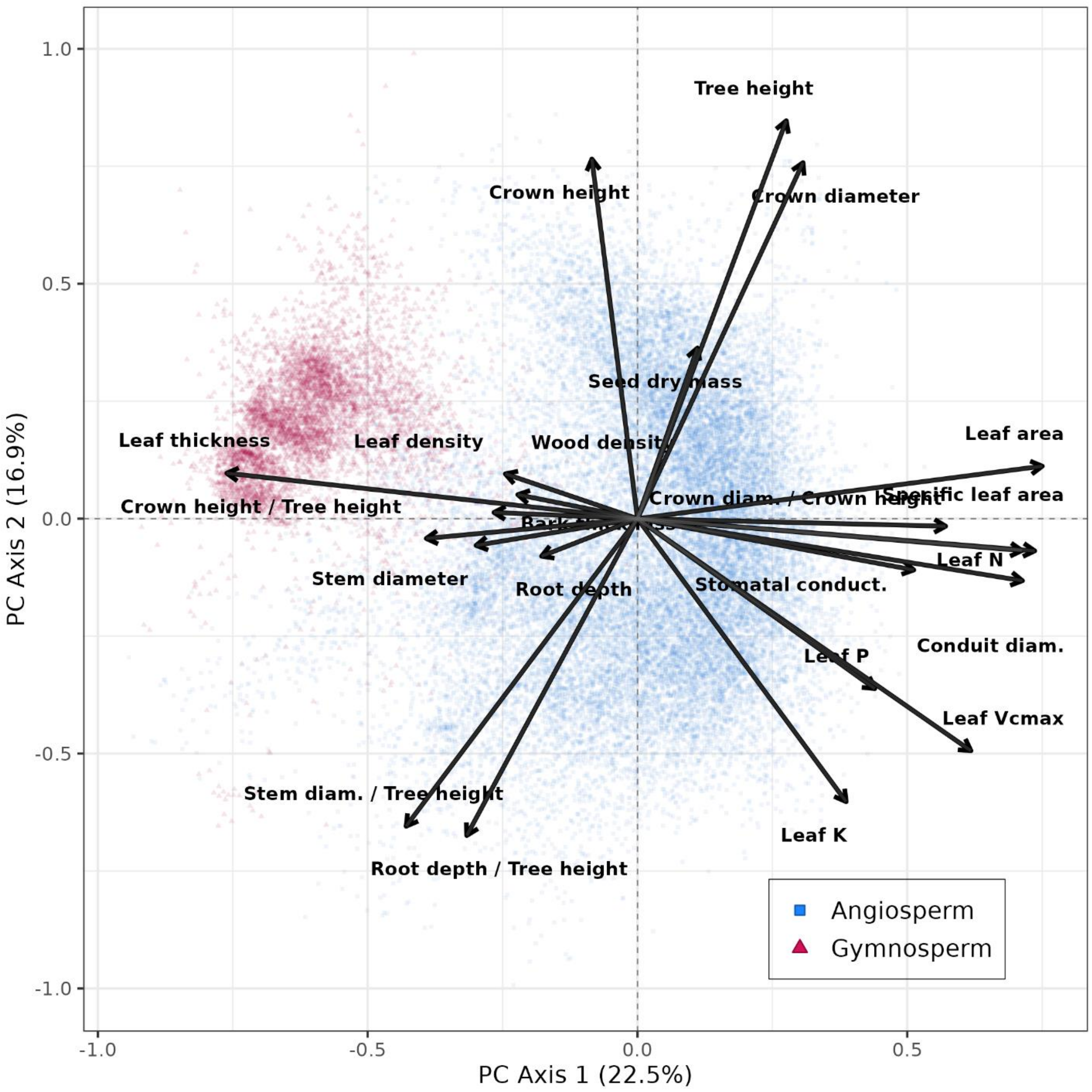

Supplementary Figure 24. The trait PC when adding four allometric relationship estimated using the imputed data (crown height / tree height; stem diameter / tree height; root depth / tree height, crown diameter / crown height). The dominant axes and representative traits are unchanged, highlighting that these allometric ratios contribute no new information to the dominant axes underpinning trait relationships. Note that these allometric ratios are estimated and their accuracy cannot be assessed directly, and so caution should be exercised when interpreting them.

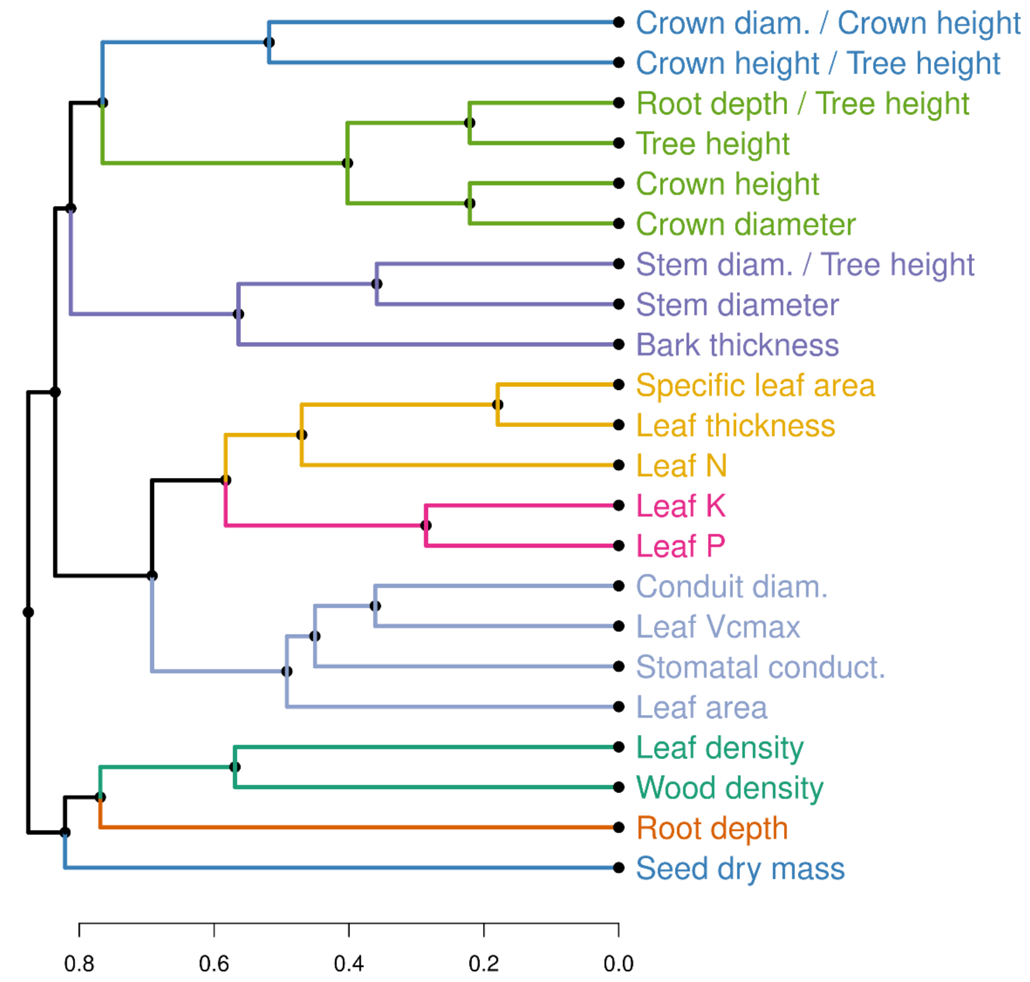

Supplementary Figure 25. The functional dendrogram when four allometric ratios (Supplementary Fig. 24) are added to the dataset. The ratios “root depth / tree height” and “stem diam. / tree height” ratio join within existing groups, whereas “crown diam. / crown height” and “crown height. / tree height” form their own unique cluster reflecting crown architecture. Note that these allometric ratios are estimated and their accuracy cannot be assessed directly, and so caution should be exercised when interpreting them.

Supplementary Table 1. The number of observations per trait by continental region, with the percentage in parentheses denoting the proportion of these observations taken on angiosperm species. For reference, angiosperms account for ~99% of all extant tree species[20,21].

Trait	Global	South America	Eurasia	Africa	Oceania	North America	No spatial coords.
All	491001 (85%)	200578 (99.8%)	94585 (57.8%)	16736 (99.6%)	15036 (92.8%)	61755 (62.4%)	102311 (91.2%)
Bark thickness	6786 (73.4%)	3945 (99.5%)	390 (97.7%)	86 (100%)	303 (93.1%)	1484 (14.7%)	578 (15.1%)
Crown diameter	4293 (68.3%)	939 (100%)	1373 (70.6%)	862 (100%)	64 (100%)	1043 (8.1%)	12 (100%)
Crown height	5218 (57.6%)	1035 (99.8%)	2430 (63.3%)	112 (100%)	101 (100%)	1486 (11.9%)	54 (83.3%)
Leaf area	14002 (96.9%)	5476 (100%)	2668 (96.1%)	444 (100%)	770 (100%)	4329 (93.1%)	315 (88.9%)
Leaf density	21810 (97.6%)	17041 (100%)	862 (83.9%)	648 (100%)	479 (96.5%)	2055 (81.8%)	725 (100%)
Leaf K per mass	7204 (75.2%)	2543 (100%)	1253 (89.1%)	430 (100%)	2 (50%)	1597 (12.5%)	1379 (81.7%)
Leaf N per mass	60617 (81.2%)	21752 (99.6%)	17410 (67.8%)	2815 (99.4%)	2287 (88.2%)	9719 (67.1%)	6634 (66.1%)
Leaf P per mass	26411 (73.3%)	7552 (99.9%)	10152 (62.5%)	821 (100%)	1023 (93.7%)	2535 (31.7%)	4328 (66.6%)
Leaf thickness	55497 (98.3%)	42351 (100%)	1326 (85.7%)	6412 (100%)	486 (99%)	3575 (79.9%)	1347 (98.1%)
Leaf Vcmax per dry mass	2028 (91%)	561 (98.9%)	322 (66.5%)	674 (100%)	193 (77.2%)	154 (84.4%)	124 (99.2%)
Root depth	2346 (68.6%)	151 (100%)	396 (51%)	466 (97%)	82 (63.4%)	604 (46.9%)	647 (72.6%)
Seed dry mass	42650 (96.2%)	291 (100%)	100 (88%)	28 (100%)	38 (97.4%)	96 (85.4%)	42097 (96.2%)
Specific leaf area	70968 (90.6%)	26810 (99.7%)	16459 (82%)	2109 (99%)	3286 (92.6%)	17220 (82.5%)	5084 (93.7%)
Stem conduit diameter	508 (68.5%)	179 (99.4%)	113 (46%)	20 (95%)	24 (62.5%)	168 (48.2%)	4 (75%)
Stem diameter	53168 (85.6%)	36422 (99.9%)	7550 (72.3%)	220 (90.9%)	829 (86%)	7995 (33.6%)	152 (57.2%)
Stomatal conductance	29084 (39.6%)	1491 (98.3%)	18778 (14.8%)	30 (100%)	3772 (96.9%)	4401 (67.2%)	612 (99.7%)
Tree height	41865 (84.9%)	25718 (99.7%)	6276 (45.7%)	251 (100%)	1259 (89.8%)	2584 (45.8%)	5777 (77.2%)
Wood density	46546 (88.7%)	6321 (99.4%)	6727 (43.7%)	308 (100%)	38 (63.2%)	710 (48.3%)	32442 (96.8%)

Supplementary Table 2. The number of species per trait by continental region, with the percentage in parentheses denoting the proportion of these species that are angiosperms. For reference, angiosperms account for ~99% of all extant tree species[20,21].

Trait	Global	South America	Eurasia	Africa	Oceania	North America	No spatial coords.
**All**	13189 (96.9%)	3559 (99.1%)	2063 (93.5%)	597 (98.5%)	585 (92.8%)	542 (83.8%)	10644 (96.3%)
**Bark thickness**	1134 (80.8%)	725 (97.8%)	70 (90%)	9 (100%)	131 (84.7%)	34 (47.1%)	196 (6.6%)
**Crown diameter**	182 (87.9%)	58 (100%)	85 (92.9%)	19 (100%)	2 (100%)	19 (10.5%)	2 (100%)
**Crown height**	289 (88.9%)	111 (98.2%)	148 (92.6%)	1 (100%)	8 (100%)	22 (13.6%)	3 (66.7%)
**Leaf area**	733 (94.4%)	327 (100%)	165 (91.5%)	56 (100%)	4 (100%)	49 (93.9%)	289 (87.9%)
**Leaf density**	1566 (97.6%)	918 (100%)	186 (90.9%)	93 (100%)	119 (95%)	169 (90.5%)	145 (100%)
**Leaf K per mass**	1587 (96.2%)	658 (100%)	385 (95.6%)	127 (100%)	2 (50%)	132 (93.2%)	391 (88.7%)
**Leaf N per mass**	4777 (96.9%)	2309 (99.4%)	1356 (94.8%)	425 (99.1%)	261 (96.2%)	356 (85.7%)	1037 (91.2%)
**Leaf P per mass**	3165 (96%)	1253 (99.8%)	989 (93.7%)	233 (100%)	195 (95.9%)	203 (83.7%)	1020 (92.1%)
**Leaf thickness**	1942 (97.6%)	1133 (99.8%)	282 (92.6%)	127 (100%)	124 (96%)	215 (90.7%)	310 (97.7%)
**Leaf Vcmax per dry mass**	550 (94.9%)	323 (98.5%)	50 (82%)	42 (100%)	99 (96%)	36 (72.2%)	21 (95.2%)
**Root depth**	794 (83.5%)	60 (100%)	132 (48.5%)	103 (94.2%)	19 (89.5%)	95 (74.7%)	554 (79.1%)
**Seed dry mass**	5468 (95.1%)	285 (100%)	50 (84%)	28 (100%)	35 (97.1%)	41 (70.7%)	5327 (94.9%)
**Specific leaf area**	4896 (97.2%)	2497 (99.4%)	1362 (96%)	275 (98.9%)	414 (96.1%)	441 (85.3%)	551 (88.6%)
**Stem conduit diameter**	259 (72.6%)	70 (98.6%)	60 (60%)	18 (94.4%)	21 (71.4%)	94 (54.3%)	4 (75%)
**Stem diameter**	2063 (96.4%)	1509 (99.7%)	433 (92.4%)	41 (97.6%)	25 (96%)	89 (60.7%)	55 (65.5%)
**Stomatal conductance**	915 (94.4%)	508 (98.4%)	129 (89.9%)	7 (100%)	144 (96.5%)	136 (80.1%)	25 (96%)
**Tree height**	3313 (92.6%)	1154 (99.7%)	378 (89.7%)	75 (100%)	154 (91.6%)	127 (71.7%)	1865 (87.6%)
**Wood density**	7491 (96.6%)	1256 (99.5%)	211 (86.3%)	62 (100%)	34 (67.6%)	121 (57.9%)	6858 (96.7%)

Supplementary Table 3. The 30 functional traits and corresponding TRY trait IDs and sub-trait IDs. Of these 30 putative traits, the 18 traits used in final the analysis (indicated with *) were selected based on uniqueness, consistency of assay conditions, taxonomic coverage, geographic coverage, and overall sample size. Note that several traits (e.g., Trait 4, wood density) have multiple corresponding sub-trait IDS. Data are included from more than 130 publications (Supplementary Data 1).

TRY Trait ID	Sub-trait Data IDs	Trait Name
4*	4<br>1629<br>1739<br>2568	Stem specific density (SSD) or wood density
6*	7	Root rooting depth
9	10	Root/shoot ratio
14*	15	Leaf nitrogen (N) content per leaf dry mass
15*	16	Leaf phosphorus (P) content per leaf dry mass
21*	24	Stem diameter
24*	28	Bark thickness
26*	30	Seed dry mass
41	46	Leaf respiration rate in the dark per leaf dry mass
44*	49	Leaf potassium (K) content per leaf dry mass
45*	50	Stomatal conductance per leaf area
46*	53	Leaf thickness
48*	55	Leaf density (leaf tissue density, leaf dry mass per leaf volume)
56	100	Leaf nitrogen/phosphorus (N/P) ratio
80	272	Root nitrogen (N) content per root dry mass
144	446	Leaf length
145	447	Leaf width
146	455	Leaf carbon/nitrogen (C/N) ratio
185*	549<br>2382	Leaf photosynthesis carboxylation capacity (Vcmax) per leaf dry mass
270	664	Leaf photosynthesis electron transport capacity (Jmax) per leaf dry mass
281*	713	Stem conduit diameter (vessels, tracheids)
324*	818	Crown (canopy) length: diameter along the longest axis
413	996	Leaf chlorophyll content per leaf area
773*	1695	Crown (canopy) height (base to top)
1055	1950	Root carbon/nitrogen (C/N) ratio
1229	2657	Wood nitrogen (N) content per wood dry mass
3106*	19<br>448<br>504	Plant height vegetative
3110*	6577	Leaf area (in case of compound leaves: leaf, petiole included)
3117*	6584<br>6598	Leaf area per leaf dry mass (specific leaf area, SLA or 1/LMA)
3120	2261	Leaf water content per leaf dry mass (not saturated)

Supplementary Table 4. The full set of 50 environmental covariates used as predictors in the random forest models.

Variable	Source	Type	Units	Resolution
Annual Temp.	1	Climatic	ºC	30 arcsec (≈900m at equator)
Temp. of the Coldest Quarter	1	Climatic	ºC	30 arcsec (≈900m at equator)
Temp. of the Driest Quarter	1	Climatic	ºC	30 arcsec (≈900m at equator)
Temp. of the Warmest Quarter	1	Climatic	ºC	30 arcsec (≈900m at equator)
Temp. of the Wettest Quarter	1	Climatic	ºC	30 arcsec (≈900m at equator)
Temp. Annual Range	1	Climatic	ºC	30 arcsec (≈900m at equator)
Temp. Seasonality	1	Climatic	ºC	30 arcsec (≈900m at equator)
Temp. Isothermality	1	Climatic	Unitless	30 arcsec (≈900m at equator)
Temp. Diurnal Range	1	Climatic	ºC	30 arcsec (≈900m at equator)
Aridity Index	2	Climatic	AI Value	≈1km
Potential Evapotranspiration	2	Climatic	PET Value (mm)	≈1km
Annual Precip.	1	Climatic	mm	30 arcsec (≈900m at equator)
Precip. of the Coldest Quarter	1	Climatic	mm	30 arcsec (≈900m at equator)
Precip. of the Driest Quarter	1	Climatic	mm	30 arcsec (≈900m at equator)
Precip. of the Warmest Quarter	1	Climatic	mm	30 arcsec (≈900m at equator)
Precip. of the Wettest Quarter	1	Climatic	mm	30 arcsec (≈900m at equator)
Precip. Seasonality	1	Climatic	mm	30 arcsec (≈900m at equator)
Potential Evapotranspiration (std. dev.)	1	Climatic	PET Value (mm)	30 arcsec (≈900m at equator)
Relative Humidity	1	Climatic	% * 100	30 arcsec (≈900m at equator)
Relative Humidity (std. Dev.)	1	Climatic	% * 100	30 arcsec (≈900m at equator)
Growing Season Length	1	Climatic	number of days	30 arcsec (≈900m at equator)
Growing Season Length (std. dev.)	1	Climatic	number of days	30 arcsec (≈900m at equator)
Growing Season Temp.	1	Climatic	ºC	30 arcsec (≈900m at equator)
Growing Season Temp. (std. dev.)	1	Climatic	ºC	30 arcsec (≈900m at equator)
Number of Frost Days	1	Climatic	Number of days	30 arcsec (≈900m at equator)
Number of Snow Days	1	Climatic	number of days	30 arcsec (≈900m at equator)
Solar Radiation	1	Climatic	kJ m-2	30 arcsec (≈900m at equator)
Solar Radiation (std. dev.)	1	Climatic	kJ m-2	30 arcsec (≈900m at equator)
Cloud Cover	3	Climatic	% cloudy days	30 arcsec (≈900m at equator)
Cloud Cover (std. dev.)	3	Climatic	% cloudy days	30 arcsec (≈900m at equator)
Burnt Areas (probability)	4	Climatic	Proportion of burned areas	≈500m
Snow (probability)	4	Climatic	Proportion of snow occurrence	≈500m
Permafrost Extent	5	Climatic	Unitless	30 arcsec (≈900m at equator)
Depth to Water Table	6	Geological	m below land surface	30 arcsec (≈900m at equator)
Depth to Bedrock	7	Geological	cm (up to 200)	≈250m
Soil Bulk Density	7	Soil	kg / cubic–meter	≈250m
Soil Cation Exchange Capacity	7	Soil	cmolc/kg	≈250m
Soil Clay Content	7	Soil	mass fraction in %	≈250m
Soil Coarse Fragments	7	Soil	%	≈250m
Soil Water Capacity	7	Soil	%	≈250m
Soil Organic Carbon	7	Soil	g per kg	≈250m
Soil Sand Content	7	Soil	mass fraction in %	≈250m
Soil Saturated Water Content	7	Soil	%	≈250m
Soil Silt Content	7	Soil	mass fraction in %	≈250m
Soil pH	7	Soil	pH x 10	≈250m
Eastness	3	Topography	eastness index (-1 to 1)	30 arcsec (≈900m at equator)
Elevation	3	Topography	meters	30 arcsec (≈900m at equator)
Northness	3	Topography	northness index (-1 to 1)	30 arcsec (≈900m at equator)
Roughness	3	Topography	(see reference)	30 arcsec (≈900m at equator)
Slope	3	Topography	(see reference)	30 arcsec (≈900m at equator)

1. CHELSA [22,23]
2. CGIAR [24]
3. EarthEnv [25]
4. ESA CCI [26]
5. Obu *et al.* 2019
6. Fan *et al.* 2013
7. Soilgrids [29]

Supplementary Table 5. The PCA loading for each of the 18 traits and the 18 PC axes, along with the % variances explained by each axis. The variable that loads most heavily on each axis is shown in gray.

	PC1	PC2	PC3	PC4	PC5	PC6	PC7	PC8	PC9	PC10	PC11	PC12	PC13	PC14	PC15	PC16	PC17	PC18
**% Variance Explained**	**24.8**	**16.3**	**11.4**	**9.3**	**5.3**	**5.2**	**4.3**	**4**	**3.5**	**2.9**	**2.6**	**2.2**	**2.1**	**1.9**	**1.4**	**1.3**	**0.8**	**0.7**
**Bark thickness**	-0.25	0.06	-0.5	-0.45	0.17	-0.05	-0.05	0.03	-0.07	0.54	-0.03	0.11	-0.06	0.1	0.1	0.04	-0.04	0.02
**Conduit diam.**	0.72	0.27	-0.47	0.03	-0.1	-0.04	-0.15	-0.05	0.01	-0.09	-0.13	-0.07	-0.12	-0.02	-0.01	0.36	0.02	-0.09
**Crown diameter**	0.1	0.88	0.14	-0.08	-0.08	-0.07	0.14	-0.07	-0.14	-0.03	-0.13	-0.01	0.08	0.11	-0.04	0.07	0.12	0.24
**Crown height**	-0.19	0.75	0.25	-0.29	0.11	-0.08	0.18	-0.01	-0.15	-0.1	-0.14	0.07	0.21	0.14	0.05	-0.02	-0.08	-0.2
**Leaf area**	0.69	0.46	-0.31	0.06	-0.2	0.26	-0.2	0.1	0.05	0.12	-0.19	-0.3	0.08	-0.05	0.02	-0.23	-0.02	-0.02
**Leaf density**	-0.28	0.2	-0.59	0.26	0.44	0.24	-0.08	0.08	-0.42	-0.18	0.12	0.01	-0.08	0	0.02	-0.07	0.09	-0.02
**Leaf K**	0.56	-0.52	-0.03	-0.21	0.18	-0.19	0.04	-0.09	0.01	-0.12	0.02	-0.23	-0.1	0.42	0.01	-0.06	0	0.01
**Leaf N**	0.73	0.13	0.14	-0.01	0.1	-0.25	0.17	-0.15	-0.09	-0.03	-0.21	0.17	-0.36	-0.16	0.09	-0.15	-0.02	0
**Leaf P**	0.56	-0.3	0.13	-0.25	0.41	-0.23	0.11	-0.13	-0.15	0.09	0.06	-0.23	0.24	-0.27	-0.02	0.05	0.02	0.01
**Leaf thickness**	-0.77	-0.1	-0.19	-0.33	-0.15	-0.07	0	-0.37	0.05	0.03	-0.13	-0.07	-0.07	-0.04	-0.17	-0.08	0.21	-0.09
**Leaf Vcmax**	0.75	-0.18	-0.38	-0.1	-0.04	-0.06	0.04	0.12	-0.04	0	-0.01	0.27	0.12	0.04	-0.36	-0.07	-0.04	0.01
**Root depth**	-0.19	0.13	-0.34	0.41	-0.34	-0.72	-0.04	0.1	-0.15	0.04	0.15	-0.05	0.07	0	0.06	-0.06	0.01	-0.02
**Seed dry mass**	0	0.46	0.01	0.35	0.46	-0.21	-0.41	-0.25	0.37	0	0	0.1	0.07	0.04	-0.02	-0.04	0	0.01
**Specific leaf area**	0.74	0.02	0.39	0.13	0.03	0.05	0.02	0.27	0.08	0.18	0.1	0.12	0.03	0.07	0.08	0	0.26	-0.1
**Stem diameter**	-0.22	0.02	-0.31	-0.67	0.09	-0.19	-0.09	0.42	0.25	-0.29	-0.06	0.03	0.02	-0.1	0.1	-0.04	0.05	0.04
**Stomatal conduct.**	0.52	0.15	-0.48	-0.21	-0.2	0.17	0.19	-0.39	0.14	-0.1	0.32	0.12	0.11	-0.01	0.17	-0.05	0.01	0.01
**Tree height**	0.01	0.77	0.23	-0.3	0.03	-0.03	0.02	0.1	0.06	0.06	0.4	-0.15	-0.24	-0.04	-0.17	-0.01	-0.04	-0.03
**Wood density**	-0.25	0.13	-0.42	0.44	0.21	0	0.61	0.13	0.32	0.06	-0.08	-0.1	-0.01	0	-0.04	0.02	0	-0.01

Supplementary Notes

Extended discussion of the two-trait clusters in Fig. 4

Intermediate to the two largest clusters in Fig. 4 (main text) is a constellation containing stem conduit diameter and stomatal conductance, demonstrating leaf/wood water regulation (Fig. 4, yellow). This constellation loads most strongly on PC 1, as moisture regulation, nutrient-use, and photosynthesis are closely interrelated. Nevertheless, conduit diameter, in particular, correlates moderately with traits indicative of light interception, notably leaf area ($\rho = 0.48$) and tree height ($\rho = 0.25$). Although the largest differences in stem conduit size are observed between angiosperms and gymnosperms, wider conduits confer greater conducting efficiency regardless of architecture[1]. Indeed, these patterns hold within clades as well, particularly when comparing conduit diameter to leaf area ($\rho = 0.43$ vs 0.38 for angiosperms vs. gymnosperms). The associations between water regulation and light interception highlights that leaf area and tree height induce important physiological and mechanical demands on organism-level water availability, which prevent against cavitation and desiccation.

Tree diameter and bark thickness also emerge as a distinct two-trait cluster. Recent research has shown that bark thickness is mainly driven by plant size (mainly stem diameter). This strong association likely reflects, not only bark accumulation as trees age, but also functional/metabolic needs as plants grow taller and increase in total leaf area[2,3]. From an ecological perspective, thick bark can be critical for defense against fire and pest damage (mainly a thick outer bark region), and for storage and photosynthate transport (mainly a thick inner bark region)[4,5]. Moreover, because older individuals are more likely to have been exposed to multiple disturbances across their lifetime, large-diameter trees in older forests can exhibit survivorship bias towards thick-bark individuals which were able to withstand historical stressors[3,6]. However, such relationships are strongly ecosystem-dependent, leading to weak overall relationships between climate, fire regimes, and bark thickness at the global scale, with stem diameter emerging as the strongest single predictor[2].

The final two-trait cluster is comprised of wood density and leaf density. Both traits are key indicators of “slow” life-history strategies in trees, correlating negatively with growth rate and

water transport, but positively with abiotic stress tolerance and resilience to disturbance[7]. Thick leaves and thick wood each protect against herbivory and pests while protecting against desiccation risk and mechanical damage[8–11]. Wood density has been identified as a particularly important multi-functional indicator of tree form and function, reflecting various aspects of tree hydraulics, pest resistance, decay rate, structural stability, tree size, growth rate, and tree mortality[9,12–15]. The production of dense wood and leaves is, however, more energetically costly, limiting growth rate but increasing life span[12,16]. The fact that wood and leaf density emerge as an independent trait constellation reinforces previous inference that these traits are aligned at one end of the slow-fast spectrum, and uniquely integrate multiple physiological and ecological pressures[7,12,16].

Exploration of invasive species in the dataset

We explored the level of invasiveness in our dataset by combining the GloNAF[17] and Kew[18] invasive species databases to identify whether each observation was occurring within that species' native or invasive range. The average proportion of invasive species observations was less than half a percent across all traits (0.3%), with leaf area having the highest proportion (0.814%), equating to 11 species out of 1352 species with a measurement taken at a location outside their native range. There was only one species with more than 5 measurements for a given trait taken out outside of its native range: *Castanea sativa* had 28, 20 and 22 measurements taken for wood density, leaf N and leaf P, making up significantly less than 0.5% of observations for each trait. Moreover, these locations where *C. sativa* is invasive are located in France, Spain, Germany, and the UK. And while these lie outside of its estimated original range, it has since been established in these regions for more than 1000 years[19]. Such results demonstrate the challenges exploring trait variation between invasive and native populations, in part due to widely different definitions of "invasive" across databases.

Supplementary References

1. Sperry, J. S., Hacke, U. G. & Pittermann, J. Size and function in conifer tracheids and angiosperm vessels. *Am. J. Bot.* **93**, 1490–1500 (2006).

2. Rosell, J. A. Bark thickness across the angiosperms: More than just fire. *New Phytol.* **211**, 90–102 (2016).

3. Williams, V. L., Witkowski, E. T. F. & Balkwill, K. Relationship between bark thickness and diameter at breast height for six tree species used medicinally in South Africa. *South African J. Bot.* **73**, 449–465 (2007).

4. Schafer, J. L., Breslow, B. P., Hohmann, M. G. & Hoffmann, W. A. Relative bark thickness is correlated with tree species distributions along a fire frequency gradient. *Fire Ecol.* **11**, 74–87 (2015).

5. Rosell, J. A. *et al.* Inner bark as a crucial tissue for non-structural carbohydrate storage across three tropical woody plant communities. *Plant Cell Environ.* **44**, 156–170 (2021).

6. Poorter, L., Mcneil, A., Hurtado, V. H., Prins, H. H. T. & Putz, F. E. Bark traits and life-history strategies of tropical dry- and moist forest trees. *Funct. Ecol.* **28**, 232–242 (2014).

7. Niinemets, Ü. Global-scale climatic controls of leaf dry mass per area, density, and thickness in trees and shrubs. *Ecology* **82**, 453–469 (2001).

8. Onoda, Y. *et al.* Global patterns of leaf mechanical properties. *Ecol. Lett.* **14**, 301–312 (2011).

9. Iida, Y. *et al.* Wood density explains architectural differentiation across 145 co-occurring tropical tree species. *Funct. Ecol.* **26**, 274–282 (2012).

10. Agrawal, A. a & Fishbein, M. Plant defense syndromes. *Ecology* **87**, S132-49 (2006).

11. Ruiz-Guerra, B., García, A., Velázquez-Rosas, N., Angulo, D. & Guevara, R. Plant-functional traits drive insect herbivory in a tropical rainforest tree community. *Perspect.*

Plant Ecol. Evol. Syst. **48**, (2021).

12. Chave, J. *et al.* Towards a worldwide wood economics spectrum. *Ecol. Lett.* **12**, 351–366 (2009).

13. Davi, H. *et al.* Distribution of endemic bark beetle attacks and their physiological consequences on Pinus halepensis. *For. Ecol. Manage.* **469**, (2020).

14. Wright, S. J. *et al.* Functional traits and the growth-mortality trade-off in tropical trees. *Ecology* **91**, 3664–3674 (2010).

15. King, D. A., Davies, S. J., Tan, S. & Noor, N. S. M. The role of wood density and stem support costs in the growth and mortality of tropical trees. *J. Ecol.* **94**, 670–680 (2006).

16. Reich, P. B. The world-wide 'fast-slow' plant economics spectrum: A traits manifesto. *J. Ecol.* **102**, 275–301 (2014).

17. van Kleunen, M. *et al.* The Global Naturalized Alien Flora (GloNAF) database. *Ecology* **100**, 1–2 (2019).

18. POWO. Plants of the World Online. Facilitated by the Royal Botanic Gardens, Kew (Published on the Internet). *2022* http://www.plantsoftheworldonline.org/.

19. Conedera, M., Krebs, P., Tinner, W., Pradella, M. & Torriani, D. The cultivation of Castanea sativa (Mill.) in Europe, from its origin to its diffusion on a continental scale. *Veg. Hist. Archaeobot.* **13**, 161–179 (2004).

20. Mill, R. R. *et al.* A new classification and linear sequence of extant gymnosperms. *Phytotaxa* **70**, 55–70 (2011).

21. BGCI. *GlobalTreeSearch online database (version 1.4). Botanic Gardens Conservation International*. (Available at https://tools.bgci.org/global_tree_search.php, 2020).

22. Karger, D. N., Conrad, O., Böhner, J., Kawohl, T. & Kreft, H. Data from: Climatologies at high resolution for the earth's land surface areas. *Dryad Digit. Repos.* (2018).

23. Karger, D. N. *et al.* Climatologies at high resolution for the earth's land surface areas. *Sci.*

Data **4**, 1–20 (2017).

24. Trabucco, A. & Zomer, R. Global Aridity Index and Potential Evapotranspiration (ET0) Climate Database v2. *Figshare. https://doi.org/10.6084/m9.figshare.7504448.v3* (2019).

25. Tuanmu, M. N. & Jetz, W. A global, remote sensing-based characterization of terrestrial habitat heterogeneity for biodiversity and ecosystem modelling. *Glob. Ecol. Biogeogr.* **24**, 1329–1339 (2015).

26. ESA Land Cover CCI project team & Defourny, P. ESA Land Cover Climate Change Initiative (Land_Cover_cci): Land Surface Seasonality Products. *Cent. Environ. Data Anal.* https://catalogue.ceda.ac.uk/uuid/7c114fc6e2884c1f (2017).

27. Obu, J. *et al.* Northern Hemisphere permafrost map based on TTOP modelling for 2000–2016 at 1 km2 scale. *Earth-Science Rev.* **193**, 299–316 (2019).

28. Fan, Y., Li, H. & Miguez-Macho, G. Global patterns of groundwater table depth. *Science (80-.).* **339**, 940–943 (2013).

29. Hengl, T. *et al.* SoilGrids250m: Global gridded soil information based on machine learning. *PLoS One* **12**, 1–40 (2017).

30. Díaz, S. *et al.* The global spectrum of plant form and function. *Nature* **529**, 1–17 (2015).

31. Joswig, J. S. *et al.* Climatic and soil factors explain the two-dimensional spectrum of global plant trait variation. *Nat. Ecol. Evol.* **6**, 24–27 (2022).
