# Supplementary material for: Global relationships in tree functional traits
Source: Nat Commun. 2022 Jun 8;13:3185. doi: 10.1038/s41467-022-30888-2 (PMC9177664; doi:10.1038/s41467-022-30888-2)
Supplement: Supplementary file 2 — Supplementary Data 1 [file 41467_2022_30888_MOESM2_ESM.pdf]

Supplementary Data References

1. Adriaenssens S. (2012). Dry deposition and canopy exchange for temperate tree species under high nitrogen deposition. PhD thesis, Ghent University, Ghent, Belgium, 209p.
2. Atkin OK (2015) Global variability in leaf respiration among plant functional types in relation to climate and leaf traits. New Phytologist DOI: 10.1111/nph.13253
3. Aubin, I., Messier, C., Gachet, S., Lawrence, K., McKenney, D., Arseneault, A., Bell, W., De Grandpré, L., Shipley, B., Ricard, J.P. and Munson, A.D., 2012. TOPIC-traits of plants in Canada. Natural Resources Canada, Canadian Forest Service, Sault Ste. Marie, Ontario. Online [URL] TOPIC website :http://cfs.cloud.nrcan.gc.ca/ctn/topic.php
4. Auger, S., Shipley, B. (2012). Interspecific and intraspecific trait variation along short environmental gradients in an old-growth temperate forest. Journal of Vegetation Science. DOI: 1111/j.1654-1103.2012.01473.x
5. Bahar, NHA, Ishida, FY, Weerasinghe, LK, Guerrieri, R, OSullivan, OS, Bloomfield, KJ, Asner, GP, Martin, RE, Lloyd, J, Malhi, Y, Phillips, OL, Meir, P, Salinas, N, Cosio, EG, Domingues, TF, Quesada, CA, Sinca, F, Escudero Vega, A, Zuloaga Ccorimanya, PP, del Aguila-Pasquel, J, Quispe Huaypar, K, Cuba Torres, I, Butrón Loayza, R, Pelaez Tapia, Y, Huaman Ovalle, J, Long, BM, Evans, JR, Atkin, OK (2017) Leaf-level photosynthetic capacity in lowland Amazonian and high-elevation Andean tropical moist forests of Peru. New Phytologist 214, 1002-1018.
6. Baraloto, C., C. E. T. Paine, L. Poorter, J. Beauchene, D. Bonal, A.-M. Domenach, B. Herault, S. Patino, J.-C. Roggy, and J. Chave. 2010. Decoupled leaf and stem economics in rainforest trees. Ecology Letters 13:1338-1347
7. Baruch, Z. & Goldstein, G. 1999. Leaf construction cost, nutrient concentration, and net CO2 assimilation of native and invasive species in Hawaii. Oecologia 121: 183-192

8. Blonder, B., Baldwin, B., Enquist, B.J., Robichaux, R.H. (2016) Variation and macroevolution in leaf functional traits in the Hawaiian silversword alliance (Asteraceae). Journal of Ecology 104:219-228 DOI: 10.1111/1365-2745.12497

9. Blonder, B., Buzzard, B., Sloat, L., Simova, I., Lipson, R., Boyle, B., Enquist, B. (2012) The shrinkage effect biases estimates of paleoclimate. American Journal of Botany. 99.11 1756-1763

10. Bond-Lamberty, B., C. Wang, and S. T. Gower (2002), Above- and belowground biomass and sapwood area allometric equations for six boreal tree species of northern Manitoba, Can. J. For. Res., 32(8), 1441-1450.

11. Bond-Lamberty, B., C. Wang, and S. T. Gower (2002), Leaf area dynamics of a boreal black spruce fire chronosequence, Tree Physiol., 22(14), 993-1001.

12. Bond-Lamberty, B., C. Wang, and S.T. Gower (2004), Net primary production and net ecosystem production of a boreal black spruce fire chronosequence, Global Change Biol., 10(4), 473-487.

13. Brendan Choat, Steven Jansen, Tim J. Brodribb, Herve Cochard, Sylvain Delzon, Radika Bhaskar, Sandra J. Bucci, Taylor S. Feild, Sean M. Gleason, Uwe G. Hacke, Anna L. Jacobsen, Frederic Lens, Hafiz Maherali, Jordi Martinez-Vilalta, Stefan Mayr, Maurizio Mencuccini, Patrick J. Mitchell, Andrea Nardini, Jarmila Pittermann, R. Brandon Pratt, John S. Sperry, Mark Westoby, Ian J. Wright & Amy E. Zanne (2012) Global convergence in the vulnerability of forests to drought. Nature 491:752-755 doi:10.1038/nature11688

14. Brown, K.A., S.E. Johnson, K. Parks, S.M. Holmes, T. Ivoandry, N.K. Abram, K.E. Delmore, R. Ludovic, H.E. Andriamaharoa, T.M. Wyman, P.C. Wright (2013) Use of provisioning ecosystem services drives loss of functional traits across land use intensification gradients in tropical forests in Madagascar. Biological Conservation 161: 118-127

15. Buchanan, S., Isaac, M.E., Van den Meersche, K. et al. Agroforest Syst (2018). https://doi.org/10.1007/s10457-018-0239-1

16. Burrascano S, Copiz R, Del Vico E, Fagiani S, Giarrizzo E, Mei M, Mortelliti A, Sabatini FM, Blasi C (2015) Wild boar rooting intensity determines shifts in understorey composition and functional traits. Community ecology 16(2) 244-253 DOI: 10.1556/168.2015.16.2.12

17. Butterfield, B.J. and J.M. Briggs. 2011. Regeneration niche differentiates functional strategies of desert woody plant species. Oecologia, 165:477-487.

18. Campetella, G; Botta-Dukát, Z; Wellstein, C; Canullo, R; Gatto, S; Chelli, S; Mucina, L; Bartha, S (2011): Patterns of plant trait-environment relationships along a forest succession chronosequence. Agriculture, Ecosystems & Environment, 145(1), 38-48. doi:10.1016/j.agee.2011.06.025

19. Carswell, F. E., Meir, P., Wandelli, E. V., Bonates, L. C. M., Kruijt, B., Barbosa, E. M., Nobre, A. D. & Jarvis, P. G. 2000 Photosynthetic capacity in a central Amazonian rain forest. Tree physiology. 20, 3, p. 179-186 8 p.

20. Catford, J. A., Morris, W. K., Vesk, P. A., Gippel, C. J. & Downes, B. J. (2014) Species and environmental characteristics point to flow regulation and drought as drivers of riparian plant invasion. Diversity and Distributions, 20, 1084-1096. http://dx.doi.org/10.1111/ddi.12225

21. Cavender-Bares, J., A. Keen, and B. Miles. 2006. Phylogenetic structure of floridian plant communities depends on taxonomic and spatial scale. Ecology 87:S109-S122.

22. Chacón-Madrigal, E., Wanek, W., Hietz, P., & S. Dullinger. 2018. Traits indicating a conservative resource strategy are weakly related to narrow range size in a group of neotropical trees. Perspectives in Plant Ecology, Evolution, and Systematics, https://doi.org/10.1016/j.ppees.2018.01.003

23. Cornelissen, J. H. C. 1996. An experimental comparison of leaf decomposition rates in a wide range of temperate plant species and types. Journal of Ecology 84:573-582.

24. Cornelissen, J. H. C., B. Cerabolini, P. Castro-Diez, P. Villar-Salvador, G. Montserrat-Marti, J. P. Puyravaud, M. Maestro, M. J. A. Werger, and R. Aerts. 2003. Functional traits

of woody plants: correspondence of species rankings between field adults and laboratory-grown seedlings? Journal of Vegetation Science 14:311-322.

25. Cornelissen, J. H. C., H. M. Quested, D. Gwynn-Jones, R. S. P. Van Logtestijn, M. A. H. De Beus, A. Kondratchuk, T. V. Callaghan, and R. Aerts. 2004. Leaf digestibility and litter decomposability are related in a wide range of subarctic plant species and types. Functional Ecology 18:779-786.

26. Cornwell, W. K., R. Bhaskar, L. Sack, S. Cordell, and C. K. Lunch. 2007. Adjustment of structure and function of Hawaiian Metrosideros polymorpha at high vs. low precipitation. Functional Ecology 21:1063-1071.

27. Craine, J. M., A. J. Elmore, M. P. M. Aidar, M. Bustamante, T. E. Dawson, E. A. Hobbie, A. Kahmen, M. C. Mack, K. K. McLauchlan, A. Michelsen, G. B. Nardoto, L. H. Pardo, J. Penuelas, P. B. Reich, E. A. G. Schuur, W. D. Stock, P. H. Templer, R. A. Virginia, J. M. Welker, and I. J. Wright. 2009. Global patterns of foliar nitrogen isotopes and their relationships with climate, mycorrhizal fungi, foliar nutrient concentrations, and nitrogen availability. New Phytologist 183:980-992.

28. Craven, D., D. Braden, M. S. Ashton, G. P. Berlyn, M. Wishnie, and D. Dent. 2007. Between and within-site comparisons of structural and physiological characteristics and foliar nutrient content of 14 tree species at a wet, fertile site and a dry, infertile site in Panama. Forest Ecology and Management 238:335-346.

29. Díaz, S., J. G. Hodgson, K. Thompson, M. Cabido, J. H. C. Cornelissen, A. Jalili, G. Montserrat-Martí, J. P. Grime, F. Zarrinkamar, Y. Asri, S. R. Band, S. Basconcelo, P. Castro-Díez, G. Funes, B. Hamzehee, M. Khoshnevi, N. Pérez-Harguindeguy, M. C. Pérez-Rontomé, F. A. Shirvany, F. Vendramini, S. Yazdani, R. Abbas-Azimi, A. Bogaard, S. Boustani, M. Charles, M. Dehghan, L. de Torres-Espuny, V. Falczuk, J. Guerrero-Campo, A. Hynd, G. Jones, E. Kowsary, F. Kazemi-Saeed, M. Maestro-Martínez, A. Romo-Díez, S. Shaw, B. Siavash, P. Villar-Salvador, and M. R. Zak. 2004. The plant traits that drive ecosystems: Evidence from three continents. Journal of Vegetation Science 15:295-304.

30. Dahlin KM, Asner GP & CB Field (2013) Environmental and community controls on plant canopy chemistry in a Mediterranean-type ecosystem. Proceedings of the National Academy of Sciences USA. 110(17): 6895-6900

31. Dawson, S. K., Warton, D. I., Kingsford, R. T., Berney, P. , Keith, D. A., Catford, J. A. and Mori, A. (2017), Plant traits of propagule banks and standing vegetation reveal flooding alleviates impacts of agriculture on wetland restoration. J Appl Ecol, 54: 1907-1918. doi:10.1111/1365-2664.12922

32. de Araujo, A.C., J. P. H. B. Ometto, A. J. Dolman, B. Kruijt, M. J. Waterloo and J. R. Ehleringer. 2011. LBA-ECO CD-02 C and N Isotopes in Leaves and Atmospheric CO2, Amazonas, Brazil. Data set. Available on-line [http://daac.ornl.gov] from Oak Ridge National Laboratory Distributed Active Archive Center, Oak Ridge, Tennessee, U.S.A.

33. de Araujo, A.C., J.P.H.B. Ometto, A.J. Dolman, B. Kruijt, M.J. Waterloo and J.R. Ehleringer. 2012. LBA-ECO CD-02 C and N Isotopes in Leaves and Atmospheric CO2, Amazonas, Brazil. Data set. Available on-line [http://daac.ornl.gov] from Oak Ridge National Laboratory Distributed Active Archive Center, Oak Ridge, Tennessee, U.S.A. http://dx.doi.org/10.3334/ORNLDAAC/1097

34. Domingues TF, Meir P, Feldpausch TR, et al. (2010) Co-limitation of photosynthetic capacity by nitrogen and phosphorus in West Africa woodlands. Plant, Cell & Environment (33): 959-980.

35. Falster DS, Remko A. Duursma, Masae I. Ishihara, Diego R. Barneche, Richard G. FitzJohn, Angelica Våhammar, Masahiro Aiba, Makoto Ando, Niels Anten, Michael J. Aspinwall, Jennifer L. Baltzer, Christopher Baraloto, Michael Battaglia, John J. Battles, Ben Bond-Lamberty, Michiel van Breugel, James Camac, Yves Claveau, Lluís Coll, Masako Dannoura, Sylvain Delagrange, Jean-Christophe Domec, Farrah Fatemi, Wang Feng, Veronica Gargaglione, Yoshiaki Goto, Akio Hagihara, Jefferson S. Hall, Steve Hamilton, Degi Harja, Tsutom Hiura, Robert Holdaway, Lindsay B. Hutley, Tomoaki Ichie, Eric J. Jokela, Anu Kantola, Jeff W. G. Kelly, Tanaka Kenzo, David King, Brian D. Kloeppel, Takashi Kohyama, Akira Komiyama, Jean-Paul Laclau, Christopher H. Lusk, Douglas A. Maguire, Guerric le Maire, Annikki Mäkelä, Lars Markesteijn, John Marshall,

Katherine McCulloh, Itsuo Miyata, Karel Mokany, Shigeta Mori, Randall W. Myster, Masahiro Nagano, Shawna L. Naidu, Yann Nouvellon, Anthony P. OGrady, Kevin L. OHara, Toshiyuki Ohtsuka, Noriyuki Osada, Olusegun O. Osunkoya, Pablo Luis Peri, Any Mary Petritan, Lourens Poorter, Angelika Portsmuth, Catherine Potvin, Johannes Ransijn, Douglas Reid, Sabina C. Ribeiro, Scott D. Roberts, Rolando Rodríguez, Angela Saldaña-Acosta, Ignacio Santa-Regina, Kaichiro Sasa, N. Galia Selaya, Stephen C. Sillett, Frank Sterck, Kentaro Takagi, Takeshi Tange, Hiroyuki Tanouchi, David Tissue, Toru Umehara, Hajime Utsugi, Matthew A. Vadeboncoeur, Fernando Valladares, Petteri Vanninen, Jian R. Wang, Elizabeth Wenk, Richard Williams, Fabiano de Aquino Ximenes, Atsushi Yamaba, Toshihiro Yamada, Takuo Yamakura, Ruth D. Yanai, and Robert A. York. 2015. BAAD: a biomass and allometry database for woody plants. Ecology 96:1445.http://dx.doi.org/10.1890/14-1889.1

36. Fan Y, Gonzalo Miguez-Macho, Esteban G. Jobbágy, Robert B. Jackson, Carlos Otero-Casal (2017) Hydrologic regulation of plant rooting depth. Proceedings of the National Academy of Sciences 114 (40) 10572-10577; DOI: 10.1073/pnas.1712381114

37. Fonseca, C. R., J. M. Overton, B. Collins, and M. Westoby. 2000. Shifts in trait-combinations along rainfall and phosphorus gradients. Journal of Ecology 88:964-977.

38. Forgiarini, C., Souza, A.F., Longhi, S.J., Oliveira, J.M., 2015. In the lack of extreme pioneers: trait relationships and ecological strategies of 66 subtropical tree species. J. Plant Ecol. 8, 359-367. doi:10.1093/jpe/rtu028

39. Freschet, G. T., J. H. C. Cornelissen, R. S. P. van Logtestijn, and R. Aerts. 2010. Evidence of the ‘plant economics spectrum’ in a subarctic flora. Journal of Ecology 98:362-373.

40. Fyllas, N. M., S. Patino, T. R. Baker, G. Bielefeld Nardoto, L. A. Martinelli, C. A. Quesada, R. Paiva, M. Schwarz, V. Horna, L. M. Mercado, A. Santos, L. Arroyo, E. M. Jimenez, F. J. Luizao, D. A. Neill, N. Silva, A. Prieto, A. Rudas, M. Silviera, I. C. G. Vieira, G. Lopez-Gonzale, and J. Lloyd. 2009. Basin-wide variations in foliar properties of Amazonian forest: phylogeny, soils and climate Biogeosciences 6:2677-2708.

41. Garnier, E., S. Lavorel, P. Ansquer, H. Castro, P. Cruz, J. Dolezal, O. Eriksson, C. Fortunel, H. Freitas, C. Golodets, K. Grigulis, C. Jouany, E. Kazakou, J. Kigel, M. Kleyer, V.

Lehsten, J. Leps, T. Meier, R. Pakeman, M. Papadimitriou, V. P. Papanastasis, H. Quested, F. Quetier, M. Robson, C. Roumet, G. Rusch, C. Skarpe, M. Sternberg, J.-P. Theau, A. Thebault, D. Vile, and M. P. Zarovali. 2007. Assessing the effects of land-use change on plant traits, communities and ecosystem functioning in grasslands: A standardized methodology and lessons from an application to 11 European sites. Annals of Botany 99:967-985.

42. Givnish T.J., R.A. Montgomery and G. Goldstein. 2004. Adaptive radiation of photosynthetic physiology in the Hawaiian lobeliads: light regimes, static light responses, and whole-plant compensation points. American Journal of Botany 91: 228-246

43. Gonzalez-Akre, E., McShea, W., Bourg, N., Anderson-Teixeira, K. 2015. Leaf traits data (SLA) for 56 woody species at the Smithsonian Conservation Biology Institute-ForestGEO Forest Dynamic Plot. Front Royal, Virginia. USA. [Data set]. Version 1.0.(www.try-db.org)

44. Guerin G.R., Wen H. & Lowe A.J. (2012) Leaf morphology shift linked to climate change. Biology Letters 8, 882-886.

45. Gutiérrez AG, & Huth A (2012) Successional stages of primary temperate rainforests of Chiloé Island, Chile. Perspectives in plant ecology, systematics and evolution. 14: 243-256

46. Han, W. X., J. Y. Fang, D. L. Guo, and Y. Zhang. 2005. Leaf nitrogen and phosphorus stoichiometry across 753 terrestrial plant species in China. New Phytologist 168:377-385.

47. Hao, G. Y., L. Sack, A. Y. Wang, K. F. Cao, and G. Goldstein. 2010. Differentiation of leaf water flux and drought tolerance traits in hemiepiphytic and non-hemiepiphytic Ficus tree species. Functional Ecology 24:731-740.

48. Hietz, P., Rosner, S., Hietz-Seifert, U. & Wright, S.J. (2017). Wood traits related to size and life history of trees in a Panamanian rainforest. New Phytol., 213, 170-180

49. HIGUCHI, P.; SILVA, A.C. Araucaria Forest Database. 2013

50. Hoof, J., L. Sack, D. T. Webb, and E. T. Nilsen. 2008. Contrasting structure and function of pubescent and glabrous varieties of Hawaiian Metrosideros polymorpha (Myrtaceae) at high elevation. Biotropica 40:113-118.

51. Iversen CM, McCormack ML, Powell AS, Blackwood CB, Freschet GT, Kattge J, Roumet C, Stover DB, Soudzilovskaia NA, Valverde-Barrantes OJ, van Bodegom PM, Violle C (2017) A global Fine-Root Ecology Database to address belowground challenges in plant ecology. New Phytologist. doi:10.1111/nph.14486.

52. Jennifer S. Powers and Peter Tiffin 2012 Plant functional type classifications in tropical dry forests in Costa Rica: leaf habit versus taxonomic approaches. Functional Ecology 2010, 24, 927-936 doi: 10.1111/j.1365-2435.2010.01701.x

53. Joseph, G.S., Seymour, C.L., Cumming, G.S., Cumming, D.H.M., & Mahlangu, Z. 2014. Termite mounds increase functional diversity of woody plants in African savannas. Ecosystems 17: 808-819.

54. Kattge, J., W. Knorr, T. Raddatz, and C. Wirth. 2009. Quantifying photosynthetic capacity and its relationship to leaf nitrogen content for global-scale terrestrial biosphere models. Global Change Biology 15:976-991.

55. Kichenin et al. 2013. Contrasting effects of plant inter- and intraspecific variation on community-level trait measures along an environmental gradient. Functional Ecology, in press.

56. Kleyer, M., R. M. Bekker, I. C. Knevel, J. P. Bakker, K. Thompson, M. Sonnenschein, P. Poschlod, J. M. van Groenendael, L. Klimes, J. Klimesova, S. Klotz, G. M. Rusch, Hermy, M. , D. Adriaens, G. Boedeltje, B. Bossuyt, A. Dannemann, P. Endels, L. Götzenberger, J. G. Hodgson, A.-K. Jackel, I. Kühn, D. Kunzmann, W. A. Ozinga, C. Römermann, M. Stadler, J. Schlegelmilch, H. J. Steendam, O. Tackenberg, B. Wilmann, J. H. C. Cornelissen, O. Eriksson, E. Garnier, and B. Peco. 2008. The LEDA Traitbase: a database of life-history traits of the Northwest European flora. Journal of Ecology 96:1266-1274.

57. Knauer et al. (2017) Towards physiologically meaningful water-use efficiency estimates from eddy covariance data. Global Change Biology, DOI: 10.1111/gcb.13893

58. Kraft, N. J. B., R. Valencia, and D. Ackerly. 2008. Functional traits and niche-based tree community assembly in an Amazonian forest. Science 322:580-582.

59. Kurokawa, H. and T. Nakashizuka. 2008. Leaf herbivory and decomposability in a Malaysian tropical rain forest. Ecology 89:2645-2656.

60. Laughlin, D. C., J. J. Leppert, M. M. Moore, and C. H. Sieg. 2010. A multi-trait test of the leaf-height-seed plant strategy scheme with 133 species from a pine forest flora. Functional Ecology 24:493-501.

61. Laughlin, D.C., P.Z. Fulé, D.W. Huffman, J. Crouse, and E. Laliberte. 2011. Climatic constraints on trait-based forest assembly. Journal of Ecology 99:1489-1499.

62. Lhotsky B., Anikó Csecserits, Bence Kovács, Zoltán Botta-Dukát: New plant trait records of the Hungarian flora

63. Li, R., Zhu, S., Chen, H. Y. H., John, R., Zhou, G., Zhang, D., Zhang, Q. and Ye, Q. (2015), Are functional traits a good predictor of global change impacts on tree species abundance dynamics in a subtropical forest?. Ecol Lett, 18: 1181-1189. doi:10.1111/ele.12497

64. Lin Y-S, Medlyn BE, Duursma RA, Prentice IC, Wang H, Baig S, Eamus D, De Dios VR, Mitchell P, Ellsworth DS, De Beeck MO, Wallin G, Uddling J, Tarvainen L, Linderson M-L, Cernusak LA, Nippert JB, Ocheltree TW, Tissue DT, Martin-StPaul NK, Rogers A, Warren JM, De Angelis P, Hikosaka K, Han Q, Onoda Y, Gimeno TE, Barton CVM, Bennie J, Bonal D, Bosc A, Löw M, Macinins-Ng C, Rey A, Rowland L, Setterfield SA, Tausz-Posch S, Zaragoza-Castells J, Broadmeadow MSJ, Drake JE, Freeman M, Ghannoum O, Hutley LB, Kelly JW, Kikuzawa K, Kolari P, Koyama K, Limousin J-M, Meir P, Da Costa ACL, Mikkelsen TN, Salinas N, Sun W, Wingate L, (2015) Optimal stomatal behaviour around the world. Nature Climate Change 5(5): 459-464 DOI: 10.1038/NCLIMATE2550

65. Lukeš, P., Stenberg, P., Rautiainen, M., Mõttus, M., Vanhatalo, K.M. Optical properties of leaves and needles for boreal tree species in Europe (2013) Remote Sensing Letters, 4 (7), pp. 667-676

66. Lusk, C. H., Kaneko, T., Grierson, E. and Clearwater, M. (2013) Correlates of tree species sorting along a temperature gradient in New Zealand rain forests: seedling functional traits, growth and shade tolerance. Journal of Ecology, 101: 1531-1541.

67. Maire V, Ian J. Wright, I. Colin Prentice, Niels H. Batjes, Radika Bhaskar, Peter M. van Bodegom, Will K. Cornwell, David Ellsworth, Ülo Niinemets, Alejandro Ordoñez, Peter B. Reich, Louis S. Santiago (2015). Global soil and climate effects on leaf photosynthetic traits and rates. Global Ecology and Biogeography 24(6): 706-717. Maire V, Wright IJ, Prentice IC, Batjes NH, Bhaskar R, van Bodegom PM, Cornwell WK, Ellsworth D, Niinemets Ü, Ordoñez A, Reich PB, Santiago LS (2015) Data from: Global effects of soil and climate on leaf photosynthetic traits and rates. Dryad Digital Repository. http://dx.doi.org/10.5061/dryad.j42m7

68. Martinez-Garza, C., F. Bongers, and L. Poorter. 2013a. Are functional traits good predictors of species performance in restoration plantings in tropical abandoned pastures? Forest Ecology and Management 303:35-45

69. Medlyn, B. E., F.-W. Badeck, D. G. G. De Pury, C. V. M. Barton, M. Broadmeadow, R. Ceulemans, P. De Angelis, M. Forstreuter, M. E. Jach, S. Kellomäki, E. Laitat, M. Marek, S. Philippot, A. Rey, J. Strassemeyer, K. Laitinen, R. Liozon, B. Portier, P. Roberntz, K. Wang, and P. G. Jarvis. 1999. Effects of elevated CO2 on photosynthesis in European forest species: a meta-analysis of model parameters. Plant, Cell and Environment 22:1475-1495.

70. Meir, P. & Levy, P. E. 2007 Photosynthetic parameters from two contrasting woody vegetation types in West Africa. Plant Ecology. 192, 2, p. 277-287 11 p.

71. Meir, P., Kruijt, B., Broadmeadow, M., Kull, O., Carswell, F. & Nobre, A. 2002 Acclimation of photosynthetic capacity to irradiance in tree canopies in relation to leaf nitrogen concentration and leaf mass per unit area. Plant, Cell and Environment. 25, 3, p. 343-357 15 p.

72. Messier, J., B. J. McGill, and M. J. Lechowicz. 2010. How do traits vary across ecological scales? A case for trait-based ecology. Ecology Letters 13:838-848.

73. Michaletz, S.T., and E.A. Johnson. 2006. A heat transfer model of crown scorch in forest fires. Canadian Journal of Forest Research 36(11): 2839-2851

74. Milla & Reich 2011 Annals of Botany 107: 455-465, 2011.

75. Miller JED, Ives AR, Harrison SP, Damschen EI (2018) Early and late flowering guilds respond differently to landscape spatial structure. J Ecol. 106:1033-1045. https://doi.org/10.1111/1365-2745.12849

76. Mori, A. S., Shiono, T., Haraguchi, T. F., Ota, A. T., Koide, D., Ohgue, T., Kitagawa, R., Maeshiro, R., Aung, T. T., Nakamori, T., Hagiwara, Y., Matsuoka, S., Ikeda, A., Hishi, T., Hobara, S., Mizumachi, E., Frisch, A., Thor, G., Fujii, S., Osono, T. and Gustafsson, L. (2015), Functional redundancy of multiple forest taxa along an elevational gradient: predicting the consequences of non-random species loss. J. Biogeogr., 42: 1383-1396. doi:10.1111/jbi.12514

77. Muller, S. C., G. E. Overbeck, J. Pfadenhauer, and V. D. Pillar. 2007. Plant functional types of woody species related to fire disturbance in forest-grassland ecotones. Plant Ecology 189:1-14.

78. Niinemets, U. 2001. Global-scale climatic controls of leaf dry mass per area, density, and thickness in trees and shrubs. Ecology 82:453-469.

79. Nolan, R.H., Fairweather, K.A., Tarin, T., Santini, N.S., Cleverly, J., Faux, R. & Eamus, D. 2017, Divergence in plant water-use strategies in semiarid woody species, Functional Plant Biology, vol. 44, no. 11, pp. 1134-1146

80. Nolan, R.H., Tarin, T., Fairweather, K.A., Cleverly, J. and Eamus, D., 2017. Variation in photosynthetic traits related to access to water in semiarid Australian woody species. Funct. Plant Biol., 44(11): 1087-1097

81. Ogaya, R. and J. Penuelas. 2003. Comparative field study of Quercus ilex and Phillyrea latifolia: photosynthetic response to experimental drought conditions. Environmental and Experimental Botany 50:137-148.

82. Onstein RE, Richard J. Carter, Yaowu Xing, H. Peter LinderInstitute (2014) Diversification rate shifts in the Cape Floristic Region: The right traits in the right place at the right time. Perspectives in Plant Ecology, Evolution and Systematics 16(6) 331-340 DOI:10.1016/j.ppees.2014.08.002

83. Ordonez, J. C., P. M. van Bodegom, J. P. M. Witte, R. P. Bartholomeus, J. R. van Hal, and R. Aerts. 2010. Plant Strategies in Relation to Resource Supply in Mesic to Wet Environments: Does Theory Mirror Nature? American Naturalist 175:225-239.

84. Penuelas, J., J. Sardans, J. Llusia, S. Owen, J. Carnicer, T. W. Giambelluca, E. L. Rezende, M. Waite, and Ü. Niinemets. 2010. Faster returns on "leaf economics" and different biogeochemical niche in invasive compared with native plant species. Global Change Biology 16:2171-2185.

85. Petter G, Wagner K, Zotz G, Cabral JS, Wanek W, Sanchez Delgado EJ, Kreft H. 2016. Distribution of functional leaf traits of vascular epiphytes: vertical trends, intra- and interspecific trait variability, and phylogenetic signals. Functional Ecology, 30: 188-198.

86. Pierce S., Brusa G., Vagge I., Cerabolini B.E.L. (2013) Allocating CSR plant functional types: the use of leaf economics and size traits to classify woody and herbaceous vascular plants. Functional Ecology, 27(4): 1002-1010

87. Pierce S., Luzzaro A., Caccianiga M., Ceriani R.M. & Cerabolini B. 2007. Disturbance is the principal-scale filter determining niche differentiation, coexistence and biodiversity in an alpine community. Journal of Ecology 95: 698-706.

88. Pillar, V. D. and E. E. Sosinski. 2003. An improved method for searching plant functional types by numerical analysis. Journal of Vegetation Science 14:323-332.

89. Prentice, I.C., Meng, T., Wang, H., Harrison, S.P., Ni, J., Wang, G., 2011. Evidence for a universal scaling relationship of leaf CO2 drawdown along a moisture gradient. New Phytologist 190: 169-180

90. Preston, K. A., W. K. Cornwell, and J. L. DeNoyer. 2006. Wood density and vessel traits as distinct correlates of ecological strategy in 51 California coast range angiosperms. New Phytologist 170:807-818.

91. Price, C.A. and B.J. Enquist. Scaling of mass and morphology in Dicotyledonous leaves: an extension of the WBE model. (2007) Ecology 88(5): 1132-1141.

92. Quero, J. L., R. Villar, T. Maranon, R. Zamora, D. Vega, and L. Sack. 2008. Relating leaf photosynthetic rate to whole-plant growth: drought and shade effects on seedlings of four Quercus species. Functional Plant Biology 35:725-737.

93. Quested, H. M., J. H. C. Cornelissen, M. C. Press, T. V. Callaghan, R. Aerts, F. Trosien, P. Riemann, D. Gwynn-Jones, A. Kondratchuk, and S. E. Jonasson. 2003. Decomposition of sub-arctic plants with differing nitrogen economies: A functional role for hemiparasites. Ecology 84:3209-3221.

94. Reich, P. B., J. Oleksyn, and I. J. Wright. 2009. Leaf phosphorus influences the photosynthesis-nitrogen relation: a cross-biome analysis of 314 species. Oecologia 160:207-212.

95. Reich, P. B., M. G. Tjoelker, K. S. Pregitzer, I. J. Wright, J. Oleksyn, and J. L. Machado. 2008. Scaling of respiration to nitrogen in leaves, stems and roots of higher land plants. Ecology Letters 11:793-801.

96. Rodrigues, A.V.; Bones, F.L.V.; Schneiders, A.; Oliveira, L.Z.; Vibrans, A.C.; Gasper, A.L. Plant Trait Dataset for Tree-Like Growth Forms Species of the Subtropical Atlantic Rain Forest in Brazil. Data 2018, 3, 16.

97. Rolo V., López-Díaz M. L. and Moreno G. (2012) Shrubs affect soil nutrients availability with contrasting consequences for pasture understory and tree overstory production and nutrient status in Mediterranean grazed open woodlands. Nutrient Cycling in Agroecosystems, 1-14

98. Rolo, V., Olivier, P. and van Aarde, R. (2016) Seeded pioneer die-offs reduce the functional trait space of new-growth coastal dune forests. Forest Ecology and Management, 377, 26-35.

99. Rosell, J. A. (2016), Bark thickness across the angiosperms: more than just fire. New Phytol, 211: 90-102. doi:10.1111/nph.13889

100. Rosell, J. A., Olson, M. E., Anfodillo, T. and Martínez-Méndez, N. (2017), Exploring the bark thickness-stem diameter relationship: clues from lianas, successive cambia, monocots and gymnosperms. New Phytol, 215: 569-581. doi:10.1111/nph.14628

101. Sack, L. 2004. Responses of temperate woody seedlings to shade and drought: do trade-offs limit potential niche differentiation? Oikos 107:110-127.

102. Sack, L., P. D. Cowan, N. Jaikumar, and N. M. Holbrook. 2003. The 'hydrology' of leaves: co-ordination of structure and function in temperate woody species. Plant Cell and Environment 26:1343-1356.

103. Scalon, M.C., Haridasan, M. & Franco, A.C. Plant Soil (2017). https://doi.org/10.1007/s11104-017-3437-0

104. Scherer-Lorenzen, M., Schulze, E.-D., Don, A., Schumacher, J. & Weller, E. (2007) Exploring the functional significance of forest diversity: A new long-term experiment with temperate tree species (BIOTREE). Perspectives in Plant Ecology, Evolution and Systematics, 9, 53-70.

105. Schurr,F.M., Midgley,G.F., Rebelo,A.G., Reeves,G., Poschlod,P. & Higgins,S.I. (2007) Global Ecology and Biogeography, 16, 449-459.

106. Seymour, C.L., Milewski, A.V., Mills, A.J., Joseph, G.S., Cumming, G.S., Cumming, D.H.M., & Mahlangu, Z. 2014. Do the large termite mounds of Macrotermes concentrate micronutrients in addition to macronutrients in nutrient-poor African savannas? Soil Biology and Biochemistry 68: 95-105.

107. Shiodera, S., J. S. Rahajoe, and T. Kohyama. 2008. Variation in longevity and traits of leaves among co-occurring understorey plants in a tropical montane forest. Journal of Tropical Ecology 24:121-133.

108. Shipley, B. and T. T. Vu. 2002. Dry matter content as a measure of dry matter concentration in plants and their parts. New Phytologist 153:359-364.

109. Smith, N. G. and Dukes, J. S. (2017), LCE: leaf carbon exchange data set for tropical, temperate, and boreal species of North and Central America. Ecology, 98: 2978. doi:10.1002/ecy.1992

110. Souza K, Higuchi P, Silva Ac, Schimalski Mb, Loebens R, Buzzi Junior F, Souza Cc, Rodrigues Junior Lc, Walter Ff, Missio Ff, Dalla Rosa A. (2017) Partição de nicho por grupos funcionais de espécies arbóreas em uma floresta subtropical. Rodriguésia [online] vol.68, n.4, pp.1165-1175. ISSN 0370-6583. http://dx.doi.org/10.1590/2175-7860201768401

111. Spasojevic, M. J., Turner, B. L., and Myers, J. A. (2016) When does intraspecific trait variation contribute to functional beta?diversity? J Ecol, 104: 487-496. doi:10.1111/1365-2745.12518

112. Swaine, E. K. 2007. Ecological and evolutionary drivers of plant community assembly in a Bornean rain forest. PhD Thesis, University of Aberdeen, Aberdeen.

113. Takkis, K. 2014. Changes in plant species richness and population performance in response to habitat loss and fragmentation. Dissertationes Biologicae Universitatis Tartuensis 255, 2014-04-07. Available from: http://hdl.handle.net/10062/39546

114. Tamir Klein, Giovanni Di Matteo, Eyal Rotenberg, Shabtai Cohen, Dan Yakir (2012) Differential ecophysiological response of a major Mediterranean pine species across a climatic gradient. Tree Physiology 33 (1): 26-36. doi: 10.1093/treephys/tps116

115. Thuiller W - Traits of European Alpine Flora - Wilfried Thuiller - OriginAlps Project - Centre National de la Recherche Scientifique

116. Tomas F. Domingues, Luiz A. Martinelli, James R. Ehleringer (2007) Ecophysiological traits of plant functional groups in forest and pasture ecosystems from eastern Amazonia, Brazil. Plant Ecol (2007) 193:101-112 DOI 10.1007/s11258-006-9251-z

117. van de Weg MJ, Meir P,Grace J, Atkin O (2009) Altitudinal variation in leaf mass per unit area, leaf tissue density and foliar nitrogen and phosphorus content along the Amazon-Andes gradient in Peru, Plant Ecology& Diversity, 2: 3, 243-254

118. van de Weg MJ, Patrick Meir John Grace, Guilmair Damian Ramos (2011) Photosynthetic parameters, dark respiration and leaf traits in the canopy of a Peruvian tropical montane cloud forest Oecologia DOI 10.1007/s00442-011-2068-z

119. van der Plas, F. & Olff, H. (2014) Mesoherbivores affect grasshopper communities in a megaherbivore-dominated South African savannah. Oecologia 175: 639. doi:10.1007/s00442-014-2920-z

120. Vergutz, L., S. Manzoni, A. Porporato, R.F. Novais, and R.B. Jackson. 2012. A Global Database of Carbon and Nutrient Concentrations of Green and Senesced Leaves. Data set. Available on-line [http://daac.ornl.gov] from Oak Ridge National Laboratory Distributed Active Archive Center, Oak Ridge, Tennessee, U.S.A. http://dx.doi.org/10.3334/ORNLDAAC/1106

121. Walker, A.P. 2014. A Global Data Set of Leaf Photosynthetic Rates, Leaf N and P, and Specific Leaf Area. Data set. Available on-line [http://daac.ornl.gov] from Oak Ridge National Laboratory Distributed Active Archive Center, Oak Ridge, Tennessee, USA. http://dx.doi.org/10.3334/ORNLDAAC/1224

122. Wang, Han; Harrison, Sandy P; Prentice, Iain Colin; Yang, Yanzheng; Bai, Fan; Furstenau Togashi, Henrique; Wang, Meng; Zhou, Shuangxi; Ni, Jian (2017): The China Plant Trait Database. PANGAEA, https://doi.org/10.1594/PANGAEA.871819

123. Wenxuan Han, Yahan Chen, Fang-Jie Zhao, Luying Tang, Rongfeng Jiang and Fusuo Zhang, 2012, Floral, climatic and soil pH controls on leaf ash content in China's terrestrial plants. Global Ecology and Biogeography, DOI: 10.1111/j.1466-8238.2011.00677.x

124. Williams, M., Y.E. Shimabokuro and E.B. Rastetter. 2012. LBA-ECO CD-09 Soil and Vegetation Characteristics, Tapajos National Forest, Brazil. Data set. Available on-line [http://daac.ornl.gov] from Oak Ridge National Laboratory Distributed Active Archive Center, Oak Ridge, Tennessee, U.S.A. http://dx.doi.org/10.3334/ORNLDAAC/1104

125. Wilson K., D. Baldocchi, P. Hanson (2000) Spatial and seasonal variability of photosynthetic parameters and their relationship to leaf nitrogen in a deciduous forest. Tree Physiology 20, 565-578

126. Wirth, C. and J. W. Lichstein. 2009. The Imprint of Species Turnover on Old-Growth Forest Carbon Balances - Insights From a Trait-Based Model of Forest Dynamics. Pages 81-113 in C. Wirth, G. Gleixner, and M. Heimann, editors. Old-Growth Forests: Function, Fate and Value. Springer, New York, Berlin, Heidelberg.

127. Wright, I. J., D. D. Ackerly, F. Bongers, K. E. Harms, G. Ibarra-Manriquez, M. Martinez-Ramos, S. J. Mazer, H. C. Muller-Landau, H. Paz, N. C. A. Pitman, L. Poorter, M. R. Silman, C. F. Vriesendorp, C. O. Webb, M. Westoby, and S. J. Wright. 2007. Relationships among ecologically important dimensions of plant trait variation in seven Neotropical forests. Annals of Botany 99:1003-1015.

128. Wright, I. J., P. B. Reich, M. Westoby, D. D. Ackerly, Z. Baruch, F. Bongers, J. Cavender-Bares, T. Chapin, J. H. C. Cornelissen, M. Diemer, J. Flexas, E. Garnier, P. K. Groom, J. Gulias, K. Hikosaka, B. B. Lamont, T. Lee, W. Lee, C. Lusk, J. J. Midgley, M. L. Navas, U. Niinemets, J. Oleksyn, N. Osada, H. Poorter, P. Poot, L. Prior, V. I. Pyankov, C. Roumet, S. C. Thomas, M. G. Tjoelker, E. J. Veneklaas, and R. Villar. 2004. The worldwide leaf economics spectrum. Nature 428:821-827.

129. Wright, S. J., K. Kitajima, N. J. B. Kraft, P. B. Reich, I. J. Wright, D. E. Bunker, R. Condit, J. W. Dalling, S. J. Davies, S. Díaz, B. M. J. Engelbrecht, K. E. Harms, S. P. Hubbell, C. O. Marks, M. C. Ruiz-Jaen, C. M. Salvador, and A. E. Zanne. 2011 . Functional traits and the growth-mortality tradeoff in tropical trees. Ecology 91:3664-3674.

130. Yahan Chen , Wenxuan Han , Luying Tang , Zhiyao Tang and Jingyun Fang 2011 Leaf nitrogen and phosphorus concentrations of woody plants differ in responses to climate, soil and plant growth form. Ecography 34, doi: 10.1111/j.1600-0587.2011.06833.x

131. Yguel B., Bailey R., Tosh N.D., Vialatte A., Vasseur C., Vitrac X., Jean F. & Prinzing A. (2011). Phytophagy on phylogenetically isolated trees: why hosts should escape their relatives. Ecol. Lett., 14, 1117-1124.
