# Supplementary material for: Global relationships in tree functional traits
Source: Nat Commun. 2022 Jun 8;13:3185. doi: 10.1038/s41467-022-30888-2 (PMC9177664; doi:10.1038/s41467-022-30888-2)
Supplement: Supplementary file 4 — Reporting Summary [file 41467_2022_30888_MOESM4_ESM.pdf]

nature portfolio

Corresponding author(s): Daniel S. Maynard

Last updated by author(s): May 6, 2022

Reporting Summary

Nature Portfolio wishes to improve the reproducibility of the work that we publish. This form provides structure for consistency and transparency in reporting. For further information on Nature Portfolio policies, see our Editorial Policies and the Editorial Policy Checklist.

Statistics

For all statistical analyses, confirm that the following items are present in the figure legend, table legend, main text, or Methods section.

n/a	Confirmed	
☐	☒	The exact sample size (*n*) for each experimental group/condition, given as a discrete number and unit of measurement
☐	☒	A statement on whether measurements were taken from distinct samples or whether the same sample was measured repeatedly
☒	☐	The statistical test(s) used AND whether they are one- or two-sided<br>*Only common tests should be described solely by name; describe more complex techniques in the Methods section.*
☐	☒	A description of all covariates tested
☒	☐	A description of any assumptions or corrections, such as tests of normality and adjustment for multiple comparisons
☐	☒	A full description of the statistical parameters including central tendency (e.g. means) or other basic estimates (e.g. regression coefficient) AND variation (e.g. standard deviation) or associated estimates of uncertainty (e.g. confidence intervals)
☒	☐	For null hypothesis testing, the test statistic (e.g. *F*, *t*, *r*) with confidence intervals, effect sizes, degrees of freedom and *P* value noted<br>*Give P values as exact values whenever suitable.*
☒	☐	For Bayesian analysis, information on the choice of priors and Markov chain Monte Carlo settings
☒	☐	For hierarchical and complex designs, identification of the appropriate level for tests and full reporting of outcomes
☐	☒	Estimates of effect sizes (e.g. Cohen's *d*, Pearson's *r*), indicating how they were calculated

Our web collection on statistics for biologists contains articles on many of the points above.

Software and code

Policy information about availability of computer code

Data collection	No specialized software was used for data collection, as all databases were previously compiled and available in tabular text format.
Data analysis	All analysis were conducted in R v. 4.2.0, using specialty packages: dendextend v. 1.15.2, wCorr v. 1.9.5, fastshap v. 0.0.7, aroma.light v. 3.26.0, ape v. 5.6-2, ncf v. 1.2-9, ranger v. 0.13.1, Taxonstand v. 2.4, along with Julia v. 1.6.2, using the Arpack v. 0.5.3 package.

For manuscripts utilizing custom algorithms or software that are central to the research but not yet described in published literature, software must be made available to editors and reviewers. We strongly encourage code deposition in a community repository (e.g. GitHub). See the Nature Portfolio guidelines for submitting code & software for further information.

Data

Policy information about availability of data

All manuscripts must include a data availability statement. This statement should provide the following information, where applicable:

- Accession codes, unique identifiers, or web links for publicly available datasets
- A description of any restrictions on data availability
- For clinical datasets or third party data, please ensure that the statement adheres to our policy

The imputed trait data used in this study have been deposited in a dedicated GitHub repository under accession code DOI: 10.5281/zenodo.6564051 (https://github.com/dsmaynard/tree_traits), along with the cleaned phylogenetic eigenvectors from the seed plant phylogeny of Smith & Brown (2018), and corresponding tree species names matched using BGCI GlobalTreeSearch database v1.3. The raw TRY trait data are not available due to data privacy and sharing restrictions, but can be obtained by making a data request to the TRY Plant Trait Database (https://www.try-db.org/TryWeb/).

Field-specific reporting

Please select the one below that is the best fit for your research. If you are not sure, read the appropriate sections before making your selection.

- [ ] Life sciences
- [ ] Behavioural & social sciences
- [x] Ecological, evolutionary & environmental sciences

For a reference copy of the document with all sections, see nature.com/documents/nr-reporting-summary-flat.pdf

Ecological, evolutionary & environmental sciences study design

All studies must disclose on these points even when the disclosure is negative.

Study description	Using existing trait data from the TRY database, we exploring the trait relationships and environmental drivers among 18 tree traits
Research sample	The goal was to obtain a representative sample of global tree trait expression, and thus we used all available datasets from the TRY database, regardless of location. This included 172 distinct sub-datasets in the TRY database. We used all trait observations that were matched to the reference phylogeny and which were indicated as tree species by BGCI, excluding monocots.
Sampling strategy	As the goal was to approximate the global distribution of tree traits, we did not do any sub-sampling of the data, but rather used all available trait/species data in the TRY database.
Data collection	Data were obtained from the TRY database in April 2020, with the final collection including all available datasets of the requested traits, in tabular form. The final analysis used 172 different sub-datasets from the TRY database. Meta-data providing collection protocols is not provided in the TRY database, but the sub-datasets were screened to ensure similar units and comparable collection methods within each trait (Supplementary Table S3).
Timing and spatial scale	We used all available data, regardless of sampling date (which is generally not available in TRY). As the goal is to estimate trait relationships worldwide, we also did not restrict our data spatially, and instead use all observations from across the globe.
Data exclusions	To focus on trees vs. woody shrubs, we excluded observations less than 5m in height, 1m in crown width or height, 10 cm diameter, and 25 cm root depth. Individual outliers also identified if they were un-biological and could not be externally verified (e.g., stem diameter > 10 m).
Reproducibility	The code and imputed traits in the GitHub repository allow for complete reproduction of the main figures and results. Reproducibility of the trait imputations was assessed via out-of-fit cross-validation, along with exploration of the sensitivity of the results to variable number of covariates in the models; and restriction to species with between 2 and 10 traits.
Randomization	There was no randomization of the data, as the study design was not experimental but rather observational/correlative, and so no direct statistical tests or comparisons of groups/treatments was conducted that required randomization.
Blinding	As the study design was not experimental, there was no blinding of the data, and there were no direct statistical tests or comparisons of groups/treatments that required blinding.

Did the study involve field work? [ ] Yes [x] No

Reporting for specific materials, systems and methods

We require information from authors about some types of materials, experimental systems and methods used in many studies. Here, indicate whether each material, system or method listed is relevant to your study. If you are not sure if a list item applies to your research, read the appropriate section before selecting a response.

Materials & experimental systems

n/a	Involved in the study	
[x]	[ ]	Antibodies
[x]	[ ]	Eukaryotic cell lines
[x]	[ ]	Palaeontology and archaeology
[x]	[ ]	Animals and other organisms
[x]	[ ]	Human research participants
[x]	[ ]	Clinical data
[x]	[ ]	Dual use research of concern

Methods

n/a	Involved in the study	
[x]	[ ]	ChIP-seq
[x]	[ ]	Flow cytometry
[x]	[ ]	MRI-based neuroimaging
